# The combined effect of water deficit stress and TiO_2_ nanoparticles on cell membrane and antioxidant enzymes in *Helianthus annuus* L.

**DOI:** 10.1007/s12298-022-01153-z

**Published:** 2022-03-15

**Authors:** Taha Ramadan, Suzan A. Sayed, Amna K. A. Abd-Elaal, Ahmed Amro

**Affiliations:** Department of Botany and Microbiology, Faculty of Science, Assiut University, Assiut, 71516 Egypt

**Keywords:** *Helianthus annuus* L., TiO_2_, Membrane injury, Water deficit, Antioxidant enzymes, Proline

## Abstract

Nanotechnology has become one of the several approaches attempting to ameliorate the severe effect of drought on plant's production and to increase the plants tolerance against water deficit for the water economy. In this research, the effect of foliar application of TiO_2_, nanoparticles or ordinary TiO_2_, on *Helianthus annuus* subjected to different levels of water deficit was studied. Cell membrane injury increased by increasing the level of water deficit and TiO_2_ concentration, and both types of TiO_2_ affected the leaves in analogous manner. Ord-TiO_2_ increased H_2_O_2_ generation by 67–240% and lipid peroxidation by 4–67% in leaves. These increases were more than that induced by Nano-TiO_2_ and the effect was concentration dependent. Proline significantly increased in leaves by water deficit stress, reaching at 25% field capacity (FC) to more than fivefold compared to that in plants grown on full FC. Spraying plants with water significantly decreased the activities of enzymes in the water deficit stressed roots. The water deficit stress exerted the highest magnitude of effect on the changes of cell membrane injury, MDA, proline content, and activities of CAT and GPX. Nano-TiO_2_ was having the highest effect on contents of H_2_O_2_ and GPX activity. In roots, the level of water deficit causes highest effect on enzyme activities, but TiO_2_ influenced more on the changes of MDA and H_2_O_2_ contents. GPX activity increased by 283% in leaves of plants treated with 50 and 150 ppm Nano-TiO_2_, while increased by 170% in those treated with Ord-TiO_2_, but APX and CAT activities increased by 17–197%, in average, with Ord-TiO_2_. This study concluded that Nano-TiO_2_ didn’t ameliorate the effects of drought stress on *H. annuus* but additively increased the stress, so its use in nano-phytotechnology mustn’t be expanded without extensive studies.

**Supplementary Information:**

The online version contains supplementary material available at 10.1007/s12298-022-01153-z.

## Introduction

The response of plants to water deficit stress is complex and involves changes in their macro- and micro-morphology and all physiological processes. Water deficit stress, as most abiotic stresses, leads to accumulation of reactive oxygen species (ROS), generated mostly in chloroplast and mitochondria, causing oxidative stress. Amongst the defense responses against abiotic stresses, plants tend to activate their ROS scavenging mechanism (Vranová et al. 2002). Therefore, plants intrinsically develop different types of antioxidants reducing oxidative damage and conferring stress tolerance. From these types are the antioxidant enzymes which include superoxide dismutase, peroxidase, and catalase (Siddiqui and Khan 2018; Khan et al. 2020). Water deficit stress causes oxidative damage in plants which results in the disruption of photosynthetic apparatus, results in decreased chlorophyll content (Shivakrishna et al. 2018).

Titanium has significant biological effect on plants, being beneficial at low levels but toxic at higher concentrations. Variations in  titanium's effect on plants will depend on the differences in environmental factors, diversity of plant species and the applied concentrations of titanium (Feizi et al. 2012; Tan et al. 2018; Wahid et al. 2020). Similar to other nanomaterials, the properties and concentration of Nano-TiO_2_ have important roles in its application. On the contrary, several reports have presented the harmful effects of a high concentration of TiO_2_ on plants that varied between plant tissues, growth stages and plant species (Nair et al. 2010; Feizi et al. 2012; Zulfiqar and Ashraf 2021). Therefore, properties, concentration, method of application, uptake by plants, reactivity and translocation of nanoparticles into different plant tissues could determine their interference with various physiological activities that lead to toxic impacts **(**Mattiello et al. 2015; Rastogi et al. 2017; Paramo et al. 2020). Furthermore, surface area of nanoparticles, their reactivity and tendency for aggregation are additional possible reasons for their harmful effects (Kobayashi et al. 2019). High concentrations of Nano-TiO_2_ mainly elevate generation of ROS, followed by chlorophyll degradation and cellular toxicity (Rico et al. 2015). In addition, damage of the cell walls and plasma membranes by high nanoparticles concentration result in an interaction with various cellular process **(**Rastogi et al. 2017; Hou et al. 2019). Sompornpailin et al. (2020) showed that application of titanium nanoparticles increased the cell membrane stability, photosynthetic pigments, and antioxidant activities in *Nicotiana tabacum*. Khan et al. (2020) also showed that Nano-TiO_2_ increased the production of enzyme and non-enzyme antioxidants in *Vicia faba* subjected to a shortage in water supply.

Hong et al. (2005) and Liu et al. (2021) reported an increase in the activities of SOD, CAT, POD and a decrease in accumulation of ROS when plants were exposed to TiO_2_ nanoparticles. Mohammadi et al. (2016) concluded that foliar treatment of dragonhead plant with high concentrations of Nano-TiO_2_ increased injurious effects of water deficit stress on physiological processes due to increasing the levels of lipid peroxidation and H_2_O_2_ generation, and decreasing the stability of photosynthetic pigments. Therefore, extensive use of Nano-TiO_2_ in order to diminish adverse effects of water deficit stress even in the case of economic crops need further investigation. Besides, the influence of Nano-TiO_2_ residues on the environment and human health should also be considered and elucidated.

Servin et al. (2013) reported that CAT activity increased in cucumber plants grown in soil amended with 250–750 mg Nano-TiO_2_ kg^−1^ but the activity of APX decreased at 500 mg kg^−1^. Also, Laware and Raskar (2014) reported that CAT and GPX activities were enhanced in the presence of 10–30 μg ml^−1^, but their activities decreased by higher concentrations of Nano-TiO_2_. Lei et al. (2008) showed that Nano-TiO_2_ treatment activated SOD, CAT, APX and GPX of spinach chloroplasts. According to Harrison (1996), the exogenous application of Nano-TiO_2_ increased CAT activity and plant resistance to environmental stresses. The APX is thought to play the most essential role in scavenging ROS and protecting cells in higher plants, algae, euglena and other organisms. APX is involved in scavenging H_2_O_2_ in water-water and ASH-GSH cycles and utilizes ASH as the electron donor (Ahmad et al. 2019; Kohli et al. 2019).

Recently, nanotechnology has been a subject of great interest in various fields including botany. Titanium dioxide (Nano-TiO_2_) is among the most highly manufactured and widely used nanoparticles in the world (Jomini et al. 2015). Due to the extensive use and applications, these nanomaterials have already entered the agroecosystems leading to exposure of plants to Nano-TiO_2_. As titanium enter the food chains, the human exposure to these nanomaterials will be increased. Therefore, evaluation of the toxic effects induced by Nano-TiO_2_ and its effects on the human health was reviewed by some authors (Iavicoli et al. 2012; Shah et al. 2017). Though, this is not the objective of this study, it is worthwhile to mention that the International Agency for Research on Cancer (IARC) classified TiO_2_ in 2006 and reassessed it in 2010 as “possibly carcinogenic to humans”. In this study, we addressed four questions. First, does the foliar application of Nano-TiO_2_ exert the same effect as Ord-TiO_2_ on sunflower plants? Second, how increasing concentrations of TiO_2_ affect the cell membranes and some enzyme activity? Third, can Nano-TiO_2_ ameliorate the effect of drought to some extent? Fourth, does Nano-TiO_2_ have a combined effect with drought as abiotic stressor on plants? As the TiO_2_ is applied by foliar spraying, whether or not the root will be affected similarly as the shoot under different levels of water availability was another objective for this study.

## Materials and methods

### Experimental design and treatments

This experiment was carried out to study the effect of different concentrations of nanoparticles or ordinary titanium dioxide (Nano-TiO_2_ or Ord-TiO_2_) on cell membrane integrity and some antioxidant enzymes of *Helianthus annuus* L. (sunflower) plants subjected to different levels of water availability. Seeds of sunflower (cultivar Giza 102) were obtained from the Agronomy Department, Faculty of Agriculture, Assiut University, Assiut, Egypt. The experiment started on 26- September 2019 at the Green house of the Botany Department, Assiut University under field conditions. Through September, October and November, the averages of maximum temperature was 32 ± 2, minimum temperature was 18 ± 1.7, and relative humidity was 31% ± 1. About 15 seeds were planted in pots containing 3 kg of soil (2:1 clay: sand by weight). The pots (120 pots) were irrigated by tap water to the field capacity which was determined to be about 24% of dry soil. After about one month from cultivation, the number of individuals was fixed to 7 homogeneous plants per pot. To avoid nutrient impoverishment, each pot received 50 ml one tenth strength of Hoagland’s solution, 10 ml with irrigation water day after day.

A factorial experiment based on randomized complete block design was carried out with three replications. The 120 pots were divided into 10 groups, three pots as replicates of four water levels (100%, 75%, 50% and 25% of field capacity), the welting point of sunflower was 7%. The first group was the control, the second group was foliar sprayed with distilled water and the other 8 groups were sprayed with 50, 150, 300 and 600 ppm Nano-TiO_2_ or Ord-TiO_2_. The foliar spraying was applied after the plants established under different water levels for 10 days, where the plants were with about three pairs of leaves. Each pot was sprayed twice at 5 days interval with a total of 15 ml spraying solution. After 25 days from the first spraying (the plants were with 6 pairs of leaves), the plants were harvested for different measurements.

The Nano-TiO_2_ with particle size less than 25 nm (Fig. S1), purity 99.7% and surface area of 45–55 m^2^ g^−1^ was purchased from Sigma-Aldrich Company. Nano-TiO_2_ and Ord-TiO_2_ particles, separately, were dissolved in distilled water just before using and scattered by ultrasonic vibration “BANDELIN SONOPULS HD 2070” homogenizer at 100 W and 40 kHz for 10 min.

### Membrane stability index and electrolyte leakage:

Cell membrane stability was carried out as given by Premachandra et al. (1992). Fresh leaf discs (10 discs) were rinsed three times with double distilled water, then submerged in 25 ml double distilled water for 24 h at room temperature. The electric conductivity of the bathing solution was measured using conductometer (YSI Model 35 Yellow Springs, OH, USA). Then samples were autoclaved and the EC measured again after cooling to the room temperature. Cell membrane stability index, or membrane injury, was evaluated as percentage injury according to the following equation:

$${\text{Membrane}}\,\,{\text{injury}}\,\,{\text{index}} = { }\left[ {1 - \frac{{\left( {1 - {\raise0.7ex\hbox{${{\text{T}}_{1} }$} \!\mathord{\left/ {\vphantom {{{\text{T}}_{1} } {{\text{T}}_{2} }}}\right.\kern-\nulldelimiterspace} \!\lower0.7ex\hbox{${{\text{T}}_{2} }$}}} \right)}}{{\left( {1 - {\raise0.7ex\hbox{${{\text{C}}_{1} }$} \!\mathord{\left/ {\vphantom {{{\text{C}}_{1} } {{\text{C}}_{2} }}}\right.\kern-\nulldelimiterspace} \!\lower0.7ex\hbox{${{\text{C}}_{2} }$}}} \right)}}{ }} \right]{ } \times 100$$

In addition, the electrolyte leakage was calculated relative to that of control plants as following:

$${\text{Electrolyte}}\,\,{\text{leakage}} = {{\frac{{{\text{T}}_{1} }}{{{\text{T}}_{2} }}} \mathord{\left/ {\vphantom {{\frac{{{\text{T}}_{1} }}{{{\text{T}}_{2} }}} {\frac{{{\text{C}}_{1} }}{{{\text{C}}_{2} }}}}} \right. \kern-\nulldelimiterspace} {\frac{{{\text{C}}_{1} }}{{{\text{C}}_{2} }}}}{ }$$

where T_1_ and T_2_ are EC values of the treated plants, while C_1_ and C_2_ represent the EC values of control plants before and after autoclaving, respectively.

### Determination of lipid peroxidation (malondialdehyde content)

The level of lipid peroxidation in sunflower leaves was determined as malondialdehyde (MDA) content according to the method of Hodges et al. (1999) with minor modifications**.** Content of MDA, which is an end product of lipid peroxidation, was determined using the thiobarbituric acid reaction. 0.2 g fresh leaves was homogenized with 4 ml ethanol (80%v/v) containing 2% dimethyl sulfoxide, and the homogenate was centrifuged at 10,000 rpm for 10 min. For every one ml aliquot, 3 ml of 20% TCA containing 0.65% thiobarbituric acid (TBA) was added. The mixture was heated at 95 °C for 30 min, then cooled quickly on an ice-bath. After that, the mixture was centrifuged at 6000 rpm for 15 min and the absorbance of the supernatant was measured at 532 nm using UV2000/2200 Spectrophotometer (Ray Wild Limited Compony, Germany). Blanks with the same weight from the same leaf or opposite one were proceeded as the samples but without TBA. After subtracting blank reading, the level of lipid peroxidation was expressed as nmol g^−1^ FW of MDA formed using an extinction coefficient of 155 mM^−1^ cm^−1^.

#### Determination of H_2_O_2_

The hydrogen peroxide content in the leaves or roots of sunflower was spectrophotometrically measured as described by Sellers (1980) with minor modifications. A defined weight (0.1 g) of the tissue was immediately extracted with 2 ml cold acetone followed by 3 ml double distilled water. After centrifugation at 6000 rpm for 10 min, 3 ml from the supernatant was mixed with 1 ml of 0.1 M potassium titanium (IV) oxalate dihydrate [K_2_TiO(C_2_O_4_)_2_.·2H_2_O] in 5 M sulphuric acid. The intensity of yellow-orange color was measured at 400 nm. A blank was prepared by boilling the same weight of leaves or roots for one minute and proceeded as the fresh sample. The molar absorptivity 935 L mol^−1^ cm^−1^ was used in calculation of H_2_O_2_ as μmole g^−1^ FW.

#### Determination of proline

Proline concentration was determined using the acid–ninhydrin method according to Bates et al. (1973). Exactly, 0.2 g fresh leaves were homogenized in 3 ml of 3% sulfosalicylic acid, then the homogenate was centrifuged for 10 min. at 10,000 rpm. One ml of the mixture was reacted with 400 µl acid-ninhydrin and 400 µl of glacial acetic acid and 200 µl of 3% sulfosalicylic acid in a test tube for one hour at 96 °C, and the reaction was terminated in an ice bath. The reaction mixture was extracted with 4 ml toluene and mixed vigorously. The chromophore containing toluene was aspirated from the aqueous phase, warmed to room temperature and the absorbance at 520 nm was measured using toluene as a blank. The proline concentration was determined from a standard curve and calculated as mg g^−1^ FW.

### Assay of antioxidant enzymes activity

#### Preparation of enzyme extract

Root or leaf tissues (0.5 g) were ground to a fine powder in liquid N_2_, and then homogenized in 5 ml of 100 mM potassium phosphate buffer (pH 7.8) containing 0.1 mM ethylenediamine tetraacetic acid-disodium salt (Na_2_-EDTA) and 0.1 g polyvinylpyrrolidone (PVP). The homogenate was centrifuged at 18,000 rpm for 10 min at 4 °C and the supernatants collected and used for the assay of catalase, guaiacol peroxidase and ascorbate peroxidase. All colorimetric measurements were made at 20 °C using spectrophotometer. The specific activity was expressed as units mg^−1^ protein min^−1^.

Catalase (CAT; EC 1.11.1.6) activity was determined by measuring the rate of H_2_O_2_ dissociation to O_2_ and water for one minute according to modified method of Aebi (1984). The assay medium (3 ml) consisted 2.8 ml 50 mM potassium phosphate buffer (pH 7), 100 µl of enzyme extract and the reaction was initiated by addition of 100 µl 10 mM H_2_O_2_. The decrease in absorbance at 240 nm was recorded for one minute.

Guaiacol peroxidase (GPX; EC 1.11.1.7) activity was measured using modification of the procedure of Zaharieva et al. (1999). Guaiacol was used as the substrate. Peroxidase activity was measured in a reaction mixture (1.3 ml) that contained 100 μl enzyme extract, 100 μl 6.5 mM H_2_O_2_, and 100 μl 1.5 mM guaiacol in 1 ml 30 mM phosphate buffer (pH = 7). The formation of tetraguaiacol was measured at 470 nm.

Ascorbate peroxidase (APX; EC 1.11.1.11) activity was measured according to Nakano and Asada (1981) by monitoring the rate of ascorbate oxidation at 290 nm. The reaction mixture contained 1.6 ml of 50 mM potassium phosphate buffer (pH = 7), 0.1 mM Na_2_-EDTA, 5 mM H_2_O_2_, 0.5 mM ascorbic acid and 50 µl enzyme extract. The decrease in absorbance at 290 nm was monitored to calculate the activity of APX.

### Statistical analysis

Data were subjected to statistical analysis using SPSS (version 21). One-way ANOVA was performed following when the effect was significant at *P* ≤ 0.05, by the post hoc Duncan’s multiple-range test (at *P* ≤ 0.05) for comparison between means of parameters at each level of water availability or at each concentration of the spraying solution. Two-way ANOVA was carried to achieve the effect of water availability, Nano- or Ord-TiO_2_ and their interaction on different parameters estimated in leaves or roots and eta square “η^2^” was calculated as: η^2^ = SS_Effect_/SS_Total_ to achieve the size effects of each factor or the interaction between factors. In addition, Pearson correlation analysis was performed to obtain the relation between some parameters.

## Results

### Effect of nano- and ord-TiO_2_ on the cell membranes of sunflower

**Fig. 1:**
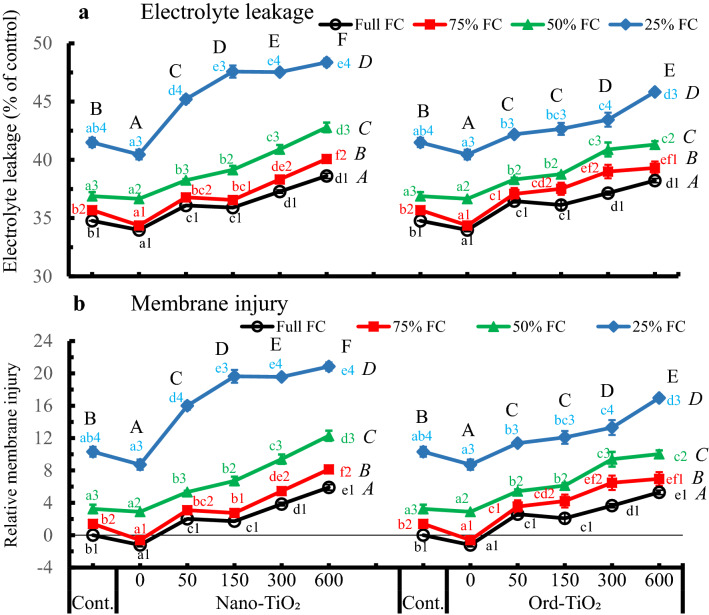
Electrolyte leakage (**a**) and cell membrane injury (**b**) in leaves of sunflower subjected to different levels of water availability (full, 75%, 50% and 25% FC) when the plants were 40 days old and sprayed with Nano- or Ord-TiO_2_ (0, 50, 150, 300 and 600 ppm) after 10 days from exposure to the levels of FC (the plants were with 3 pairs of true leaves). Electrolyte leakage and cell membrane injury were estimated when the plants were bearing 6 pairs of leaves (≈ 75 days old). Data are means ± SE, n = 3. For comparison between different concentrations of TiO_2_ at each FC level, means in each line labelled by similar lower case letter(s) are not significantly different; but for comparison between different FC levels at each concentration, means with similar figures are not significantly different according to One-Way ANOVA and Duncan's Multiple Range Test at *P* ≤ 0.05. Upper case letters are for comparison between groups of water deficit levels (italic letters on the right side of FC levels, n = 18), or between groups of Nano- or Ord-TiO_2_ treatments (upper case letters above treatments, n = 12) according to Two-Way ANOVA and Duncan's Multiple Range Test at *P* ≤ 0.05

Electrolyte leakage and cell membrane injury of sunflower leaves (Fig. 1a, b) were matching, following the same trend, and both were decreased by spraying plants with water, then increased gradually and mostly significantly by either Nano- or Ord-TiO_2_ or by decreasing the levels of water availability. Electrolyte leakage increased significantly in plants sprayed with Nano- or Ord-TiO_2_, and the leakage was more pronounced with Nano-TiO_2_ treatments (Fig. 1a). The damage by spraying plants with 600 ppm Nano-TiO_2_ was magnified by the shortage in water supply reaching to 2-, 4- and sixfold at 75%, 50% and 25% FC, respectively, compared to that at full FC. The cell membranes were significantly injured by lowering water availability to 50% and 25% FC. In plants grown on 25% FC and sprayed with 150 ppm Nano-TiO_2_, the membrane injury was magnified to 11-fold of that at full FC. In comparison, the membrane damage was generally lower by Ord-TiO_2_ treatments, especially at the lowest level of water availability (Fig. 1B). The highest recorded membrane damage or electrolyte leakage by spraying plants grown on 25% FC with 600 ppm Nano-TiO_2_ did not exceed significantly compared to those of 150 and 300 ppm Nano-TiO_2_ at the same water level. Based on the two-way ANOVA, all concentrations of TiO_2_ and all levels of FC significantly affected the electrolyte leakage and membrane injury of sunflower leaves. Only, 50 and 150 ppm Ord-TiO_2_ were having similar effects (Fig. 1). The membrane injury of sunflower leaves was significantly correlated with the contents of H_2_O_2_ either by spraying with Nano-TiO_2_ (*r*-value = 0.891**) or with Ord-TiO_2_ (*r*-value = 0.844**).

### Effect on H_2_O_2_ generation, lipid peroxidation and proline accumulation

**Fig. 2:**
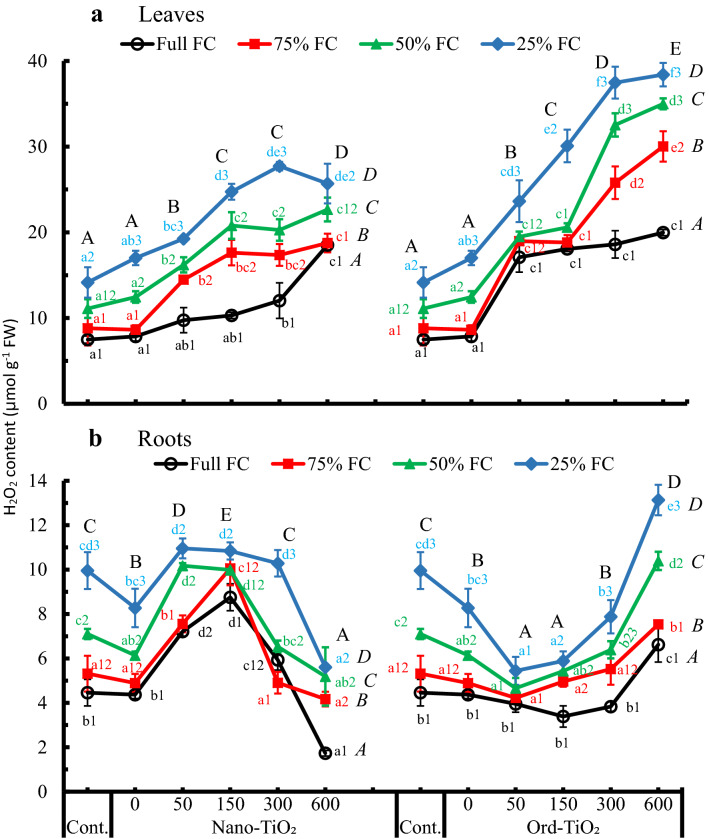
Content of hydrogen peroxide (μmol H_2_O_2_ g^−1^ FW) in leaves (**a**) and roots (**b**) of sunflower subjected to different levels of water availability when the plants were 40 days old and sprayed with Nano- or Ord-TiO_2_ after 10 days from exposure to the levels of FC (the plants were with 3 pairs of true leaves). After 25 days from spraying, the plants (≈ 75 days old) were bearing 6 pairs of leaves. H_2_O_2_ was determined in the 2^nd^ leaf from the top. Data are means ± SE, n = 3. For comparison between different concentrations of TiO_2_ at each FC level, means in each line labelled by similar lower case letter(s) are not significantly different; but for comparison between different FC levels at each concentration, means with similar figures are not significantly different according to One-Way ANOVA and Duncan's Multiple Range Test at *P* ≤ 0.05. Upper case letters are for comparison between groups of water deficit levels (italic letters on the right side of FC levels, n = 18), or between groups of Nano- or Ord-TiO_2_ treatments (upper case letters above treatments, n = 12) according to Two-Way ANOVA and Duncan's Multiple Range Test at *P* ≤ 0.05

Data illustrated in Fig. 2a showed that leaf H_2_O_2_ increased continuously with decreasing water availability at all treatments of TiO_2_. In leaves of plants grown on full FC, increasing concentration of the spraying solution (Nano- or Ord-TiO_2_) did not induce significant differences in H_2_O_2_ generation, except at 600 ppm Nano-TiO_2_. At 25% FC, H_2_O_2_ content increased significantly in leaves of plants treated with Nano-TiO_2_ up to 300 ppm, then decreased by spraying plants with 600 ppm Nano-TiO_2_. The maximum H_2_O_2_ content was observed in roots of most plants treated with 50 and 150 ppm Nano-TiO_2_. In contrast, H_2_O_2_ in the leaves increased by increasing the concentration of Ord-TiO_2_. At full FC, H_2_O_2_ content did not change significantly in plants treated with Ord-TiO_2_. Contents of H_2_O_2_ in sunflower leaves sprayed with Ord-TiO_2_ were generally more compared to those sprayed with Nano-TiO_2_. At the lower level of water availability (25% FC), H_2_O_2_ in plants treated with 300 and 600 ppm Ord-TiO_2_ increased to 2.5–3.5-fold compared to that in the control (Fig. 2A).

Compared to leaves, H_2_O_2_ in sunflower roots showed different trend toward decreasing by 300 and 600 ppm Nano-TiO_2_, and this was opposite for the same treatments of Ord-TiO_2_ (Fig. 2b). Roots H_2_O_2_ significantly increased up to 150 ppm Nano-TiO_2_ then reduced gradually till 600 ppm. An opposite trend was achieved with Ord-TiO_2_, i.e., the lowest H_2_O_2_ content was detected at 50 and 150 ppm of Ord-TiO_2_, while the highest content was observed at 600 ppm for all levels of water availability. Compared to control, H_2_O_2_ in roots of plants sprayed with 600 ppm Nano-TiO_2_ was reduced by 40–80%, but was increased to 130–150% by 600 ppm Ord-TiO_2_. The results of two-way ANOVA revealed to significant differences between the levels of each main factor. In roots, change in the content of H_2_O_2_ by increasing the water deficit stress was depending on the concentration of TiO_2_.

**Fig. 3:**
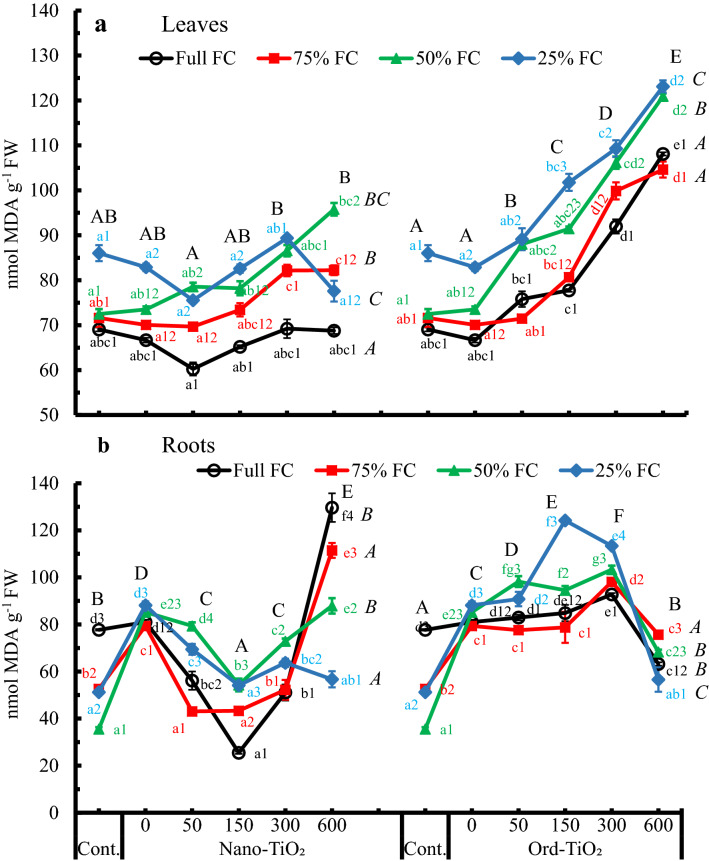
Lipid peroxidation (nmol MDA g^−1^ FW) in leaves (**a**) and roots (**b**) of sunflower subjected to different levels of water availability when the plants were 40 days old and sprayed with Nano- or Ord-TiO_2_ after 10 days from exposure to the levels of FC (the plants were with 3 pairs of true leaves). After 25 days from spraying, the plants (≈ 75 days old) were bearing 6 pairs of leaves. MDA was determined in the 2^nd^ leaf from the top. Data are means ± SE, n = 3. For comparison between different concentrations of TiO_2_ at each FC level, means in each line labelled by similar lower case letter(s) are not significantly different; but for comparison between different FC levels at each concentration, means with similar figures are not significantly different according to One-Way ANOVA and Duncan's Multiple Range Test at *P* ≤ 0.05. Upper case letters are for comparison between groups of water deficit levels (italic letters on the right side of FC levels, n = 18), or between groups of Nano- or Ord-TiO_2_ treatments (upper case letters above treatments, n = 12) according to Two-Way ANOVA and Duncan's Multiple Range Test at *P* ≤ 0.05

Generally, foliar spraying of sunflower plants with Ord-TiO_2_ increased H_2_O_2_ generation (Fig. 2a) and concomitantly the lipid peroxidation in its leaves (Fig. 3a) more than Nano-TiO_2_. Also, the level of H_2_O_2_ or lipid peroxidation increased by the concentration of TiO_2_ (Nano or Ord) treatment. The maximum levels of H_2_O_2_ and lipid peroxidation were shown in plants treated with 600 ppm Ord-TiO_2_. The minimum content of MDA was detected in plants treated with 50 ppm Nano-TiO_2_, while the maximum content was observed in plants treated with 600 ppm Ord-TiO_2_. However, the MDA content in sunflower leaves were significantly correlated with the contents of H_2_O_2_ either by spraying with Nano-TiO_2_ (*r*-value = 0.726**) or with Ord-TiO_2_ (*r*-value = 0.915**), but the correlation was negative in the roots with *r*-values = − 0.578* and − 0.408, respectively.

Lipid peroxidation of leaf cells was generally increased proportionally and significantly with decreasing soil–water potential (i.e. from Full FC to 25% FC), especially by treatment with Ord-TiO_2_ (Fig. 3a). This trend was different with high concentrations of Nano-TiO_2_, i.e., lipid peroxidation was unchanged from 300 to 600 ppm Nano-TiO_2_ in plants grown on Full and 75% FC, while it was reduced by 600 ppm Nano-TiO_2_ in plants grown on 25% FC. At all levels of water availability, spraying plants with 50 and 150 ppm Nano-TiO_2_ did not induce any significant difference in MDA. In contrast, 300 and 600 ppm Ord-TiO_2_ increased lipid peroxidation process to 130–170% compared to control, especially at 50% FC.

As in leaves, lipid peroxidation of the root cells of sunflower was the worst at 25% and 50% FC, compared to plants grown on 75% and full FC (Fig. 3B). At this shortage of water supply, lipid peroxidation increased by 2–3 fold compared to control. The levels of water availability 50% and above showed the peak of lipid peroxidation by 600 ppm Nano-TiO_2_. On the other hand, 600 ppm Ord-TiO_2_, compared to the other applied concentrations, significantly decreased the content of MDA at all levels of water availability. Comparing between groups by two-way ANOVA showed that there are non-significant differences between the effects of Nano-TiO_2_ concentrations above 50 ppm on the lipid peroxidation in leaves, while there are significant differences between all treatments of Ord-TiO_2_ (Fig. 3a). In roots, the effect of low concentrations of either Nano- or Ord-TiO_2_ on lipid peroxidation was significantly different from that of high concentrations (Fig. 3b).

**Fig. 4:**
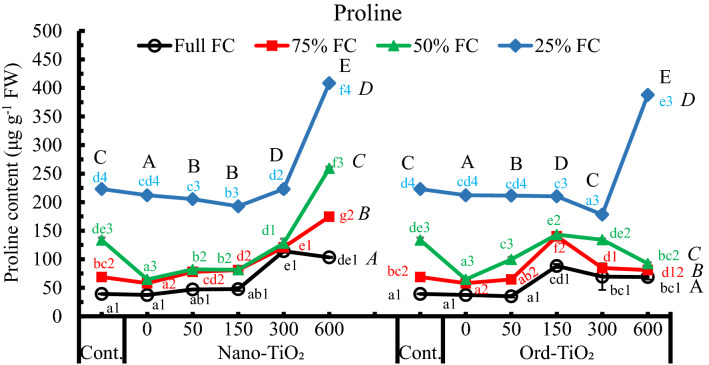
Content of proline (µg g^−1^ FW) in leaves of sunflower subjected to different levels of water availability when the plants were 40 days old and sprayed with Nano- or Ord-TiO_2_ after 10 days from exposure to the levels of FC (the plants were with 3 pairs of true leaves). After 25 days from spraying, the plants (≈ 75 days old) were bearing 6 pairs of leaves. Proline was determined in the 2^nd^ leaf from the top. Data are means ± SE, n = 3. For comparison between different concentrations of TiO_2_ at each FC level, means in each line labelled by similar lower case letter(s) are not significantly different; but for comparison between different FC levels at each concentration, means with similar figures are not significantly different according to One-Way ANOVA and Duncan's Multiple Range Test at *P* ≤ 0.05. Upper case letters are for comparison between groups of water deficit levels (italic letters on the right side of FC levels, n = 18), or between groups of Nano- or Ord-TiO_2_ treatments (upper case letters above treatments, n = 12) according to Two-Way ANOVA and Duncan's Multiple Range Test at *P* ≤ 0.05

Changes of proline contents in leaves of sunflower plants responded differentially to TiO_2_ concentrations and water availability (Fig. 4). The proline content in leaves of plants grown under shortage of water supply, whether sprayed with TiO_2_ or not, increased significantly in comparison with plants grown under full FC. Proline dramatically increased in plants grown on 25% available water reaching more than fivefold compared to that in plants grown on full FC. On the other hand, concentrations of Nano-TiO_2_ up to 150 ppm exerted weak changes in proline content that increased significantly at 300 and 600 ppm. In addition, plants sprayed with Nano-TiO_2_, especially 600 ppm, accumulated more proline compared to those sprayed with Ord-TiO_2_. In plants grown on 75% and 50% FC, despite the proline content was increased significantly by 600 ppm Nano-TiO_2_, it was decreased by Ord-TiO_2_. However, the adverse effect of water deficit stress on sunflower, as indicated by proline accumulation, is exacerbated by the highest concentration of TiO_2_ where the combined effect of both main factors is higher compared to either factor alone.

### Antioxidant enzymes

**Fig. 5:**
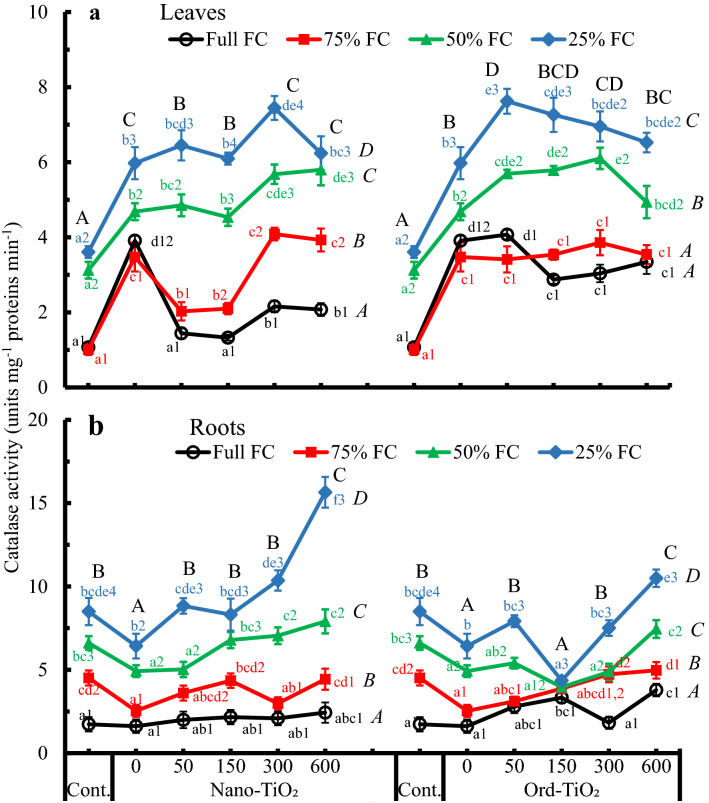
Catalase activity (unit mg^−1^ protein min^−1^) in leaves (**a**) and roots (**b**) of sunflower subjected to different levels of water availability when the plants were 40 days old and sprayed with Nano- or Ord-TiO_2_ after 10 days from exposure to the levels of FC (the plants were with 3 pairs of true leaves). After 25 days from spraying, the plants (≈ 75 days old) were bearing 6 pairs of leaves. The extract in which the enzyme activity was assayed was obtained from leaves that originated after spraying with TiO_2_. Data are means ± SE, n = 3. For comparison between different concentrations of TiO_2_ at each FC level, means in each line labelled by similar lower case letter(s) are not significantly different; but for comparison between different FC levels at each concentration, means with similar figures are not significantly different according to One-Way ANOVA and Duncan's Multiple Range Test at *P* ≤ 0.05. Upper case letters are for comparison between groups of water deficit levels (italic letters on the right side of FC levels, n = 18), or between groups of Nano- or Ord-TiO_2_ treatments (upper case letters above treatments, n = 12) according to Two-Way ANOVA and Duncan's Multiple Range Test at *P* ≤ 0.05

To evaluate if Nano- or Ord-TiO_2_ have a role in the activity of antioxidant enzymes, catalase (CAT), guaiacol peroxidase (GPX) and ascorbate peroxidase (APX) activities were assayed in leaves and roots of sunflower plants. Results of CAT activity are illustrated in Fig. 5. Catalase activity significantly differed by water availability and TiO_2_ material. The activity in leaves (Fig. 5a) gradually and significantly increased from full field capacity (Full FC) toward dryness (25% FC) under both types of TiO_2_ treatments. This increasing activity by drought, especially at 25% FC, was relatively suppressed by high TiO_2_ concentrations (300 and 600 ppm). The activity of CAT increased significantly in plants sprayed with 50 and 150 ppm Ord-TiO_2_ compared to control or those sprayed with Nano-TiO_2_.

**Fig. 6:**
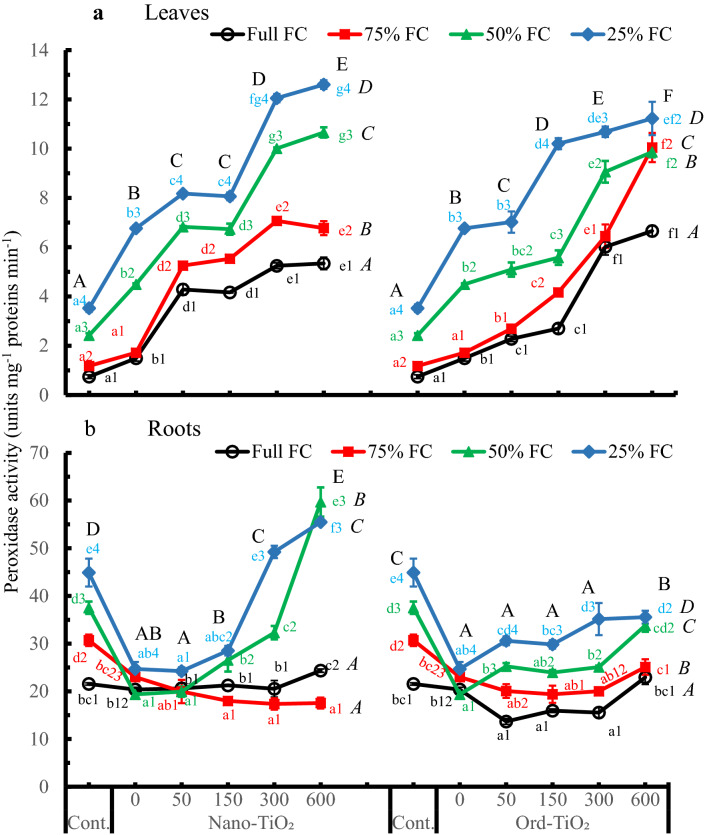
Guaiacole peroxidase activity (unit mg^−1^ protein min^−1^) in leaves (**a**) and roots (**b**) of sunflower subjected to different levels of water availability when the plants were 40 days old and sprayed with Nano- or Ord-TiO_2_ after 10 days from exposure to the levels of FC (the plants were with 3 pairs of true leaves). After 25 days from spraying, the plants (≈ 75 days old) were bearing 6 pairs of leaves. The extract in which the enzyme activity was assayed was obtained from leaves that originated after spraying with TiO_2_. Data are means ± SE, n = 3. For comparison between different concentrations of TiO_2_ at each FC level, means in each line labelled by similar lower case letter(s) are not significantly different; but for comparison between different FC levels at each concentration, means with similar figures are not significantly different according to One-Way ANOVA and Duncan's Multiple Range Test at *P* ≤ 0.05. Upper case letters are for comparison between groups of water deficit levels (italic letters on the right side of FC levels, n = 18), or between groups of Nano- or Ord-TiO_2_ treatments (upper case letters above treatments, n = 12) according to Two-Way ANOVA and Duncan's Multiple Range Test at *P* ≤ 0.05

Generally, in roots (Fig. 5b), spraying plants with water significantly decreased the CAT activity and the other TiO_2_ levels showed, with minor exceptions, slight or no changes compared to that of control. Notably there was unusual similarity in CAT activity at 150 ppm Ord-TiO_2_, i.e., there was no difference between the CAT activity among water availability gradients. The results in Fig. 5 showed that CAT activity is affected by changing water availability more than by changing the concentrations of spraying TiO_2_ solutions. Also, at all levels of water supply, by spraying plants with water the CAT activity increased significantly in leaves but decreased in roots. As indicated by two-way ANOVA, the CAT (Fig. 5a) and GPX (Fig. 6a) activity in leaves increased significantly by all levels of both main factors. In roots, the activity increased significantly by 50% and 25% levels of FC, and by only the highest concentration of Nano-TiO_2_, but all concentrations of Ord-TiO_2_ significantly decreased the CAT activity. The activity of GPX increased in sunflower leaves by increasing the concentration of Nano- or Ord-TiO_2_. In contrast to catalase, the GPX activity increased in plants treated with Nano-TiO_2_ more than in plants treated with Ord-TiO_2_.

The activity of GPX in sunflower leaves increased continuously and most significantly from control to 600 ppm of both types of TiO_2_ treatments at all levels of water availability. Therefore, the highest activity was observed in plants treated with 300 and 600 ppm Nano-TiO_2_ (Fig. 6). This increase, in leaves of plants grown on full FC, reached by nanoparticles treatment to about sevenfold, while it reached to about fourfold in plants grown on 50% and 25% FC. The same trend was shown for Ord-TiO_2_, but with less magnitude at 50% and 25% FC. The results in Fig. 6a refer to non-significant differences between the effect of 50 and 150 ppm or between 300 and 600 ppm Nano-TiO_2_. Mostly, at all treatments of TiO_2_, whether in leaves or roots, as the available water decreases the GPX activity increases.

Activity of GPX in roots followed approximately different trend from that of leaves (Fig. 6b). Generally, there was non-significant change in GPX activity by increasing TiO_2_ concentration especially of Ord-TiO_2_. Except in plants grown on 50% and 25% FC and sprayed with 300 and 600 ppm Nano-TiO_2_, the activity of GPX was inhibited in sunflower roots compared to control. This reduction reached to 53% and 63% of control in plants grown on 50% FC and sprayed with 50 ppm Nano-TiO_2_ and 150 ppm Ord-TiO_2_, respectively. Otherwise, unusual increase was observed in GPX activity by 600 ppm Nano-TiO_2_ treatment in roots of plants grown on 50% and 25% FC.

**Fig. 7:**
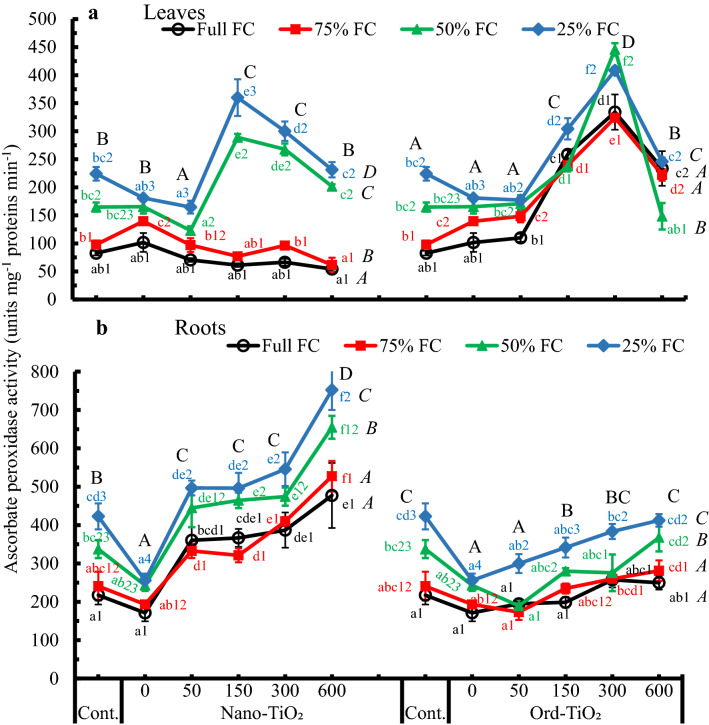
Ascorbate peroxidase activity (unit mg^−1^ protein min^−1^) in leaves (**a**) and roots (**b**) of sunflower subjected to different levels of water availability when the plants were 40 days old and sprayed with Nano- or Ord-TiO_2_ after 10 days from exposure to the levels of FC (the plants were with 3 pairs of true leaves). After 25 days from spraying, the plants (≈ 75 days old) were bearing 6 pairs of leaves. The extract in which the enzyme activity was assayed was obtained from leaves that originated after spraying with TiO_2_. Data are means ± SE, n = 3. For comparison between different concentrations of TiO_2_ at each FC level, means in each line labelled by similar lower case letter(s) are not significantly different; but for comparison between different FC levels at each concentration, means with similar figures are not significantly different according to One-Way ANOVA and Duncan's Multiple Range Test at *P* ≤ 0.05. Upper case letters are for comparison between groups of water deficit levels (italic letters on the right side of FC levels, n = 18), or between groups of Nano- or Ord-TiO_2_ treatments (upper case letters above treatments, n = 12) according to Two-Way ANOVA and Duncan's Multiple Range Test at *P* ≤ 0.05

Data illustrated in Fig. 7 showed that APX activity increased in sunflower leaves treated with Ord-TiO_2_ more than in those treated with Nano-TiO_2_. In plants grown on full and 75% FC, APX activity did not change by Nano-TiO_2_ treatments, but increased with Ord-TiO_2_. At all treatments, the activity of APX in leaves (Fig. 7a) or roots (Fig. 7b) increased by decreasing the water availability. However, APX activity increased in plants subjected to low soil–water potentials (at 50% and 25% FC) compared to those subjected to high water potentials (Full and 75% FC). This increasing activity was more pronounced in plants treated by Nano-TiO_2_ compared to those treated by Ord-TiO_2_. Compared to control plants or those sprayed by water, APX activity unchanged in the leaves treated with 50 ppm Ord-TiO_2,_ but increased significantly by the higher concentrations.

Roots APX release due to TiO_2_ treatments occurred after a notable reduction by spraying plants with water (Fig. 7b). However, its activity increased dramatically by increasing the level of Nano-TiO_2_. The APX activity in the sunflower roots due to Ord-TiO_2_ treatments was changing within a narrow range at all levels of water availability, so the activity did not differ statistically compared to control. Also, the effect of drought stress (25% FC) was clearer on the enzyme activity in plant roots under all TiO_2_ treatments. It is worthy to mention that foliar spraying on plants with water decreased the APX activity in the roots compared to control, and the decreases were significant at 50% and 25% FC. Data of two-way ANOVA revealed to a significant increase in the APX activity in leaves and roots of sunflower by 50% and 25% FC. The activity increased significantly by all concentrations of Nano-TiO_2_ in roots and by 150 and 300 ppm in leaves.

When averaged across all levels of water availability, the data in Table 1 showed that membrane injury, proline, and also the activity of CAT and GPX in sunflower leaves sprayed with Nano-TiO_2_ mostly did not differ significantly from those sprayed with Ord-TiO_2_. In addition, lipid peroxidation, H_2_O_2_ and APX activity increased significantly by most concentrations of Ord-TiO_2_ compared to Nano-TiO_2_. Also, most measured parameters (averaged across all levels of FC) in sunflower leaves increased significantly by increasing concentration of Nano- or Ord-TiO_2_. Interestingly, the contents of H_2_O_2_ increased but MDA decreased in the plant roots by Nano-TiO_2_ up to 300 ppm, the situation was different at 600 ppm. Except MDA in roots of plants sprayed with Nano- or Ord-TiO_2_, all measured parameters (averaged across all concentrations of TiO_2_) increased significantly and gradually by shortage in water availability. The content of H_2_O_2_ increased significantly by 50 and 150 ppm Nano-TiO_2_ compared to control group or other treatments.

**Table 1:** The effect of concentrations of Nano- or Ord-TiO_2_ on the studied parameters in leaves and roots of *H. annuus* grown on different levels of water deficit stress

Organ	TiO_2_ Treatment	Mem. injury			H_2_O_2_ (μmol g^−1^ FW)			MDA (nmol g^−1^ FW)			Proline (μg g^−1^ FW)		
		NPs	Ord	Sign.	NPs	Ord.	Sign.	NPs	Ord	Sign.	NPs	Ord.	Sign.
Leaves	Full FC	2.1^a^ ± 0.57	2.1^a^ ± 0.5	ns	11.0^a^ ± 1.0	14.8 ^a^ ± 2.2	*	66.5^a^ ± 1.6	81.6^a^ ± 6.4	**	64.9^a^ ± 7.7	56.4^a^ ± 5.9	ns
	75% FC	3.4^b^ ± 0.7	3.7^b^ ± 0.7	ns	14.3^b^ ± 1.1	18.5^b^ ± 3.4	ns	74.9^b^ ± 1.8	83.1^a^ ± 6.3	*	96.9^b^ ± 9.7	82.9^b^ ± 6.7	ns
	50% FC	6.6^c^ ± 0.8	6.2^c^ ± 0.7	ns	17.3^c^ ± 1.1	21.9^c^ ± 5.6	ns	80.9^bc^ ± 3.3	92.1^b^ ± 7.7	ns	124.9^c^ ± 15.9	111.3^c^ ± 6.9	ns
	25% FC	15.9^d^ ± 1.2	12.1^d^ ± 0.7	**	21.4^d^ ± 1.3	26.8^d^ ± 6.6	ns	82.3^c^ ± 2.2	98.7^c^ ± 6.4	**	244.1^d^ ± 18	237.1^d^ ± 16.7	ns
	Control	3.7^b^ ± 1.2	3.7^b^ ± 1.2		10.4^a^ ± 1.0	10.4^a^ ± 1.0		74.8^ab^ ± 3.5	74.8^a^ ± 3.5		116.2^c^ ± 21.3	116.2^c^ ± 21.3	
	H_2_O	2.5^a^ ± 1.2	2.5^a^ ± 1.2		11.5^a^ ± 1.1	11.5^a^ ± 1.1		73.3^ab^ ± 2.5	73.3^a^ ± 2.5		93.0^a^ ± 20.9	93.0^a^ ± 20.9	
	50 ppm	6.6^c^ ± 1.7	5.7^c^ ± 1.0	ns	14.9^b^ ± 1.1	19.8^b^ ± 1.0	**	71.0^a^ ± 2.8	81.1^b^ ± 2.4	*	103.2^b^ ± 18.3	102.7^b^ ± 20.2	ns
	150 ppm	7.7^d^ ± 2.2	6.1^c^ ± 1.2	ns	18.4^c^ ± 1.7	21.9^c^ ± 1.5	ns	74.9^ab^ ± 2.9	87.9^c^ ± 3.3	**	100.7^b^ ± 16.6	145.5^d^ ± 13.1	*
	300 ppm	9.6^e^ ± 1.9	8.2^d^ ± 1.1	ns	19.4^c^ ± 1.8	28.6^d^ ± 2.3	**	81.8^b^ ± 3.4	101.8^d^ ± 2.4	**	146.5^d^ ± 13.6	116.6^c^ ± 13.9	ns
	600 ppm	11.8^f^ ± 1.7	9.8^e^ ± 1.4	ns	21.4^d^ ± 1.1	30.9^e^ ± 2.2	**	81.1^b^ ± 4.1	114.2^e^ ± 2.7	**	236.4^e^ ± 34.3	157.5^e^ ± 40.2	ns
Roots	Full FC				5.4^a^ ± 0.6	4.4^a^ ± 0.3	ns	70.2^b^ ± 7.9	80.4^b^ ± 2.2	ns			
	75% FC				6.2^b^ ± 0.5	5.4^b^ ± 0.3	ns	63.7^a^ ± 6.0	77.0^a^ ± 3.4	ns			
	50% FC				7.5^c^ ± 0.5	6.7^c^ ± 0.5	ns	69.2^b^ ± 4.6	80.9^b^ ± 5.6	ns			
	25% FC				9.3^d^ ± 0.5	8.4^d^ ± 0.7	ns	63. 9^a^ ± 3.1	87.4^c^ ± 6.6	**			
	Control				6.7^c^ ± 0.7	6.7^c^ ± 0.7		54.3^b^ ± 4.6	54.3^a^ ± 4.6				
	H_2_O				5.9^b^ ± 0.5	5.9^b^ ± 0.5		83.4^d^ ± 1.2	83.4^c^ ± 1.2				
	50 ppm				9.0^d^ ± 0.5	4.6^a^ ± 0.3	**	62.0^c^ ± 4.3	87.4^d^ ± 2.5	**			
	150 ppm				9.9^e^ ± 0.3	4.9^a^ ± 0.3	**	44.3^a^ ± 3.6	95.6^e^ ± 2.5	**			
	300 ppm				6.9^c^ ± 0.6	5.9^b^ ± 0.5	ns	60.0^c^ ± 3.0	101.9^f^ ± 2.3	**			
	600 ppm				4.2^a^ ± 0.5	9.4^d^ ± 0.8	**	96.4^e^ ± 8.4	65.9^b^ ± 2.4	**			

Organ	TiO_2_ Treatment	CAT			GPX			APX					
		Activity (units mg^−1^ proteins min−)											
		NPs	Ord.	Sign.	NPs	Ord.	Sign.	NPs	Ord.	Sign.			
Leaves	Full FC	2.0 ^a^ ± 0.2	3.1^a^ ± 0.2	**	3.6^a^ ± 0.4	3.3^a^ ± 0.5	ns	72.8^a^ ± 4.6	186.8^a^ ± 23.8	**			
	75% FC	2.8^b^ ± 0.3	3.1^a^ ± 0.3	ns	4.6^b^ ± 0.6	4.4^b^ ± 0.8	ns	94.9^b^ ± 6.5	195.3^a^ ± 18.5	**			
	50% FC	4.8^c^ ± 0.2	5.1^b^ ± 0.3	ns	6.9^c^ ± 0.7	6.1^c^ ± 0.6	ns	202.0^c^ ± 14.6	222.3^b^ ± 25.7	ns			
	25% FC	6.0^d^ ± 0.3	6.3^c^ ± 0.3	ns	8.5^d^ ± 0.8	8.2^d^ ± 0.7	ns	243.5^d^ ± 17.5	256.9^c^ ± 19.8	ns			
	Control	2.2^a^ ± 0.4	2.2^a^ ± 0.4		2.0^a^ ± 0.3	2.0^a^ ± 0.3		142.4^b^ ± 17.4	142.4^a^ ± 17.4				
	H_2_O	4.5^c^ ± 0.3	4.5^b^ ± 0.3		3.6^b^ ± 0.7	3.6^b^ ± 0.7		147.0^b^ ± 10.2	147.0^a^ ± 10.2				
	50 ppm	3.7^b^ ± 0.6	5.2^d^ ± 0.5	ns	6.1^c^ ± 0.5	4.3^c^ ± 0.6	*	113.8^a^ ± 11.2	151.6^a^ ± 9.0	*			
	150 ppm	3.5^b^ ± 0.6	4.9^bcd^ ± 0.5	ns	6.1^c^ ± 0.4	5.7^d^ ± 0.9	ns	196.8^c^ ± 39.9	260.1^c^ ± 9.5	ns			
	300 ppm	4.8^c^ ± 0.6	5.0^cd^ ± 0.5	ns	8.6^d^ ± 0.8	8.1^e^ ± 0.6	ns	182.6^c^ ± 31.3	378.2^d^ ± 17.0	**			
	600 ppm	4.5^c^ ± 0.5	4.6^bc^ ± 0.4	ns	8.8^e^ ± 0.9	9.5^f^ ± 0.5	ns	137.3^b^ ± 24.5	212.6^b^ ± 14.4	*			
Roots	Full FC	2.0^a^ ± 0.2	2.5^a^ ± 0.2	ns	21.5^a^ ± 0.45	18.3^a^ ± 0.9	**	329.9^a^ ± 29.6	214.9^a^ ± 9.7	**			
	75% FC	3.7^b^ ± 0.2	4.0^b^ ± 0.3	ns	21.1^a^ ± 1.2	23.0^b^ ± 1.1	ns	337.5^a^ ± 27.9	230.5^a^ ± 11.8	**			
	50% FC	6.4^c^ ± 0.3	5.5^c^ ± 0.3	ns	32.6^b^ ± 3.4	27.5^c^ ± 1.5	ns	436.5^b^ ± 32.5	282.2^b^ ± 17.2	**			
	25% FC	9.7^d^ ± 0.8	7.5^d^ ± 0.5	*	37.8^c^ ± 3.1	33.5^d^ ± 1.7	ns	494.9^c^ ± 38	352.5^c^ ± 16.7	**			
	Control	5.3^b^ ± 0.8	5.3^b^ ± 0.8		33.7^d^ ± 2.7	33.7^c^ ± 2.7		304.6^b^ ± 27.8	304.6^c^ ± 27.8				
	H_2_O	3.9^a^ ± 0.6	3.9^a^ ± 0.6		21.9^ab^ ± 0.7	21.9^a^ ± 0.7		215.8^a^ ± 12.5	215.8^a^ ± 12.5				
	50 ppm	4.9^b^ ± 0.8	4.8^b^ ± 0.6	ns	21.2^a^ ± 0.8	22.4^a^ ± 1.9	ns	408.6^c^ ± 24.3	214.3^a^ ± 17.0	**			
	150 ppm	5.4^b^ ± 0.8	3.9^a^ ± 0.2	ns	23.6^b^ ± 1.4	22.3^a^ ± 1.6	ns	412.1^c^ ± 24.3	263.7^b^ ± 17.6	**			
	300 ppm	5.6^b^ ± 1.0	4.7^b^ ± 0.6	ns	29.9^c^ ± 3.8	23.9^a^ ± 2.3	ns	454.2^c^ ± 23.7	294.1^bc^ ± 19.9	**			
	600 ppm	7.6^c^ ± 1.6	6.7^c^ ± 0.8	ns	39.3^e^ ± 5.6	29.4^b^ ± 1.7	ns	603.0^d^ ± 40.3	327.7^c^ ± 22.5	**			

The data are averages across all concentrations of TiO_2_ in each level of water deficit (n = 18), and across all levels of water deficit iin control, H_2_O-sprayed group and each concentration of TiO_2_ (n = 12). Comparison between Nano- and Ord-TiO_2_ at each level of FC or concentration of TiO_2_ was achieved from one-way ANOVA, while the comparison between different levels of each factor (water availability or TiO_2_) was achieved from two-way ANOVA and Duncan’s test at *P* ≤ 0.05

For comparison between different levels of water deficit or different concentrations of TiO_2_ either in leaves or roots, averages with different letters are significantly different according to Duncan’s test

* and ** = significant at *P* ≤ 0.05 and 0.01, respectively; ns = non-significant

To measure the effect size of each factor, η^2^ was calculated by dividing the sums of squares for the effecting factor (SS_effect_) on the total sums of squares for all effects including errors and interactions (SS_total_) in the ANOVA. Values of η^2^ in Table 2 showed that the shortage in water availability exerted the highest magnitude of effect on the changes of studied parameters in sunflower leaves treated with Nano- or Ord-TiO_2_ except the content of H_2_O_2_ and GPX activities where Nano-TiO_2_ was having the highest effect. Compared to NPs, Ord-TiO_2_ exerted the highest effect on changing lipid peroxidation, H_2_O_2_, GPX and APX activities in the leaves, while the shortage in water availability exerted the highest magnitude of effect on the changes of other parameters. In roots of sunflower treated with Nano- or Ord-TiO_2_, the levels of water availability mostly wielded the highest effect on enzyme activities, but TiO_2_ influenced more on the changes of MDA and H_2_O_2_ contents. The values of η^2^ revealed that the effect of interaction between different levels of water availability and Nano- or Ord-TiO_2_ is weak or negligible on all parameters either in leaves or roots. Based on the two-way ANOVA, the two main factors (water deficit stress, Nano- or Ord-TiO_2_) and their interaction significantly affected approximately all measured parameters in leaves and roots of sunflower.

**Table 2:** The eta squared ($\eta$^2^) for the magnitude of effect of Nano- (NPs) or Ord-TiO_2_ (OPs), water deficit stress (WD) and the interaction between them on changes of each studied parameter in sunflower leaves and roots. The significant effect of each factor on the parameters is achieved from the two-way ANOVA

Organ	Water deficit stressed plants sprayed with	Factor	Eta square ($\eta$^2^)													
			Memb. injury	Sign	H_2_O_2_	Sign	Lipid perox	Sign	Proline	Sign	CAT activity	Sign	GPX activity	Sign	APX activity	Sign
Leaves	Nano-TiO_2_	NPs	0.250	***	0.456	***	0.120	*	0.318	***	0.212	***	0.587	***	0.104	***
		WD	0.706	***	0.408	***	0.292	***	0.596	***	0.673	***	0.362	***	0.674	***
		WD*NPs	0.036	***	0.054	***	0.118	ns	0.083	***	0.078	***	0.046	***	0.188	***
		Error	0.008		0.082		0.470		0.003		0.037		0.004		0.034	
	Ord-TiO_2_	OPs	0.285	***	0.681	***	0.709	***	0.079	***	0.317	***	0.613	***	0.783	***
		WD	0.674	***	0.221	***	0.158	***	0.735	***	0.590	***	0.322	***	0.082	***
		WD*OPs	0.014	***	0.059	***	0.024	ns	0.176	***	0.046	***	0.049	***	0.093	***
		Error	0.027		0.039		0.109		0.010		0.048		0.016		0.042	
Roots	Nano-TiO_2_	NPs			0.522	***	0.563	***			0.108	***	0.299	***	0.649	***
		WD			0.322	***	0.016	***			0.724	***	0.351	***	0.213	***
		WD*NPs			0.072	***	0.399	***			0.113	***	0.322	***	0.029	***
		Error			0.085		0.022				0.055		0.028		0.108	
	Ord-TiO_2_	OPs			0.430	***	0.679	***			0.167	***	0.320	***	0.295	***
		WD			0.382	***	0.035	***			0.636	***	0.509	***	0.457	***
		WD*OPs			0.090	***	0.261	***			0.125	***	0.111	***	0.075	ns
		Error			0.098		0.025				0.073		0.060		0.173	

*, ** and *** = significant at *P* ≤ 0.05, 0.01 and 0.001, respectively; ns = non-significant

## Discussion and conclusion

Water deficiency is one of the most severe environmental stresses which affects almost all plant functions. One of the inevitable consequences of water deficit stress is the enhancement of ROS generation in the chloroplasts, peroxisomes and mitochondria of the plant cell. To keep the intracellular ROS level and set the redox-status of the cell, the enhanced ROS generation is mainly kept under tight control by a cooperative antioxidant systems. The enhanced ROS itself function as an alarm signal that triggers defense responses by specific signal transduction pathways that involve H_2_O_2_ as secondary messenger (Cruz de Carvalho 2008; Ahmad et al. 2019). In this study, the shortage of water availability significantly increased electrolyte leakage and cell membrane injury, H_2_O_2_ content and lipid peroxidation (which is a key indicator of oxidative stress) in sunflower plants. As the content of ROS increases, the damage of cellular membranes increases. In sunflower subjected to water deficit stress, an increase in thylakoid membrane electron leakage to O_2_ was observed by Sgherri et al. (1996). However, it is difficult to assess the fraction of ROS generated due to electron leakage from thylakoid membrane from that generated by photorespiration which affect strongly on the oxidative load under drought stress. Noctor et al. (2002) estimated quantitatively the photorespiration oxidative load in the leaves of C_3_-plants and observed that about 70% of total H_2_O_2_ is generated by photorespiration under drought stress.

Titanium dioxide (Nano-TiO_2_) is among the most often used nanoparticles in various fields of nano-phytotechnology. Because it has been used extensively, the exposure of plants to Nano-TiO_2_ will be inevitable and concomitantly its transport through the food chains. Larue et al. (2012) reported that foliar application of nanoparticles can enter the leaves through stomatal apertures and then are translocated to various tissues via the symplast and/or apoplast pathways. In leaves of sunflower, TiO_2_ whether nanoparticles or ordinary showed additive effect with shortage of water availability by injuring the cell membranes and increasing electrolyte leakage, lipid peroxidation, H_2_O_2_ generation and proline accumulation. However, the adverse effect of water deficit stress on sunflower plants that was significant (especially under severe shortage in water availability) was aggravated by Nano-TiO_2_, and there combination was having a greater detrimental effect compared to either water deficit or nanoparticles separately. The effect was concentration dependent. Wang et al. (2010) observed that the Nano-TiO_2_ particles caused an increase in lipid peroxidation of treated plants. Larue et al. (2012) reported that the mode of action of plant cell damage in certain concentrations of TiO_2_ nanoparticles is unknown.

Both Nano- and Ord-TiO_2_ differentially affected the contents of H_2_O_2_ and MDA in the leaves and roots of sunflower, where both were positively and negatively correlated in the leaves and roots, respectively. This was due to the decreasing levels of H_2_O_2_ in roots that did not exceed 12 μmole g^−1^ FW, while in leaves it was ranging between 8 and 38 μmole g^−1^ FW. In addition, the activities of assayed enzymes CAT, GPX and APX increased in roots by water deficit stress, Nano- or Ord-TiO_2_. The two main factors, levels of FC and concentrations of TiO_2_, and their interaction affected significantly on all measured parameters in leaves and roots of sunflower. Hydrogen peroxide generated in the plant cell organelles such as chloroplast, mitochondria, but mainly in peroxisomes. Normally, this ROS is going to be balanced between productions and scavenging in all cell sections. Sometimes this balance is disturbed by a number of contrary environmental stressors, including drought and unwanted metals associated with oxidative stress (Gupta and Sandalio 2012).

Lei et al. (2008) reported that the application of Nano-TiO_2_ enhanced the activities of rubisco and antioxidant enzyme activities, photosynthetic rate and chlorophyll formation that subsequently increased crop yield. TiO_2_ could be considered as a stimulant for plants through the activation of different defense mechanisms involved in plant tolerance against various abiotic stresses. Zheng et al. (2005) showed that the growth of spinach plants was greatly improved at 250–4000 ppm Nano-TiO_2_, but no improvement was observed at higher concentrations when they used higher concentrations up to 8000 ppm. Regarding the negative effect of Nano-TiO_2_, Foltete et al. (2011) observed that these nanoparticles can attach to the *Vicia faba* root surface in 48 h upon exposure and subsequently inhibited the plant growth. The data of this study indicate that TiO_2_ is a stressor at all concentrations, but the effect was concentration dependent. In addition, no stimulation of the growth parameters was observed by spraying sunflower plants with TiO_2_ (Fig. S2). Shoemaker (2020) studied the effect of up to 1000 mg Nano-TiO_2_ kg^−1^ soil on *Sorghum bicolor* and found no significant effect on physiology or biomass accumulation. He stated that further research will be necessary to identify if Nano-TiO_2_ particles penetrate the leaf cuticle and epidermis and translocated by the plant.

The results in this study showed that the magnitude of effect of water deficit stress in increasing the content of proline and enzyme activities is generally higher compared to that of TiO_2_. Ashraf and Foolad (2007) reported that proline has a strong ability to hydrate, so it can play a protective role in cell structure. When the plant is challenged by adverse abiotic factors, proline accumulate and interacts with proteins to form a hydrophobic skeleton to stabilize and protect biological macromolecules and cell membrane structures. Proline is also a variety of free radical scavengers, and can reduce the ROS damage through chelating singlet oxygen and hydroxyl radical. Another way of proline to remove ROS is to stimulate the activity of peroxidase, catalase and other enzymes in plants (Yang et al. 2021). In this study, TiO_2_, either ordinary or nanoparticles, seemed to be not belonging to events that enhance proline accumulation in plants especially by treatments with low concentrations. The higher concentrations of TiO_2_ when combined with the high level of water deficit affected significantly on proline accumulation. Our results contradict to those of Shah et al. (2021),  who observed an enhanced accumulation of proline in salinized maize seedlings by priming its caryopses with TiO_2_, despite proline content reduced by salinity stress.

In contrast to proline, the magnitude of effect of Nano- or Ord-TiO_2_ on increasing the generation of H_2_O_2_ was higher compared to that of drought. There are numerous studies reporting the induction of oxidative stress by water deficit (Morán et al. 1994; Dat et al. 2000; Wang et al. 2016). The activities of antioxidative enzymes, like superoxide dismutase, peroxidase and catalase, were found to be correlated with tolerance to drought stress (Dat et al. 2000; Bartels and Sunkar 2005; Wang et al. 2016). Also, Mittler and Zilinskas (1994) observed that the activities of chloroplastic and cytosolic Cu, Zn-SODs and cytosolic APX increased during drought of pea plants. However, Dŏgarŏglu (2017) documented no effect of Nano-TiO_2_ on the activities of antioxidant enzymes in wheat seedlings.

In this study, although the shortage in water availability and foliar application of TiO_2_ exerted combined diverse effect on sunflower, both seem to be capable of enhancing the antioxidative enzyme defense reactions in the plants. Generally, the related antioxidative defense reactions including catalase capable of scavenging hydrogen peroxide are also activated. In leaves and roots of sunflower, despite the activity of CAT, GPX and APX increased by decreasing water availability, the activity varied according to the concentration of TiO_2_ treatment solution. In addition the trend of activity was different from one enzyme to another. This may be due to the difference in the existing compartments of each enzyme in the cells. These enzymes are located throughout different compartments of the plant cell, with the exception of CAT that is exclusively located in peroxisomes. Its main function is to remove H_2_O_2_ produced by photorespiration or fatty acid β-oxidation reaction (Willekens et al. 1997). However, as H_2_O_2_ can easily diffuse across the cell membranes, H_2_O_2_ generated by other parts of the cell can also be diffused into peroxisomes and decomposed by CAT. So, CAT is responsible for scavenging H_2_O_2_ produced in or diffused into peroxisomes. The increase in activity of catalase, which was in the peroxisomes of plants, may be a manifestation of the adaptive response of plants to abiotic stress (Leung 2018). APX which needs a reductant (ascorbate) to scavenge H_2_O_2_ has a high affinity for H_2_O_2_ than CAT. APX is located in every cellular ROS producing compartment (Mittler 2002). This may explain why Nano- or Ord-TiO_2_ affected APX activity in leaves and roots of sunflower in a different manner from that on CAT or GPX. The increase in APX activity in sunflower roots by high concentrations of Nano-TiO_2_ was accompanied by a decrease in H_2_O_2_ generation. This is documenting to the hypothesis that, APX may function as a fine regulator of intracellular ROS steady-state levels, possibly at signaling levels, whereas CAT may function mainly as a bulk scavenger for excess ROS generation under stress conditions (Willekens et al. 1997; Noctor and Foyer 1998; Mittler 2002). In leaves of sunflower, combination of high concentration of Nano-TiO_2_ with high levels of water deficit inhibited the activity of APX. The combination of high levels of two or more stressors may damage some of the antioxidative systems in plants.

Unfortunately, there are many conflicting opinions and results about the effect of Nano-TiO_2_ on the seed germination, growth parameters or physiological and enzyme activities of plants. Therefore, it is difficult to find an answer in the literature to the question whether or not application of Nano-TiO_2_ is improving to the plants. According to the concentration applied, the particles size used, the target plant species and the environmental conditions, the effect of nano-TiO_2_ varied from biostimulation to death of the cells and plants. Although membrane injury, lipid peroxidation and H_2_O_2_ generation increased in sunflower leaves and roots by water deficit stress, they increased more by Nano- or Ord-TiO_2_, and concomitantly the activity of antioxidant enzymes enhanced. In conclusion, the foliar spraying of sunflower plants with Nano-TiO_2_ did not ameliorate the stress caused by decreasing water availability, on the other hand it increased the stress and its use in nano-phytotechnology mustn’t be expanded without extensive studies.

## Supplementary Information

Below is the link to the electronic supplementary material.

Supplementary file1 (DOCX 2992 KB)

Supplementary file2 (PDF 1949 KB)
