# Supplementary material for: The combined effect of water deficit stress and TiO2 nanoparticles on cell membrane and antioxidant enzymes in Helianthus annuus L
Source: Physiol Mol Biol Plants. 2022 Mar 15;28(2):391–409. doi: 10.1007/s12298-022-01153-z (PMC8943097; doi:10.1007/s12298-022-01153-z)
Supplement: Supplementary file 1 — Supplementary file1 (DOCX 2992 KB) [file 12298_2022_1153_MOESM1_ESM.docx]

Fig. S1: TEM micrograph of TiO_2_ nanoparticles. The quantified particles size is < 25 nm.


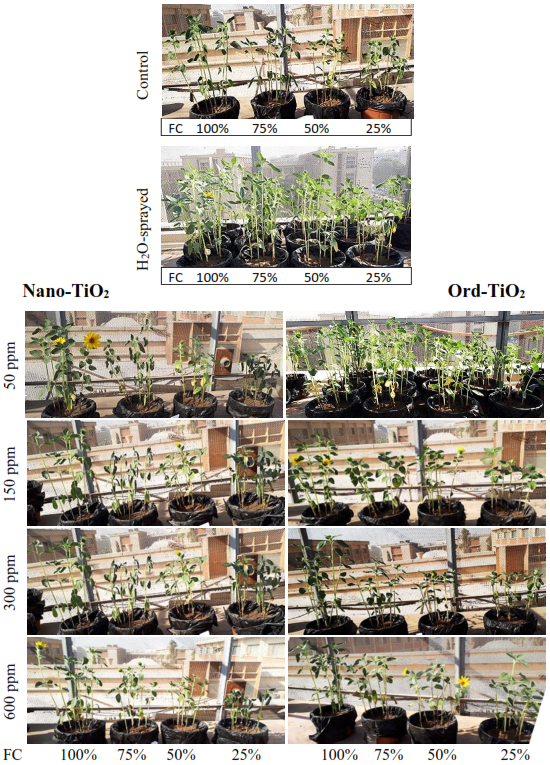
Fig. S2: Photos of *Helianthus annuus* subjected to different levels of water availability (100, 75, 50 and 25% of field capacity) and sprayed with different concentrations of Nano- or Ord-TiO_2_.
