# Supplementary material for: The combined effect of water deficit stress and TiO2 nanoparticles on cell membrane and antioxidant enzymes in Helianthus annuus L
Source: Physiol Mol Biol Plants. 2022 Mar 15;28(2):391–409. doi: 10.1007/s12298-022-01153-z (PMC8943097; doi:10.1007/s12298-022-01153-z)
Supplement: Supplementary file 2 — Supplementary file2 (PDF 1949 KB) [file 12298_2022_1153_MOESM2_ESM.pdf]

```
UNIANOVA EC_Leakage BY FC_Levels NPs
  /METHOD=SSTYPE(3)
  /INTERCEPT=EXCLUDE
  /SAVE=SEPRED
  /POSTHOC=FC_Levels NPs(DUNCAN)
  /PLOT=PROFILE(NPs*FC_Levels)
  /EMMEANS=TABLES(OVERALL)
  /EMMEANS=TABLES(FC_Levels)
  /EMMEANS=TABLES(NPs)
  /EMMEANS=TABLES(FC_Levels*NPs)
  /PRINT=DESCRIPTIVE
  /CRITERIA=ALPHA(.05)
  /DESIGN=FC_Levels NPs FC_Levels*NPs.
```

Univariate Analysis of Variance

[DataSet1] E:\Amna M Sc\Paper\Two Way ANOVA H.sav

Between-Subjects Factors

		Value Label	N
FC_Levels	1	FFC	18
	2	75%FC	18
	3	50%FC	18
	4	25%FC	18
NPs	1	Cont	12
	2	Water sprayed	12
	3	50NP	12
	4	150NP	12
	5	300NP	12
	6	600NP	12

Descriptive Statistics

Dependent Variable: EC_Leakage

FC_Levels	NPs	Mean	Std. Deviation	N
FFC	Cont	34.7667	.00577	3
	Water sprayed	33.9733	.23965	3
	50NP	36.0833	.04726	3
	150NP	35.8967	.01155	3
	300NP	37.2733	.24028	3
	600NP	38.6167	.38991	3
	Total	36.1017	1.58479	18
75%FC	Cont	35.6800	.05568	3
	Water sprayed	34.3700	.03606	3
	50NP	36.7833	.23159	3
	150NP	36.5533	.49238	3
	300NP	38.3200	.49487	3
	600NP	40.0767	.22279	3
	Total	36.9639	1.90214	18
50%FC	Cont	36.8867	.59341	3
	Water sprayed	36.6500	.12530	3
	50NP	38.2467	.16773	3
	150NP	39.1400	.58966	3
	300NP	40.9000	.65023	3
	600NP	42.7867	.70939	3
	Total	39.1017	2.28457	18
25%FC	Cont	41.4967	.69974	3
	Water sprayed	40.4600	.72021	3
	50NP	45.2100	.55435	3
	150NP	47.5767	.90235	3
	300NP	47.5300	.12530	3
	600NP	48.3667	.57848	3
	Total	45.1067	3.22368	18
Total	Cont	37.2075	2.73134	12
	Water sprayed	36.3633	2.71087	12
	50NP	39.0808	3.79426	12
	150NP	39.7917	4.88853	12
	300NP	41.0058	4.18489	12
	600NP	42.4617	3.91291	12
	Total	39.3185	4.21192	72

Tests of Between-Subjects Effects

Dependent Variable: EC_Leakage

Source	Type III Sum of Squares	df	Mean Square	F	Sig.
Model	112557.370[a]	24	4689.890	22446.380	.000
FC_Levels	889.958	3	296.653	1419.815	.000
NPs	314.356	5	62.871	300.909	.000
FC_Levels * NPs	45.214	15	3.014	14.427	.000
Error	10.029	48	.209		
Total	112567.399	72			

a. R Squared = 1.000 (Adjusted R Squared = 1.000)

Estimated Marginal Means

1. Grand Mean

Dependent Variable: EC_Leakage

Mean	Std. Error	95% Confidence Interval	
		Lower Bound	Upper Bound
39.318	.054	39.210	39.427

2. FC_Levels

Dependent Variable: EC_Leakage

FC_Levels	Mean	Std. Error	95% Confidence Interval	
			Lower Bound	Upper Bound
FFC	36.102	.108	35.885	36.318
75%FC	36.964	.108	36.747	37.181
50%FC	39.102	.108	38.885	39.318
25%FC	45.107	.108	44.890	45.323

3. NPs

Dependent Variable: EC_Leakage

NPs	Mean	Std. Error	95% Confidence Interval	
			Lower Bound	Upper Bound
Cont	37.208	.132	36.942	37.473
Water sprayed	36.363	.132	36.098	36.629
50NP	39.081	.132	38.816	39.346
150NP	39.792	.132	39.526	40.057
300NP	41.006	.132	40.741	41.271
600NP	42.462	.132	42.196	42.727

4. FC_Levels * NPs

Dependent Variable: EC_Leakage

FC_Levels	NPs	Mean	Std. Error	95% Confidence Interval	
				Lower Bound	Upper Bound
FFC	Cont	34.767	.264	34.236	35.297
	Water sprayed	33.973	.264	33.443	34.504
	50NP	36.083	.264	35.553	36.614
	150NP	35.897	.264	35.366	36.427
	300NP	37.273	.264	36.743	37.804
	600NP	38.617	.264	38.086	39.147
75%FC	Cont	35.680	.264	35.149	36.211
	Water sprayed	34.370	.264	33.839	34.901
	50NP	36.783	.264	36.253	37.314
	150NP	36.553	.264	36.023	37.084
	300NP	38.320	.264	37.789	38.851
	600NP	40.077	.264	39.546	40.607
50%FC	Cont	36.887	.264	36.356	37.417
	Water sprayed	36.650	.264	36.119	37.181
	50NP	38.247	.264	37.716	38.777
	150NP	39.140	.264	38.609	39.671
	300NP	40.900	.264	40.369	41.431
	600NP	42.787	.264	42.256	43.317
25%FC	Cont	41.497	.264	40.966	42.027
	Water sprayed	40.460	.264	39.929	40.991
	50NP	45.210	.264	44.679	45.741
	150NP	47.577	.264	47.046	48.107
	300NP	47.530	.264	46.999	48.061
	600NP	48.367	.264	47.836	48.897

Post Hoc Tests

FC_Levels

Homogeneous Subsets

EC_Leakage

Duncan[a,b]

FC_Levels	N	Subset			
		1	2	3	4
FFC	18	36.1017			
75%FC	18		36.9639		
50%FC	18			39.1017	
25%FC	18				45.1067
Sig.		1.000	1.000	1.000	1.000

Means for groups in homogeneous subsets are displayed.
Based on observed means.
The error term is Mean Square(Error) = .209.

a. Uses Harmonic Mean Sample Size = 18.000.

b. Alpha = .05.

NPs

Homogeneous Subsets

EC_Leakage

Duncan[a,b]

NPs	N	Subset					
		1	2	3	4	5	6
Water sprayed	12	36.3633					
Cont	12		37.2075				
50NP	12			39.0808			
150NP	12				39.7917		
300NP	12					41.0058	
600NP	12						42.4617
Sig.		1.000	1.000	1.000	1.000	1.000	1.000

Means for groups in homogeneous subsets are displayed.
Based on observed means.
The error term is Mean Square(Error) = .209.

a. Uses Harmonic Mean Sample Size = 12.000.

b. Alpha = .05.

Profile Plots

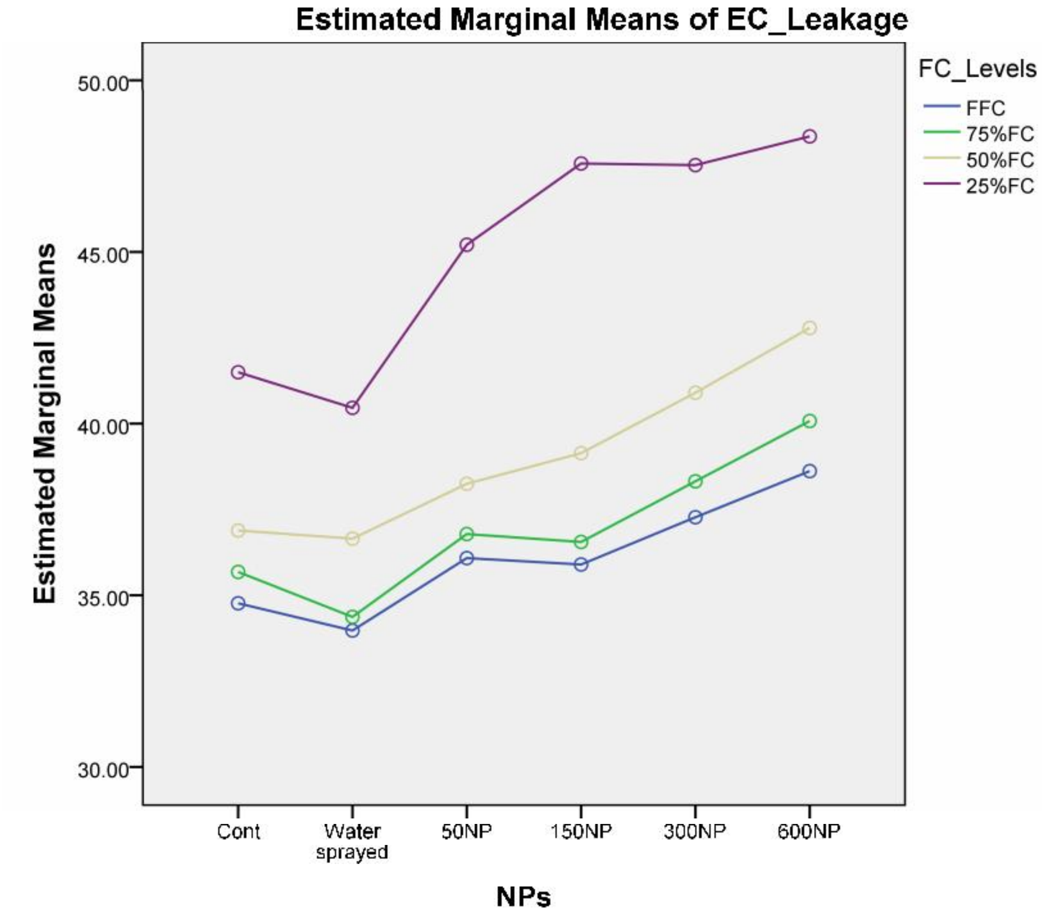


```
UNIANOVA EC_Leakage BY FC_LevOP OPs
  /METHOD=SSTYPE(3)
  /INTERCEPT=EXCLUDE
  /SAVE=SEPRED
  /POSTHOC=FC_LevOP OPs(DUNCAN)
  /PLOT=PROFILE(OPs*FC_LevOP)
  /EMMEANS=TABLES(OVERALL)
  /EMMEANS=TABLES(FC_LevOP)
  /EMMEANS=TABLES(OPs)
  /EMMEANS=TABLES(FC_LevOP*OPs)
  /PRINT=DESCRIPTIVE
  /CRITERIA=ALPHA(.05)
  /DESIGN=FC_LevOP OPs FC_LevOP*OPs.
```

Univariate Analysis of Variance

[DataSet1] E:\Amna M Sc\Paper\Two Way ANOVA H.sav

Between-Subjects Factors

		Value Label	N
FC_LevOP	1.00	FFC	18
	2.00	75%FC	18
	3.00	50%FC	18
	4.00	25%FC	18
OPs	1.00	Cont	12
	2.00	Water sprayed	12
	3.00	50OPs	12
	4.00	150OPs	12
	5.00	300OPs	12
	6.00	600OPs	12

Descriptive Statistics

Dependent Variable: EC_Leakage

FC_LevOP	OPs	Mean	Std. Deviation	N
FFC	Cont	34.7667	.00577	3
	Water sprayed	33.9733	.23965	3
	50OPs	36.4600	.26058	3
	150OPs	36.1267	.67575	3
	300OPs	37.1433	.28290	3
	600OPs	38.2200	.30348	3
	Total	36.1150	1.48612	18
75%FC	Cont	35.6800	.05568	3
	Water sprayed	34.3700	.03606	3
	50OPs	37.0767	.87363	3
	150OPs	37.5167	.89512	3
	300OPs	38.9933	1.00481	3
	600OPs	39.3033	.95091	3
	Total	37.1567	1.89669	18
50%FC	Cont	36.8867	.59341	3
	Water sprayed	36.6500	.12530	3
	50OPs	38.3100	.26514	3
	150OPs	38.7700	.16823	3
	300OPs	40.8933	1.03016	3
	600OPs	41.3100	.50863	3
	Total	38.8033	1.89724	18
25%FC	Cont	41.4967	.69974	3
	Water sprayed	40.4600	.72021	3
	50OPs	42.1833	.10970	3
	150OPs	42.6567	.89646	3
	300OPs	43.4467	1.04409	3
	600OPs	45.8333	.42004	3
	Total	42.6794	1.83916	18

Descriptive Statistics

Dependent Variable: EC_Leakage

FC_LevOP	OPs	Mean	Std. Deviation	N
Total	Cont	37.2075	2.73134	12
	Water sprayed	36.3633	2.71087	12
	50OPs	38.5075	2.35869	12
	150OPs	38.7675	2.61416	12
	300OPs	40.1192	2.55603	12
	600OPs	41.1667	3.08549	12
	Total	38.6886	3.06222	72

Tests of Between-Subjects Effects

Dependent Variable: EC_Leakage

Source	Type III Sum of Squares	df	Mean Square	F	Sig.
Model	108417.774[a]	24	4517.407	11896.001	.000
FC_LevOP	448.384	3	149.461	393.587	.000
OPs	189.923	5	37.985	100.027	.000
FC_LevOP * OPs	9.246	15	.616	1.623	.103
Error	18.228	48	.380		
Total	108436.002	72			

a. R Squared = 1.000 (Adjusted R Squared = 1.000)

Estimated Marginal Means

1. Grand Mean

Dependent Variable: EC_Leakage

Mean	Std. Error	95% Confidence Interval	
		Lower Bound	Upper Bound
38.689	.073	38.543	38.835

2. FC_LevOP

Dependent Variable: EC_Leakage

FC_LevOP	Mean	Std. Error	95% Confidence Interval	
			Lower Bound	Upper Bound
FFC	36.115	.145	35.823	36.407
75%FC	37.157	.145	36.865	37.449
50%FC	38.803	.145	38.511	39.095
25%FC	42.679	.145	42.387	42.971

3. OPs

Dependent Variable: EC_Leakage

OPs	Mean	Std. Error	95% Confidence Interval	
			Lower Bound	Upper Bound
Cont	37.208	.178	36.850	37.565
Water sprayed	36.363	.178	36.006	36.721
50OPs	38.508	.178	38.150	38.865
150OPs	38.768	.178	38.410	39.125
300OPs	40.119	.178	39.761	40.477
600OPs	41.167	.178	40.809	41.524

4. FC_LevOP * OPs

Dependent Variable: EC_Leakage

FC_LevOP	OPs	Mean	Std. Error	95% Confidence Interval	
				Lower Bound	Upper Bound
FFC	Cont	34.767	.356	34.051	35.482
	Water sprayed	33.973	.356	33.258	34.689
	50OPs	36.460	.356	35.745	37.175
	150OPs	36.127	.356	35.411	36.842
	300OPs	37.143	.356	36.428	37.859
	600OPs	38.220	.356	37.505	38.935
75%FC	Cont	35.680	.356	34.965	36.395
	Water sprayed	34.370	.356	33.655	35.085
	50OPs	37.077	.356	36.361	37.792
	150OPs	37.517	.356	36.801	38.232
	300OPs	38.993	.356	38.278	39.709
	600OPs	39.303	.356	38.588	40.019
50%FC	Cont	36.887	.356	36.171	37.602
	Water sprayed	36.650	.356	35.935	37.365
	50OPs	38.310	.356	37.595	39.025
	150OPs	38.770	.356	38.055	39.485
	300OPs	40.893	.356	40.178	41.609
	600OPs	41.310	.356	40.595	42.025
25%FC	Cont	41.497	.356	40.781	42.212
	Water sprayed	40.460	.356	39.745	41.175
	50OPs	42.183	.356	41.468	42.899
	150OPs	42.657	.356	41.941	43.372
	300OPs	43.447	.356	42.731	44.162
	600OPs	45.833	.356	45.118	46.549

Post Hoc Tests

FC_LevOP

Homogeneous Subsets

EC_Leakage

Duncan[a,b]

FC_LevOP	N	Subset			
		1	2	3	4
FFC	18	36.1150			
75%FC	18		37.1567		
50%FC	18			38.8033	
25%FC	18				42.6794
Sig.		1.000	1.000	1.000	1.000

Means for groups in homogeneous subsets are displayed.
Based on observed means.
The error term is Mean Square(Error) = .380.

a. Uses Harmonic Mean Sample Size = 18.000.

b. Alpha = .05.

OPs

Homogeneous Subsets

EC_Leakage

Duncan[a,b]

OPs	N	Subset				
		1	2	3	4	5
Water sprayed	12	36.3633				
Cont	12		37.2075			
50OPs	12			38.5075		
150OPs	12			38.7675		
300OPs	12				40.1192	
600OPs	12					41.1667
Sig.		1.000	1.000	.307	1.000	1.000

Means for groups in homogeneous subsets are displayed.
Based on observed means.
The error term is Mean Square(Error) = .380.

a. Uses Harmonic Mean Sample Size = 12.000.

b. Alpha = .05.

Profile Plots

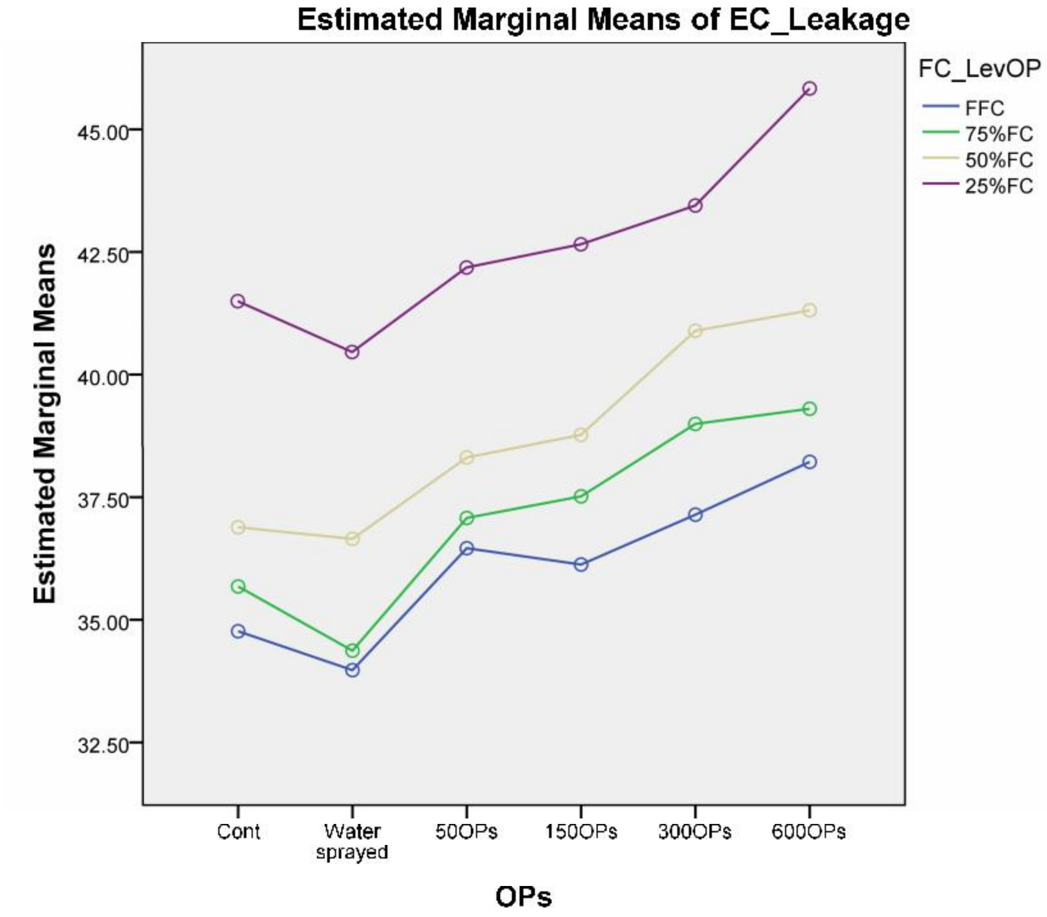


```
UNIANOVA Mem_Inj BY FC_Levels NPs
  /METHOD=SSTYPE(3)
  /INTERCEPT=EXCLUDE
  /SAVE=SEPRED
  /POSTHOC=FC_Levels NPs(DUNCAN)
  /PLOT=PROFILE(NPs*FC_Levels)
  /EMMEANS=TABLES(OVERALL)
  /EMMEANS=TABLES(FC_Levels)
  /EMMEANS=TABLES(NPs)
  /EMMEANS=TABLES(FC_Levels*NPs)
  /PRINT=DESCRIPTIVE
  /CRITERIA=ALPHA(.05)
  /DESIGN=FC_Levels NPs FC_Levels*NPs.
```

Univariate Analysis of Variance

```
[DataSet1] E:\Amna M Sc\Paper\Two Way ANOVA H.sav
```

Between-Subjects Factors

		Value Label	N
FC_Levels	1	FFC	18
	2	75%FC	18
	3	50%FC	18
	4	25%FC	18
NPs	1	Cont	12
	2	Water sprayed	12
	3	50NP	12
	4	150NP	12
	5	300NP	12
	6	600NP	12

Descriptive Statistics

Dependent Variable: Mem_Inj

FC_Levels	NPs	Mean	Std. Deviation	N
FFC	Cont	.0020	.00200	3
	Water sprayed	-1.0895-	.32516	3
	50NP	2.0150	.07398	3
	150NP	1.7290	.01562	3
	300NP	3.8433	.37192	3
	600NP	5.9037	.59504	3
	Total	2.0672	2.39937	18
75%FC	Cont	1.3977	.08523	3
	Water sprayed	-.6137-	.05522	3
	50NP	3.0867	.35548	3
	150NP	2.7370	.75462	3
	300NP	5.4447	.75779	3
	600NP	8.1347	.33655	3
	Total	3.3645	2.91575	18
50%FC	Cont	3.2453	.90674	3
	Water sprayed	2.8890	.18884	3
	50NP	5.3330	.25237	3
	150NP	6.7033	.90079	3
	300NP	9.4017	.99272	3
	600NP	12.2910	1.08323	3
	Total	6.6439	3.50112	18
25%FC	Cont	10.3103	1.06984	3
	Water sprayed	8.7203	1.10397	3
	50NP	16.0070	.84879	3
	150NP	19.6357	1.38253	3
	300NP	19.5663	.18847	3
	600NP	20.8493	.89209	3
	Total	15.8482	4.94496	18

Descriptive Statistics

Dependent Variable: Mem_Inj

FC_Levels	NPs	Mean	Std. Deviation	N
Total	Cont	3.7388	4.18403	12
	Water sprayed	2.4765	4.12300	12
	50NP	6.6104	5.81705	12
	150NP	7.7013	7.49423	12
	300NP	9.5640	6.41568	12
	600NP	11.7947	5.99903	12
	Total	6.9810	6.45006	72

Tests of Between-Subjects Effects

Dependent Variable: Mem_Inj

Source	Type III Sum of Squares	df	Mean Square	F	Sig.
Model	6439.224[a]	24	268.301	549.618	.000
FC_Levels	2087.358	3	695.786	1425.326	.000
NPs	735.615	5	147.123	301.383	.000
FC_Levels * NPs	107.427	15	7.162	14.671	.000
Error	23.432	48	.488		
Total	6462.656	72			

a. R Squared = .996 (Adjusted R Squared = .995)

Estimated Marginal Means

1. Grand Mean

Dependent Variable: Mem_Inj

Mean	Std. Error	95% Confidence Interval	
		Lower Bound	Upper Bound
6.981	.082	6.815	7.147

2. FC_Levels

Dependent Variable: Mem_Inj

FC_Levels	Mean	Std. Error	95% Confidence Interval	
			Lower Bound	Upper Bound
FFC	2.067	.165	1.736	2.398
75%FC	3.365	.165	3.033	3.696
50%FC	6.644	.165	6.313	6.975
25%FC	15.848	.165	15.517	16.179

3. NPs

Dependent Variable: Mem_Inj

NPs	Mean	Std. Error	95% Confidence Interval	
			Lower Bound	Upper Bound
Cont	3.739	.202	3.333	4.144
Water sprayed	2.477	.202	2.071	2.882
50NP	6.610	.202	6.205	7.016
150NP	7.701	.202	7.296	8.107
300NP	9.564	.202	9.158	9.970
600NP	11.795	.202	11.389	12.200

4. FC_Levels * NPs

Dependent Variable: Mem_Inj

FC_Levels	NPs	Mean	Std. Error	95% Confidence Interval	
				Lower Bound	Upper Bound
FFC	Cont	.002	.403	-.809-	.813
	Water sprayed	-1.090-	.403	-1.901-	-.278-
	50NP	2.015	.403	1.204	2.826
	150NP	1.729	.403	.918	2.540
	300NP	3.843	.403	3.032	4.654
	600NP	5.904	.403	5.093	6.715
75%FC	Cont	1.398	.403	.587	2.209
	Water sprayed	-.614-	.403	-1.425-	.197
	50NP	3.087	.403	2.276	3.898
	150NP	2.737	.403	1.926	3.548
	300NP	5.445	.403	4.634	6.256
	600NP	8.135	.403	7.324	8.946
50%FC	Cont	3.245	.403	2.434	4.056
	Water sprayed	2.889	.403	2.078	3.700
	50NP	5.333	.403	4.522	6.144
	150NP	6.703	.403	5.892	7.514
	300NP	9.402	.403	8.591	10.213
	600NP	12.291	.403	11.480	13.102
25%FC	Cont	10.310	.403	9.499	11.121
	Water sprayed	8.720	.403	7.909	9.531
	50NP	16.007	.403	15.196	16.818
	150NP	19.636	.403	18.825	20.447
	300NP	19.566	.403	18.755	20.377
	600NP	20.849	.403	20.038	21.660

Post Hoc Tests

FC_Levels

Homogeneous Subsets

Mem_Inj

Duncan[a,b]

FC_Levels	N	Subset			
		1	2	3	4
FFC	18	2.0672			
75%FC	18		3.3645		
50%FC	18			6.6439	
25%FC	18				15.8482
Sig.		1.000	1.000	1.000	1.000

Means for groups in homogeneous subsets are displayed.
Based on observed means.
The error term is Mean Square(Error) = .488.

a. Uses Harmonic Mean Sample Size = 18.000.

b. Alpha = .05.

NPs

Homogeneous Subsets

Mem_Inj

Duncan[a,b]

NPs	N	Subset					
		1	2	3	4	5	6
Water sprayed	12	2.4765					
Cont	12		3.7388				
50NP	12			6.6104			
150NP	12				7.7013		
300NP	12					9.5640	
600NP	12						11.7947
Sig.		1.000	1.000	1.000	1.000	1.000	1.000

Means for groups in homogeneous subsets are displayed.
Based on observed means.
The error term is Mean Square(Error) = .488.

a. Uses Harmonic Mean Sample Size = 12.000.

b. Alpha = .05.

Profile Plots

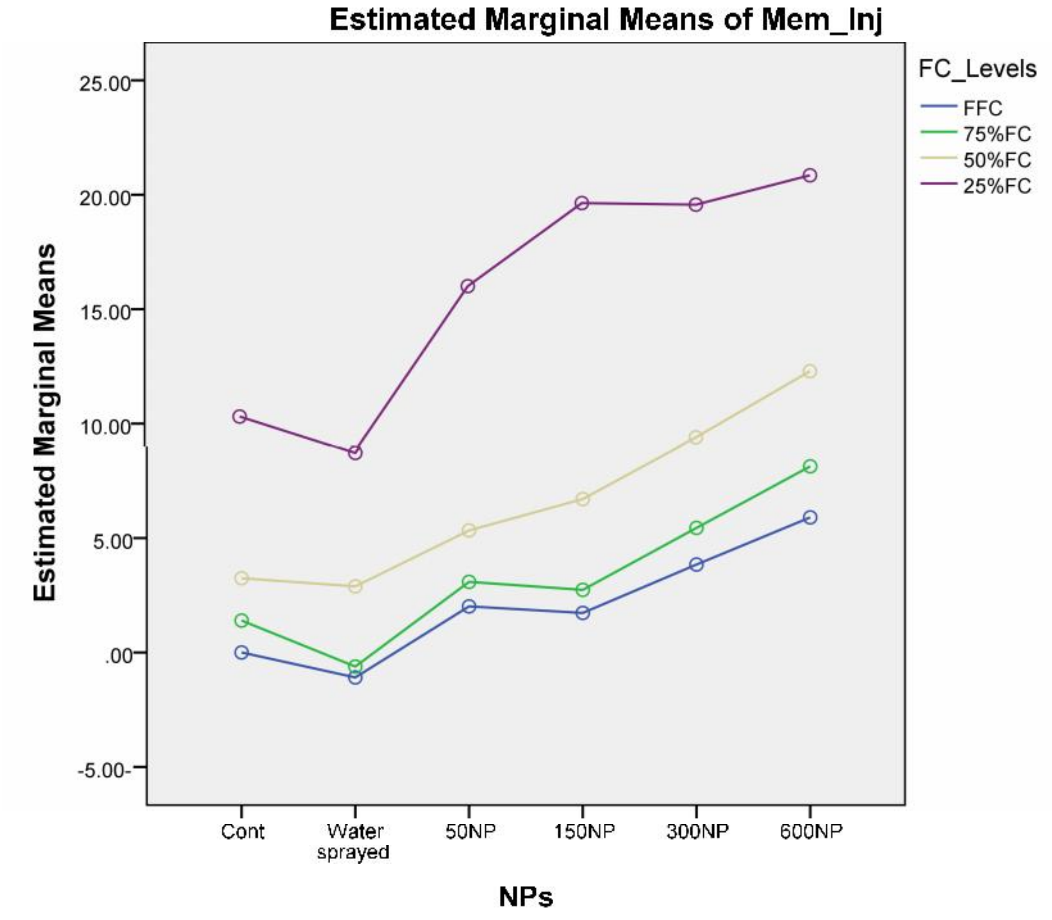


```
UNIANOVA Mem_Inj BY FC_LevOP OPs
  /METHOD=SSTYPE(3)
  /INTERCEPT=EXCLUDE
  /SAVE=SEPRED
  /POSTHOC=FC_LevOP OPs(DUNCAN)
  /PLOT=PROFILE(OPs*FC_LevOP)
  /EMMEANS=TABLES(OVERALL)
  /PRINT=DESCRIPTIVE
  /CRITERIA=ALPHA(.05)
  /DESIGN=FC_LevOP OPs FC_LevOP*OPs.
```

Univariate Analysis of Variance

```
[DataSet1] E:\Amna M Sc\Paper\Two Way ANOVA H.sav
```

Between-Subjects Factors

		Value Label	N
FC_LevOP	1.00	FFC	18
	2.00	75%FC	18
	3.00	50%FC	18
	4.00	25%FC	18
OPs	1.00	Cont	12
	2.00	Water sprayed	12
	3.00	50OPs	12
	4.00	150OPs	12
	5.00	300OPs	12
	6.00	600OPs	12

Descriptive Statistics

Dependent Variable: Mem_Inj

FC_LevOP	OPs	Mean	Std. Deviation	N
FFC	Cont	.0020	.00200	3
	Water sprayed	-1.0895-	.32516	3
	50OPs	2.5943	.40511	3
	150OPs	2.0780	1.03515	3
	300OPs	3.6423	.42795	3
	600OPs	5.2897	.46806	3
	Total	2.0861	2.24396	18
75%FC	Cont	1.3977	.08523	3
	Water sprayed	-.6137-	.05522	3
	50OPs	3.5377	1.33919	3
	150OPs	4.2150	1.37847	3
	300OPs	6.4773	1.54212	3
	600OPs	6.9563	1.45689	3
	Total	3.6617	2.90977	18
50%FC	Cont	3.2453	.90674	3
	Water sprayed	2.8890	.18884	3
	50OPs	5.4280	.40730	3
	150OPs	6.1340	.25363	3
	300OPs	9.3877	1.57876	3
	600OPs	10.0287	.77631	3
	Total	6.1854	2.90741	18
25%FC	Cont	10.3103	1.06984	3
	Water sprayed	8.7203	1.10397	3
	50OPs	11.3717	.17090	3
	150OPs	12.0917	1.36902	3
	300OPs	13.3023	1.59740	3
	600OPs	16.9623	.64445	3
	Total	12.1264	2.81994	18

Descriptive Statistics

Dependent Variable: Mem_Inj

FC_LevOP	OPs	Mean	Std. Deviation	N
Total	Cont	3.7388	4.18403	12
	Water sprayed	2.4765	4.12300	12
	50OPs	5.7329	3.61789	12
	150OPs	6.1297	4.00798	12
	300OPs	8.2024	3.91691	12
	600OPs	9.8093	4.72960	12
	Total	6.0149	4.68557	72

Tests of Between-Subjects Effects

Dependent Variable: Mem_Inj

Source	Type III Sum of Squares	df	Mean Square	F	Sig.
Model	4120.962[a]	24	171.707	192.883	.000
FC_LevOP	1050.349	3	350.116	393.295	.000
OPs	443.706	5	88.741	99.686	.000
FC_LevOP * OPs	21.986	15	1.466	1.646	.096
Error	42.730	48	.890		
Total	4163.692	72			

a. R Squared = .990 (Adjusted R Squared = .985)

Estimated Marginal Means

Grand Mean

Dependent Variable: Mem_Inj

Mean	Std. Error	95% Confidence Interval	
		Lower Bound	Upper Bound
6.015	.111	5.791	6.239

Post Hoc Tests

FC_LevOP

Homogeneous Subsets

Mem_Inj

Duncan[a,b]

FC_LevOP	N	Subset			
		1	2	3	4
FFC	18	2.0861			
75%FC	18		3.6617		
50%FC	18			6.1854	
25%FC	18				12.1264
Sig.		1.000	1.000	1.000	1.000

Means for groups in homogeneous subsets are displayed.
Based on observed means.
The error term is Mean Square(Error) = .890.

a. Uses Harmonic Mean Sample Size = 18.000.

b. Alpha = .05.

OPs

Homogeneous Subsets

Mem_Inj

Duncan[a,b]

OPs	N	Subset				
		1	2	3	4	5
Water sprayed	12	2.4765				
Cont	12		3.7388			
50OPs	12			5.7329		
150OPs	12			6.1297		
300OPs	12				8.2024	
600OPs	12					9.8093
Sig.		1.000	1.000	.308	1.000	1.000

Means for groups in homogeneous subsets are displayed.
Based on observed means.
The error term is Mean Square(Error) = .890.

a. Uses Harmonic Mean Sample Size = 12.000.

b. Alpha = .05.

Profile Plots

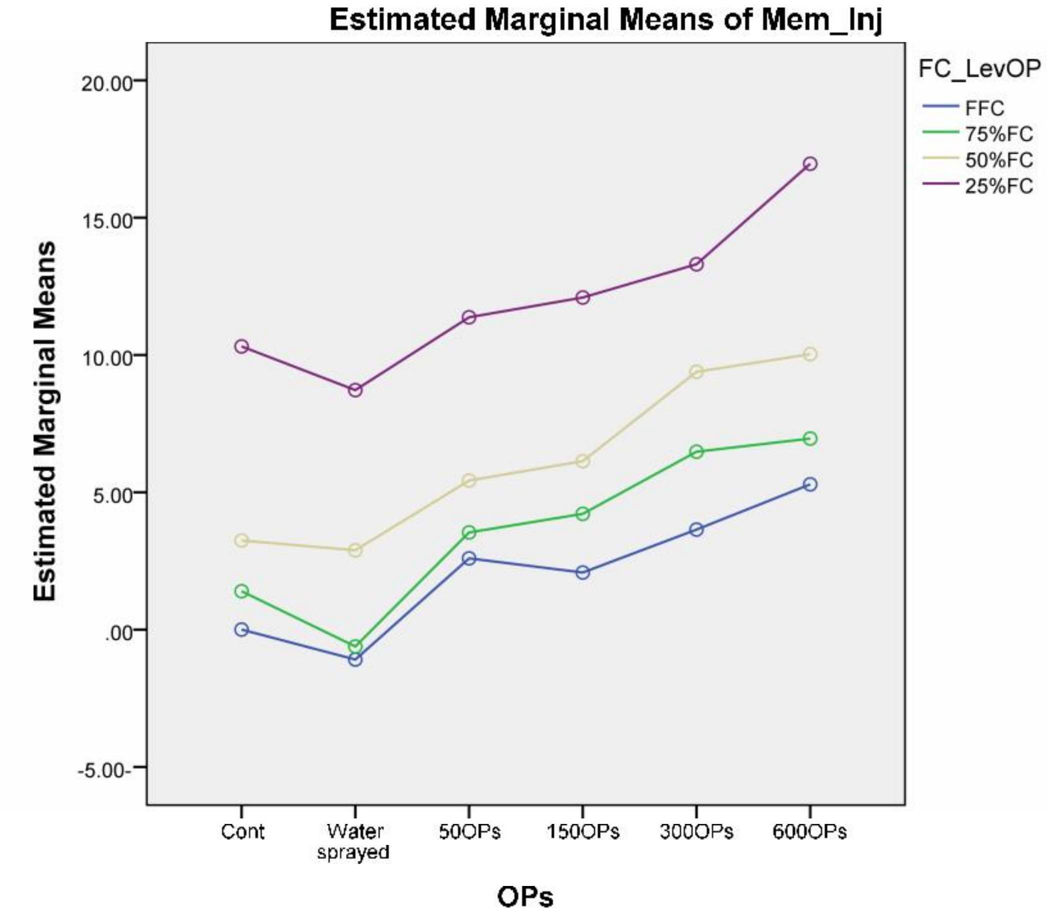


```
UNIANOVA H2O2_Sh BY FC_Levels NPs
  /METHOD=SSTYPE(3)
  /INTERCEPT=EXCLUDE
  /SAVE=SEPRED
  /POSTHOC=FC_Levels NPs(DUNCAN)
  /PLOT=PROFILE(NPs*FC_Levels)
  /EMMEANS=TABLES(OVERALL)
  /EMMEANS=TABLES(FC_Levels)
  /EMMEANS=TABLES(NPs)
  /EMMEANS=TABLES(FC_Levels*NPs)
  /PRINT=DESCRIPTIVE
  /CRITERIA=ALPHA(.05)
  /DESIGN=FC_Levels NPs FC_Levels*NPs.
```

Univariate Analysis of Variance

[DataSet1] E:\Amna M Sc\Paper\Two Way ANOVA H.sav

Between-Subjects Factors

		Value Label	N
FC_Levels	1	FFC	18
	2	75%FC	18
	3	50%FC	18
	4	25%FC	18
NPs	1	Cont	12
	2	Water sprayed	12
	3	50NP	12
	4	150NP	12
	5	300NP	12
	6	600NP	12

Descriptive Statistics

Dependent Variable: H2O2_Sh

FC_Levels	NPs	Mean	Std. Deviation	N
FFC	Cont	7.4833	.72473	3
	Water sprayed	7.8533	.92154	3
	50NP	9.7467	2.53319	3
	150NP	10.3033	.62740	3
	300NP	12.0267	3.59634	3
	600NP	18.5267	1.27210	3
	Total	10.9900	4.14133	18
75%FC	Cont	8.8067	3.48621	3
	Water sprayed	8.6300	.93290	3
	50NP	14.4767	.72954	3
	150NP	17.6233	2.57078	3
	300NP	17.3533	2.20509	3
	600NP	18.7500	1.88072	3
	Total	14.2733	4.63171	18
50%FC	Cont	11.1067	1.89210	3
	Water sprayed	12.4533	1.15932	3
	50NP	16.2033	1.54507	3
	150NP	20.7900	2.69902	3
	300NP	20.2767	2.17480	3
	600NP	22.6633	2.41299	3
	Total	17.2489	4.78243	18
25%FC	Cont	14.1500	3.06379	3
	Water sprayed	17.0167	1.45260	3
	50NP	19.2500	.34828	3
	150NP	24.7333	1.59954	3
	300NP	27.7200	.86603	3
	600NP	25.6933	4.01559	3
	Total	21.4272	5.42187	18

Descriptive Statistics

Dependent Variable: H2O2_Sh

FC_Levels	NPs	Mean	Std. Deviation	N
Total	Cont	10.3867	3.41270	12
	Water sprayed	11.4883	3.91939	12
	50NP	14.9192	3.82555	12
	150NP	18.3625	5.79672	12
	300NP	19.3442	6.26790	12
	600NP	21.4083	3.81536	12
	Total	15.9849	6.06134	72

Tests of Between-Subjects Effects

Dependent Variable: H2O2_Sh

Source	Type III Sum of Squares	df	Mean Square	F	Sig.
Model	20790.663[a]	24	866.278	193.399	.000
FC_Levels	1063.710	3	354.570	79.159	.000
NPs	1188.557	5	237.711	53.070	.000
FC_Levels * NPs	141.260	15	9.417	2.102	.026
Error	215.003	48	4.479		
Total	21005.667	72			

a. R Squared = .990 (Adjusted R Squared = .985)

Estimated Marginal Means

1. Grand Mean

Dependent Variable: H2O2_Sh

Mean	Std. Error	95% Confidence Interval	
		Lower Bound	Upper Bound
15.985	.249	15.483	16.486

2. FC_Levels

Dependent Variable: H2O2_Sh

FC_Levels	Mean	Std. Error	95% Confidence Interval	
			Lower Bound	Upper Bound
FFC	10.990	.499	9.987	11.993
75%FC	14.273	.499	13.270	15.276
50%FC	17.249	.499	16.246	18.252
25%FC	21.427	.499	20.424	22.430

3. NPs

Dependent Variable: H2O2_Sh

NPs	Mean	Std. Error	95% Confidence Interval	
			Lower Bound	Upper Bound
Cont	10.387	.611	9.158	11.615
Water sprayed	11.488	.611	10.260	12.717
50NP	14.919	.611	13.691	16.148
150NP	18.363	.611	17.134	19.591
300NP	19.344	.611	18.116	20.573
600NP	21.408	.611	20.180	22.637

4. FC_Levels * NPs

Dependent Variable: H2O2_Sh

FC_Levels	NPs	Mean	Std. Error	95% Confidence Interval	
				Lower Bound	Upper Bound
FFC	Cont	7.483	1.222	5.027	9.940
	Water sprayed	7.853	1.222	5.397	10.310
	50NP	9.747	1.222	7.290	12.203
	150NP	10.303	1.222	7.847	12.760
	300NP	12.027	1.222	9.570	14.483
	600NP	18.527	1.222	16.070	20.983
75%FC	Cont	8.807	1.222	6.350	11.263
	Water sprayed	8.630	1.222	6.173	11.087
	50NP	14.477	1.222	12.020	16.933
	150NP	17.623	1.222	15.167	20.080
	300NP	17.353	1.222	14.897	19.810
	600NP	18.750	1.222	16.293	21.207
50%FC	Cont	11.107	1.222	8.650	13.563
	Water sprayed	12.453	1.222	9.997	14.910
	50NP	16.203	1.222	13.747	18.660
	150NP	20.790	1.222	18.333	23.247
	300NP	20.277	1.222	17.820	22.733
	600NP	22.663	1.222	20.207	25.120
25%FC	Cont	14.150	1.222	11.693	16.607
	Water sprayed	17.017	1.222	14.560	19.473
	50NP	19.250	1.222	16.793	21.707
	150NP	24.733	1.222	22.277	27.190
	300NP	27.720	1.222	25.263	30.177
	600NP	25.693	1.222	23.237	28.150

Post Hoc Tests

FC_Levels

Homogeneous Subsets

H2O2_Sh

Duncan[a,b]

FC_Levels	N	Subset 1	2	3	4
FFC	18	10.9900			
75%FC	18		14.2733		
50%FC	18			17.2489	
25%FC	18				21.4272
Sig.		1.000	1.000	1.000	1.000

Means for groups in homogeneous subsets are displayed.
Based on observed means.
The error term is Mean Square(Error) = 4.479.

a. Uses Harmonic Mean Sample Size = 18.000.

b. Alpha = .05.

NPs

Homogeneous Subsets

H2O2_Sh

Duncan[a,b]

NPs	N	Subset 1	2	3	4
Cont	12	10.3867			
Water sprayed	12	11.4883			
50NP	12		14.9192		
150NP	12			18.3625	
300NP	12			19.3442	
600NP	12				21.4083
Sig.		.208	1.000	.262	1.000

Means for groups in homogeneous subsets are displayed.
Based on observed means.
The error term is Mean Square(Error) = 4.479.

a. Uses Harmonic Mean Sample Size = 12.000.

b. Alpha = .05.

Profile Plots

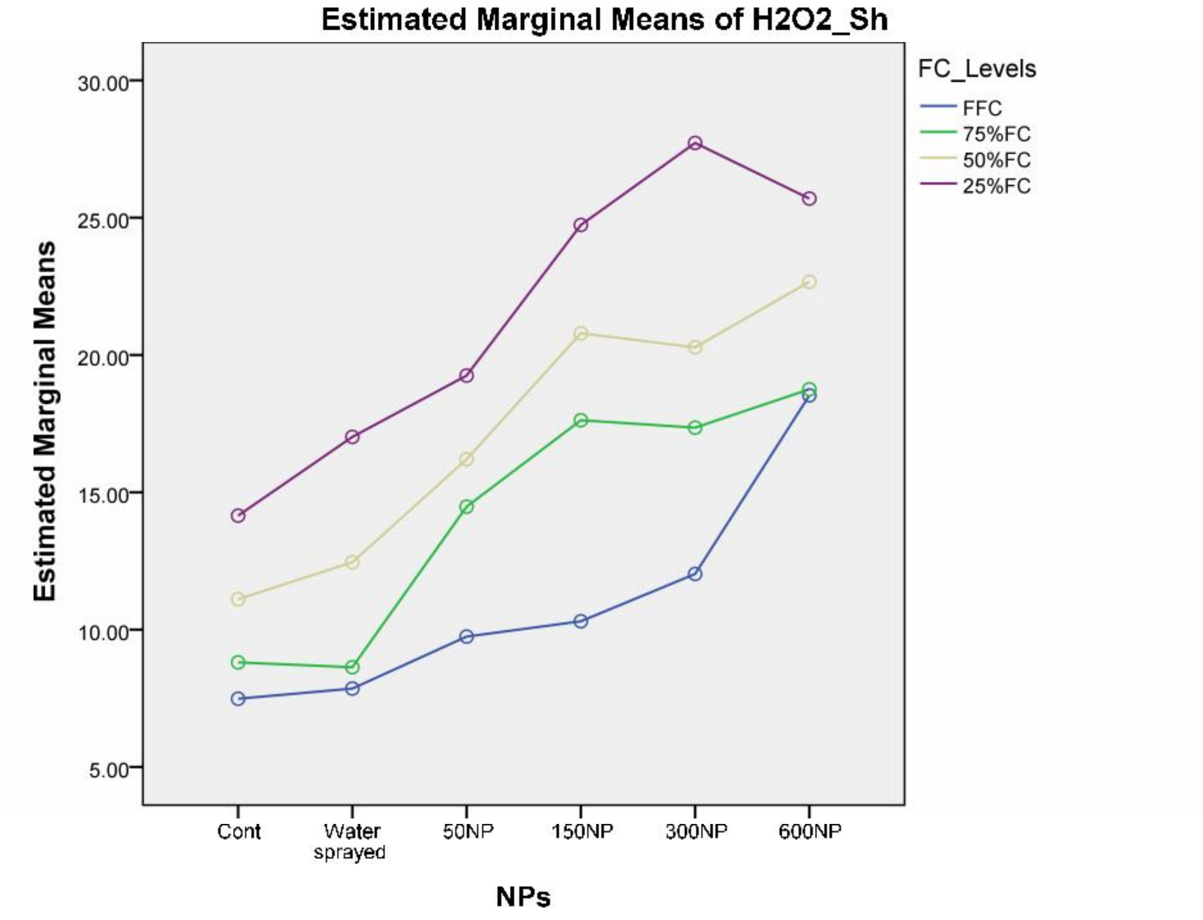


```
UNIANOVA H2O2_Sh BY FC_LevOP OPs
  /METHOD=SSTYPE(3)
  /INTERCEPT=EXCLUDE
  /SAVE=SEPRED
  /POSTHOC=FC_LevOP OPs(DUNCAN)
  /PLOT=PROFILE(OPs*FC_LevOP)
  /EMMEANS=TABLES(OVERALL)
  /EMMEANS=TABLES(FC_LevOP)
  /EMMEANS=TABLES(OPs)
  /EMMEANS=TABLES(FC_LevOP*OPs)
  /PRINT=DESCRIPTIVE
  /CRITERIA=ALPHA(.05)
  /DESIGN=FC_LevOP OPs FC_LevOP*OPs.
```

Univariate Analysis of Variance

```
[DataSet1] E:\Amna M Sc\Paper\Two Way ANOVA H.sav
```

Between-Subjects Factors

		Value Label	N
FC_LevOP	1.00	FFC	18
	2.00	75%FC	18
	3.00	50%FC	18
	4.00	25%FC	18
OPs	1.00	Cont	12
	2.00	Water sprayed	12
	3.00	50OPs	12
	4.00	150OPs	12
	5.00	300OPs	12
	6.00	600OPs	12

Descriptive Statistics

Dependent Variable: H2O2_Sh

FC_LevOP	OPs	Mean	Std. Deviation	N
FFC	Cont	7.4833	.72473	3
	Water sprayed	7.8533	.92154	3
	50OPs	17.0933	3.00776	3
	150OPs	18.0667	.53929	3
	300OPs	18.5967	2.75780	3
	600OPs	19.9700	.61555	3
	Total	14.8439	5.49839	18
75%FC	Cont	8.8067	3.48621	3
	Water sprayed	8.6300	.93290	3
	50OPs	18.9700	.12490	3
	150OPs	18.8167	1.49768	3
	300OPs	25.7867	3.29023	3
	600OPs	30.0267	3.07168	3
	Total	18.5061	8.41589	18
50%FC	Cont	11.1067	1.89210	3
	Water sprayed	12.4533	1.15932	3
	50OPs	19.4933	1.03404	3
	150OPs	20.5800	.82146	3
	300OPs	32.5400	2.35204	3
	600OPs	34.9967	1.10528	3
	Total	21.8617	9.45834	18
25%FC	Cont	14.1500	3.06379	3
	Water sprayed	17.0167	1.45260	3
	50OPs	23.6333	4.22073	3
	150OPs	30.0867	3.28982	3
	300OPs	37.4733	3.21351	3
	600OPs	38.4000	2.37968	3
	Total	26.7933	9.96283	18

Descriptive Statistics

Dependent Variable: H2O2_Sh

FC_LevOP	OPs	Mean	Std. Deviation	N
Total	Cont	10.3867	3.41270	12
	Water sprayed	11.4883	3.91939	12
	50OPs	19.7975	3.36158	12
	150OPs	21.8875	5.28254	12
	300OPs	28.5992	7.83525	12
	600OPs	30.8483	7.46596	12
	Total	20.5013	9.43466	72

Tests of Between-Subjects Effects

Dependent Variable: H2O2_Sh

Source	Type III Sum of Squares	df	Mean Square	F	Sig.
Model	36334.032[a]	24	1513.918	293.524	.000
FC_LevOP	1393.692	3	464.564	90.072	.000
OPs	4303.114	5	860.623	166.861	.000
FC_LevOP * OPs	375.536	15	25.036	4.854	.000
Error	247.571	48	5.158		
Total	36581.603	72			

a. R Squared = .993 (Adjusted R Squared = .990)

Estimated Marginal Means

1. Grand Mean

Dependent Variable: H2O2_Sh

Mean	Std. Error	95% Confidence Interval	
		Lower Bound	Upper Bound
20.501	.268	19.963	21.039

2. FC_LevOP

Dependent Variable: H2O2_Sh

FC_LevOP	Mean	Std. Error	95% Confidence Interval	
			Lower Bound	Upper Bound
FFC	14.844	.535	13.768	15.920
75%FC	18.506	.535	17.430	19.582
50%FC	21.862	.535	20.785	22.938
25%FC	26.793	.535	25.717	27.870

3. OPs

Dependent Variable: H2O2_Sh

OPs	Mean	Std. Error	95% Confidence Interval	
			Lower Bound	Upper Bound
Cont	10.387	.656	9.068	11.705
Water sprayed	11.488	.656	10.170	12.807
50OPs	19.798	.656	18.479	21.116
150OPs	21.888	.656	20.569	23.206
300OPs	28.599	.656	27.281	29.917
600OPs	30.848	.656	29.530	32.167

4. FC_LevOP * OPs

Dependent Variable: H2O2_Sh

FC_LevOP	OPs	Mean	Std. Error	95% Confidence Interval	
				Lower Bound	Upper Bound
FFC	Cont	7.483	1.311	4.847	10.120
	Water sprayed	7.853	1.311	5.217	10.490
	50OPs	17.093	1.311	14.457	19.730
	150OPs	18.067	1.311	15.430	20.703
	300OPs	18.597	1.311	15.960	21.233
	600OPs	19.970	1.311	17.334	22.606
75%FC	Cont	8.807	1.311	6.170	11.443
	Water sprayed	8.630	1.311	5.994	11.266
	50OPs	18.970	1.311	16.334	21.606
	150OPs	18.817	1.311	16.180	21.453
	300OPs	25.787	1.311	23.150	28.423
	600OPs	30.027	1.311	27.390	32.663
50%FC	Cont	11.107	1.311	8.470	13.743
	Water sprayed	12.453	1.311	9.817	15.090
	50OPs	19.493	1.311	16.857	22.130
	150OPs	20.580	1.311	17.944	23.216
	300OPs	32.540	1.311	29.904	35.176
	600OPs	34.997	1.311	32.360	37.633
25%FC	Cont	14.150	1.311	11.514	16.786
	Water sprayed	17.017	1.311	14.380	19.653
	50OPs	23.633	1.311	20.997	26.270
	150OPs	30.087	1.311	27.450	32.723
	300OPs	37.473	1.311	34.837	40.110
	600OPs	38.400	1.311	35.764	41.036

Post Hoc Tests

FC_LevOP

Homogeneous Subsets

H2O2_Sh

Duncan[a,b]

FC_LevOP	N	Subset			
		1	2	3	4
FFC	18	14.8439			
75%FC	18		18.5061		
50%FC	18			21.8617	
25%FC	18				26.7933
Sig.		1.000	1.000	1.000	1.000

Means for groups in homogeneous subsets are displayed.
Based on observed means.
The error term is Mean Square(Error) = 5.158.

a. Uses Harmonic Mean Sample Size = 18.000.

b. Alpha = .05.

OPs

Homogeneous Subsets

H2O2_Sh

Duncan[a,b]

OPs	N	Subset				
		1	2	3	4	5
Cont	12	10.3867				
Water sprayed	12	11.4883				
50OPs	12		19.7975			
150OPs	12			21.8875		
300OPs	12				28.5992	
600OPs	12					30.8483
Sig.		.241	1.000	1.000	1.000	1.000

Means for groups in homogeneous subsets are displayed.
Based on observed means.
The error term is Mean Square(Error) = 5.158.

a. Uses Harmonic Mean Sample Size = 12.000.

b. Alpha = .05.

Profile Plots

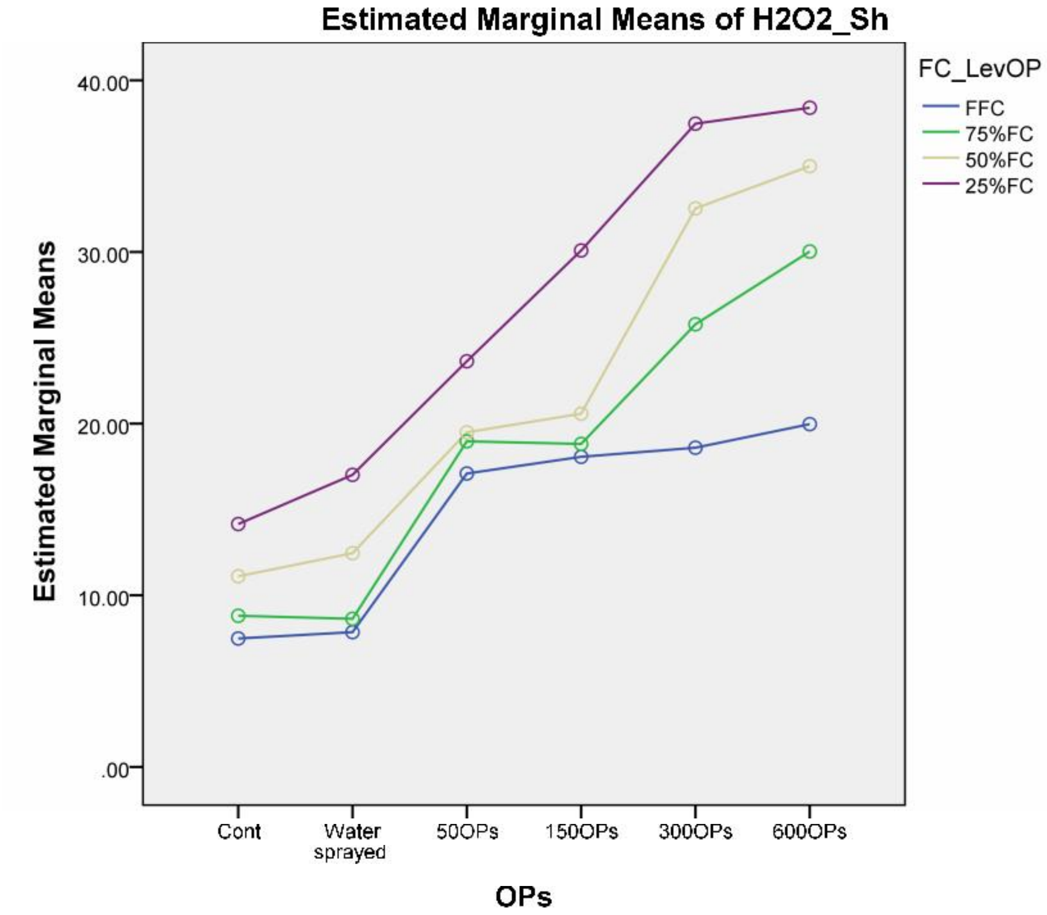


```
UNIANOVA H2O2_Ro BY FC_Levels NPs
  /METHOD=SSTYPE(3)
  /INTERCEPT=EXCLUDE
  /SAVE=SEPRED
  /POSTHOC=FC_Levels NPs(DUNCAN)
  /PLOT=PROFILE(NPs*FC_Levels)
  /EMMEANS=TABLES(OVERALL)
  /EMMEANS=TABLES(FC_Levels)
  /EMMEANS=TABLES(NPs)
  /EMMEANS=TABLES(FC_Levels*NPs)
  /PRINT=DESCRIPTIVE
  /CRITERIA=ALPHA(.05)
  /DESIGN=FC_Levels NPs FC_Levels*NPs.
```

Univariate Analysis of Variance

[DataSet1] E:\Amna M Sc\Paper\Two Way ANOVA H.sav

Between-Subjects Factors

		Value Label	N
FC_Levels	1	FFC	18
	2	75%FC	18
	3	50%FC	18
	4	25%FC	18
NPs	1	Cont	12
	2	Water sprayed	12
	3	50NP	12
	4	150NP	12
	5	300NP	12
	6	600NP	12

Descriptive Statistics

Dependent Variable: H2O2_Ro

FC_Levels	NPs	Mean	Std. Deviation	N
FFC	Cont	4.4567	1.03471	3
	Water sprayed	4.3700	.30447	3
	50NP	7.2133	.36088	3
	150NP	8.7533	1.04711	3
	300NP	5.9333	.78907	3
	600NP	1.7300	.22068	3
	Total	5.4094	2.38664	18
75%FC	Cont	5.3133	1.39672	3
	Water sprayed	4.8900	.71042	3
	50NP	7.5567	.65516	3
	150NP	10.0533	1.32217	3
	300NP	4.9033	.84097	3
	600NP	4.1667	.56898	3
	Total	6.1472	2.25308	18
50%FC	Cont	7.1033	.39552	3
	Water sprayed	6.1400	.29513	3
	50NP	10.1733	.20108	3
	150NP	9.9900	.36373	3
	300NP	6.5267	.47353	3
	600NP	5.1900	2.27502	3
	Total	7.5206	2.12183	18
25%FC	Cont	9.9567	1.43855	3
	Water sprayed	8.2733	1.50434	3
	50NP	10.9567	.77597	3
	150NP	10.8400	.67089	3
	300NP	10.2833	1.03751	3
	600NP	5.6033	.21385	3
	Total	9.3189	2.12421	18

Descriptive Statistics

Dependent Variable: H2O2_Ro

FC_Levels	NPs	Mean	Std. Deviation	N
Total	Cont	6.7075	2.40579	12
	Water sprayed	5.9183	1.73323	12
	50NP	8.9750	1.75392	12
	150NP	9.9092	1.10959	12
	300NP	6.9117	2.23163	12
	600NP	4.1725	1.86677	12
	Total	7.0990	2.64300	72

Tests of Between-Subjects Effects

Dependent Variable: H2O2_Ro

Source	Type III Sum of Squares	df	Mean Square	F	Sig.
Model	4082.395[a]	24	170.100	193.954	.000
FC_Levels	159.590	3	53.197	60.657	.000
NPs	258.758	5	51.752	59.009	.000
FC_Levels * NPs	35.521	15	2.368	2.700	.005
Error	42.097	48	.877		
Total	4124.491	72			

a. R Squared = .990 (Adjusted R Squared = .985)

Estimated Marginal Means

1. Grand Mean

Dependent Variable: H2O2_Ro

Mean	Std. Error	95% Confidence Interval Lower Bound	95% Confidence Interval Upper Bound
7.099	.110	6.877	7.321

2. FC_Levels

Dependent Variable: H2O2_Ro

FC_Levels	Mean	Std. Error	95% Confidence Interval Lower Bound	95% Confidence Interval Upper Bound
FFC	5.409	.221	4.966	5.853
75%FC	6.147	.221	5.703	6.591
50%FC	7.521	.221	7.077	7.964
25%FC	9.319	.221	8.875	9.763

3. NPs

Dependent Variable: H2O2_Ro

NPs	Mean	Std. Error	95% Confidence Interval	
			Lower Bound	Upper Bound
Cont	6.708	.270	6.164	7.251
Water sprayed	5.918	.270	5.375	6.462
50NP	8.975	.270	8.431	9.519
150NP	9.909	.270	9.366	10.453
300NP	6.912	.270	6.368	7.455
600NP	4.173	.270	3.629	4.716

4. FC_Levels * NPs

Dependent Variable: H2O2_Ro

FC_Levels	NPs	Mean	Std. Error	95% Confidence Interval	
				Lower Bound	Upper Bound
FFC	Cont	4.457	.541	3.370	5.544
	Water sprayed	4.370	.541	3.283	5.457
	50NP	7.213	.541	6.126	8.300
	150NP	8.753	.541	7.666	9.840
	300NP	5.933	.541	4.846	7.020
	600NP	1.730	.541	.643	2.817
75%FC	Cont	5.313	.541	4.226	6.400
	Water sprayed	4.890	.541	3.803	5.977
	50NP	7.557	.541	6.470	8.644
	150NP	10.053	.541	8.966	11.140
	300NP	4.903	.541	3.816	5.990
	600NP	4.167	.541	3.080	5.254
50%FC	Cont	7.103	.541	6.016	8.190
	Water sprayed	6.140	.541	5.053	7.227
	50NP	10.173	.541	9.086	11.260
	150NP	9.990	.541	8.903	11.077
	300NP	6.527	.541	5.440	7.614
	600NP	5.190	.541	4.103	6.277
25%FC	Cont	9.957	.541	8.870	11.044
	Water sprayed	8.273	.541	7.186	9.360
	50NP	10.957	.541	9.870	12.044
	150NP	10.840	.541	9.753	11.927
	300NP	10.283	.541	9.196	11.370
	600NP	5.603	.541	4.516	6.690

Post Hoc Tests

FC_Levels

Homogeneous Subsets

H2O2_Ro

Duncan[a,b]

FC_Levels	N	Subset			
		1	2	3	4
FFC	18	5.4094			
75%FC	18		6.1472		
50%FC	18			7.5206	
25%FC	18				9.3189
Sig.		1.000	1.000	1.000	1.000

Means for groups in homogeneous subsets are displayed.
Based on observed means.
The error term is Mean Square(Error) = .877.

a. Uses Harmonic Mean Sample Size = 18.000.

b. Alpha = .05.

NPs

Homogeneous Subsets

H2O2_Ro

Duncan[a,b]

NPs	N	Subset				
		1	2	3	4	5
600NP	12	4.1725				
Water sprayed	12		5.9183			
Cont	12			6.7075		
300NP	12			6.9117		
50NP	12				8.9750	
150NP	12					9.9092
Sig.		1.000	1.000	.596	1.000	1.000

Means for groups in homogeneous subsets are displayed.
Based on observed means.
The error term is Mean Square(Error) = .877.

a. Uses Harmonic Mean Sample Size = 12.000.

b. Alpha = .05.

Profile Plots

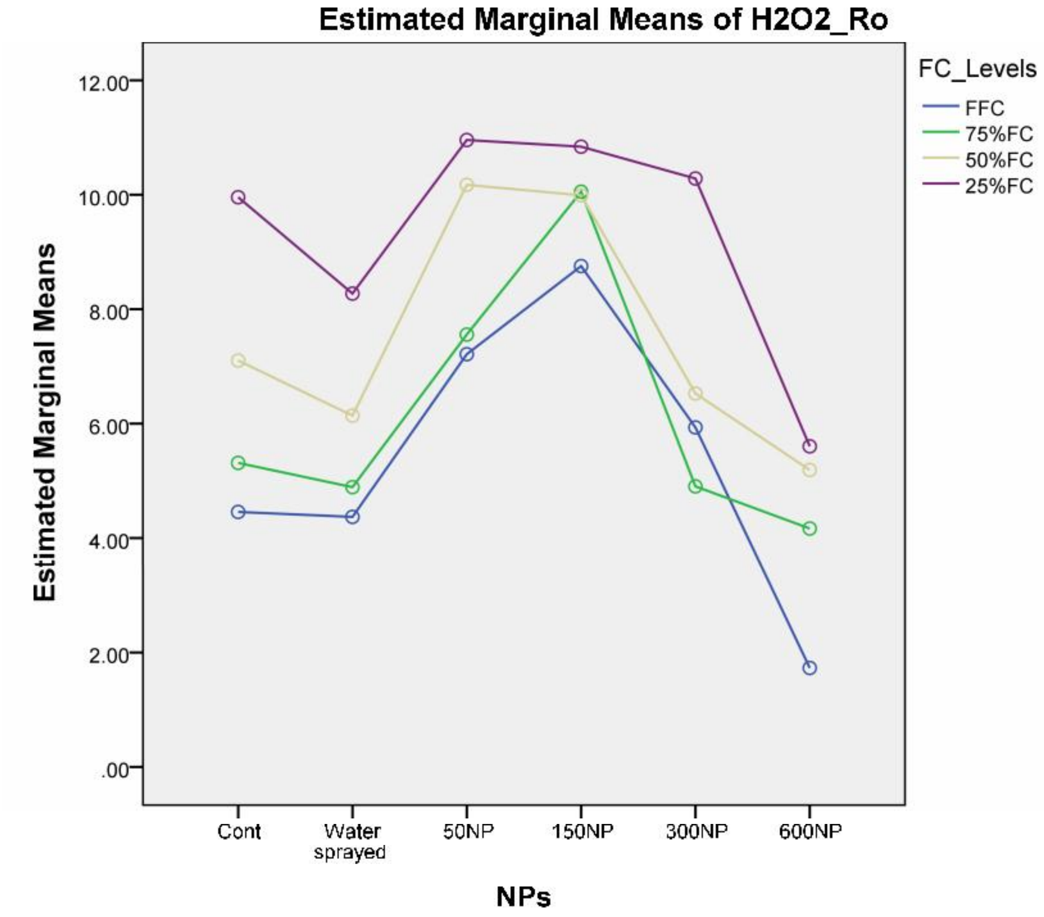


```
UNIANOVA H2O2_Ro BY FC_LevOP OPs
  /METHOD=SSTYPE(3)
  /INTERCEPT=EXCLUDE
  /SAVE=SEPRED
  /POSTHOC=FC_LevOP OPs(DUNCAN)
  /PLOT=PROFILE(OPs*FC_LevOP)
  /EMMEANS=TABLES(OVERALL)
  /EMMEANS=TABLES(FC_LevOP)
  /EMMEANS=TABLES(OPs)
  /EMMEANS=TABLES(FC_LevOP*OPs)
  /PRINT=DESCRIPTIVE
  /CRITERIA=ALPHA(.05)
  /DESIGN=FC_LevOP OPs FC_LevOP*OPs.
```

Univariate Analysis of Variance

```
[DataSet1] E:\Amna M Sc\Paper\Two Way ANOVA H.sav
```

Between-Subjects Factors

		Value Label	N
FC_LevOP	1.00	FFC	18
	2.00	75%FC	18
	3.00	50%FC	18
	4.00	25%FC	18
OPs	1.00	Cont	12
	2.00	Water sprayed	12
	3.00	50OPs	12
	4.00	150OPs	12
	5.00	300OPs	12
	6.00	600OPs	12

Descriptive Statistics

Dependent Variable: H2O2_Ro

FC_LevOP	OPs	Mean	Std. Deviation	N
FFC	Cont	4.4567	1.03471	3
	Water sprayed	4.3700	.30447	3
	50OPs	3.9567	.65187	3
	150OPs	3.3867	.82972	3
	300OPs	3.8267	.36910	3
	600OPs	6.6233	1.36152	3
	Total	4.4367	1.28355	18
75%FC	Cont	5.3133	1.39672	3
	Water sprayed	4.8900	.71042	3
	50OPs	4.2233	.32517	3
	150OPs	4.9600	.40632	3
	300OPs	5.5233	1.22916	3
	600OPs	7.5367	.32316	3
	Total	5.4078	1.28249	18
50%FC	Cont	7.1033	.39552	3
	Water sprayed	6.1400	.29513	3
	50OPs	4.6567	.80002	3
	150OPs	5.4300	1.02942	3
	300OPs	6.3900	.67000	3
	600OPs	10.3933	.71248	3
	Total	6.6856	1.96737	18
25%FC	Cont	9.9567	1.43855	3
	Water sprayed	8.2733	1.50434	3
	50OPs	5.4400	1.08761	3
	150OPs	5.8867	.74782	3
	300OPs	7.8800	1.29549	3
	600OPs	13.1300	1.18507	3
	Total	8.4278	2.85723	18

Descriptive Statistics

Dependent Variable: H2O2_Ro

FC_LevOP	OPs	Mean	Std. Deviation	N
Total	Cont	6.7075	2.40579	12
	Water sprayed	5.9183	1.73323	12
	50OPs	4.5692	.87851	12
	150OPs	4.9158	1.19036	12
	300OPs	5.9050	1.74125	12
	600OPs	9.4208	2.79577	12
	Total	6.2394	2.43589	72

Tests of Between-Subjects Effects

Dependent Variable: H2O2_Ro

Source	Type III Sum of Squares	df	Mean Square	F	Sig.
Model	3182.960[a]	24	132.623	154.024	.000
FC_LevOP	160.731	3	53.577	62.222	.000
OPs	181.165	5	36.233	42.080	.000
FC_LevOP * OPs	38.056	15	2.537	2.946	.002
Error	41.331	48	.861		
Total	3224.291	72			

a. R Squared = .987 (Adjusted R Squared = .981)

Estimated Marginal Means

1. Grand Mean

Dependent Variable: H2O2_Ro

Mean	Std. Error	95% Confidence Interval	
		Lower Bound	Upper Bound
6.239	.109	6.020	6.459

2. FC_LevOP

Dependent Variable: H2O2_Ro

FC_LevOP	Mean	Std. Error	95% Confidence Interval	
			Lower Bound	Upper Bound
FFC	4.437	.219	3.997	4.876
75%FC	5.408	.219	4.968	5.848
50%FC	6.686	.219	6.246	7.125
25%FC	8.428	.219	7.988	8.868

3. OPs

Dependent Variable: H2O2_Ro

OPs	Mean	Std. Error	95% Confidence Interval	
			Lower Bound	Upper Bound
Cont	6.708	.268	6.169	7.246
Water sprayed	5.918	.268	5.380	6.457
50OPs	4.569	.268	4.031	5.108
150OPs	4.916	.268	4.377	5.454
300OPs	5.905	.268	5.366	6.444
600OPs	9.421	.268	8.882	9.959

4. FC_LevOP * OPs

Dependent Variable: H2O2_Ro

FC_LevOP	OPs	Mean	Std. Error	95% Confidence Interval	
				Lower Bound	Upper Bound
FFC	Cont	4.457	.536	3.379	5.534
	Water sprayed	4.370	.536	3.293	5.447
	50OPs	3.957	.536	2.879	5.034
	150OPs	3.387	.536	2.309	4.464
	300OPs	3.827	.536	2.749	4.904
	600OPs	6.623	.536	5.546	7.701
75%FC	Cont	5.313	.536	4.236	6.391
	Water sprayed	4.890	.536	3.813	5.967
	50OPs	4.223	.536	3.146	5.301
	150OPs	4.960	.536	3.883	6.037
	300OPs	5.523	.536	4.446	6.601
	600OPs	7.537	.536	6.459	8.614
50%FC	Cont	7.103	.536	6.026	8.181
	Water sprayed	6.140	.536	5.063	7.217
	50OPs	4.657	.536	3.579	5.734
	150OPs	5.430	.536	4.353	6.507
	300OPs	6.390	.536	5.313	7.467
	600OPs	10.393	.536	9.316	11.471
25%FC	Cont	9.957	.536	8.879	11.034
	Water sprayed	8.273	.536	7.196	9.351
	50OPs	5.440	.536	4.363	6.517
	150OPs	5.887	.536	4.809	6.964
	300OPs	7.880	.536	6.803	8.957
	600OPs	13.130	.536	12.053	14.207

Post Hoc Tests

FC_LevOP

Homogeneous Subsets

H2O2_Ro

Duncan[a,b]

FC_LevOP	N	Subset 1	2	3	4
FFC	18	4.4367			
75%FC	18		5.4078		
50%FC	18			6.6856	
25%FC	18				8.4278
Sig.		1.000	1.000	1.000	1.000

Means for groups in homogeneous subsets are displayed.
Based on observed means.
The error term is Mean Square(Error) = .861.

a. Uses Harmonic Mean Sample Size = 18.000.

b. Alpha = .05.

OPs

Homogeneous Subsets

H2O2_Ro

Duncan[a,b]

OPs	N	Subset 1	2	3	4
50OPs	12	4.5692			
150OPs	12	4.9158			
300OPs	12		5.9050		
Water sprayed	12		5.9183		
Cont	12			6.7075	
600OPs	12				9.4208
Sig.		.365	.972	1.000	1.000

Means for groups in homogeneous subsets are displayed.
Based on observed means.
The error term is Mean Square(Error) = .861.

a. Uses Harmonic Mean Sample Size = 12.000.

b. Alpha = .05.

Profile Plots

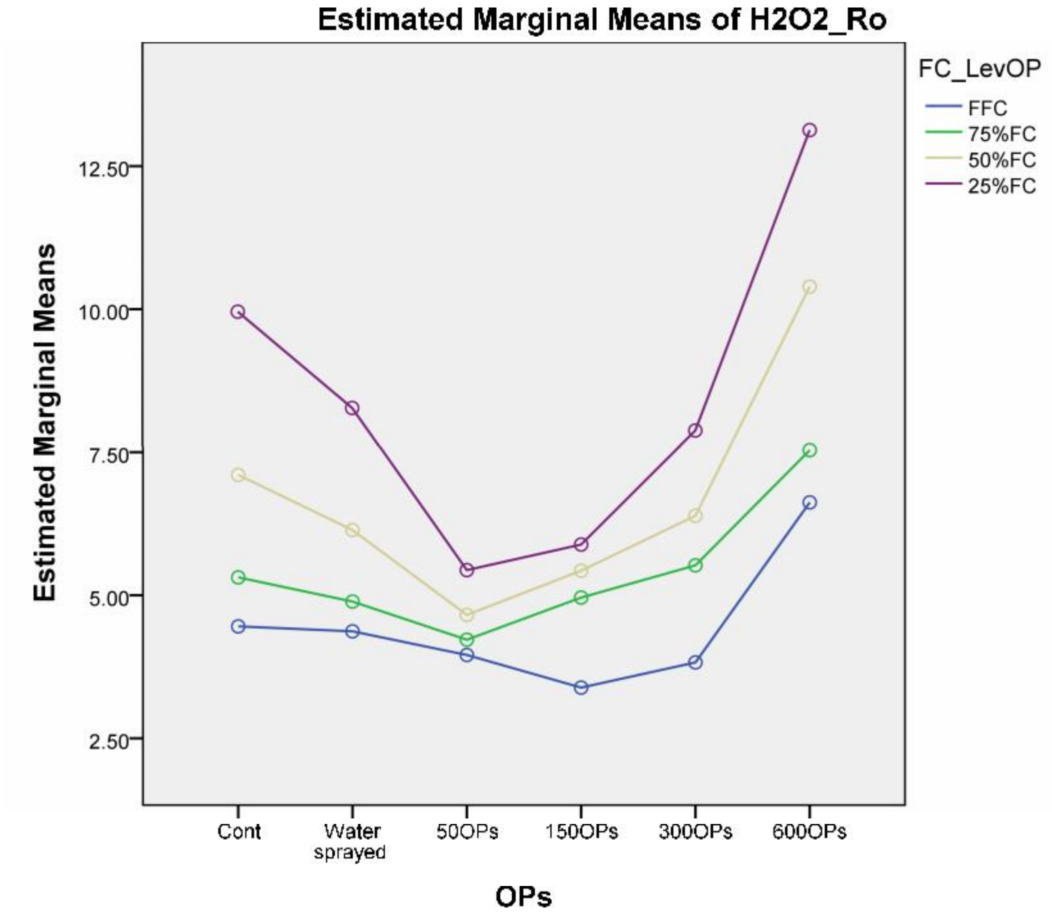


```
UNIANOVA LIP_PER_Sh BY FC_Levels NPs
  /METHOD=SSTYPE(3)
  /INTERCEPT=EXCLUDE
  /SAVE=SEPRED
  /POSTHOC=FC_Levels NPs(DUNCAN)
  /PLOT=PROFILE(NPs*FC_Levels)
  /EMMEANS=TABLES(OVERALL)
  /EMMEANS=TABLES(FC_Levels)
  /EMMEANS=TABLES(NPs)
  /EMMEANS=TABLES(FC_Levels*NPs)
  /PRINT=DESCRIPTIVE
  /CRITERIA=ALPHA(.05)
  /DESIGN=FC_Levels NPs FC_Levels*NPs.
```

Univariate Analysis of Variance

[DataSet1] E:\Amna M Sc\Paper\Two Way ANOVA H.sav

Between-Subjects Factors

		Value Label	N
FC_Levels	1	FFC	18
	2	75%FC	18
	3	50%FC	18
	4	25%FC	18
NPs	1	Cont	12
	2	Water sprayed	12
	3	50NP	12
	4	150NP	12
	5	300NP	12
	6	600NP	12

Descriptive Statistics

Dependent Variable: LIP_PER_Sh

FC_Levels	NPs	Mean	Std. Deviation	N
FFC	Cont	69.0467	9.42181	3
	Water sprayed	66.6700	6.32581	3
	50NP	60.2533	5.87627	3
	150NP	65.1800	3.25018	3
	300NP	69.2167	4.94484	3
	600NP	68.7700	10.99281	3
	Total	66.5228	6.93572	18
75%FC	Cont	71.5933	9.96842	3
	Water sprayed	70.0633	2.81617	3
	50NP	69.6800	8.30893	3
	150NP	73.4167	5.32200	3
	300NP	82.1633	1.32213	3
	600NP	82.2300	6.32045	3
	Total	74.8578	7.68294	18
50%FC	Cont	72.4833	13.85448	3
	Water sprayed	73.5033	4.92581	3
	50NP	78.5567	10.99007	3
	150NP	78.2167	15.32971	3
	300NP	86.5833	19.73776	3
	600NP	95.7967	12.76071	3
	Total	80.8567	14.19640	18
25%FC	Cont	86.0300	13.24765	3
	Water sprayed	82.8900	11.48751	3
	50NP	75.5433	1.59168	3
	150NP	82.5900	3.94476	3
	300NP	89.3833	3.03355	3
	600NP	77.5800	14.63640	3
	Total	82.3361	9.37862	18

Descriptive Statistics

Dependent Variable: LIP_PER_Sh

FC_Levels	NPs	Mean	Std. Deviation	N
Total	Cont	74.7883	12.19449	12
	Water sprayed	73.2817	8.77843	12
	50NP	71.0083	9.71738	12
	150NP	74.8508	9.90552	12
	300NP	81.8367	11.93362	12
	600NP	81.0942	14.20872	12
	Total	76.1433	11.58472	72

Tests of Between-Subjects Effects

Dependent Variable: LIP_PER_Sh

Source	Type III Sum of Squares	df	Mean Square	F	Sig.
Model	422492.873[a]	24	17603.870	188.704	.000
FC_Levels	2785.927	3	928.642	9.955	.000
NPs	1139.865	5	227.973	2.444	.047
FC_Levels * NPs	1124.961	15	74.997	.804	.668
Error	4477.847	48	93.288		
Total	426970.720	72			

a. R Squared = .990 (Adjusted R Squared = .984)

Estimated Marginal Means

1. Grand Mean

Dependent Variable: LIP_PER_Sh

Mean	Std. Error	95% Confidence Interval	
		Lower Bound	Upper Bound
76.143	1.138	73.855	78.432

2. FC_Levels

Dependent Variable: LIP_PER_Sh

FC_Levels	Mean	Std. Error	95% Confidence Interval	
			Lower Bound	Upper Bound
FFC	66.523	2.277	61.945	71.100
75%FC	74.858	2.277	70.280	79.435
50%FC	80.857	2.277	76.279	85.434
25%FC	82.336	2.277	77.759	86.913

3. NPs

Dependent Variable: LIP_PER_Sh

NPs	Mean	Std. Error	95% Confidence Interval	
			Lower Bound	Upper Bound
Cont	74.788	2.788	69.182	80.394
Water sprayed	73.282	2.788	67.676	78.888
50NP	71.008	2.788	65.402	76.614
150NP	74.851	2.788	69.245	80.457
300NP	81.837	2.788	76.231	87.443
600NP	81.094	2.788	75.488	86.700

4. FC_Levels * NPs

Dependent Variable: LIP_PER_Sh

FC_Levels	NPs	Mean	Std. Error	95% Confidence Interval	
				Lower Bound	Upper Bound
FFC	Cont	69.047	5.576	57.835	80.259
	Water sprayed	66.670	5.576	55.458	77.882
	50NP	60.253	5.576	49.041	71.465
	150NP	65.180	5.576	53.968	76.392
	300NP	69.217	5.576	58.005	80.429
	600NP	68.770	5.576	57.558	79.982
75%FC	Cont	71.593	5.576	60.381	82.805
	Water sprayed	70.063	5.576	58.851	81.275
	50NP	69.680	5.576	58.468	80.892
	150NP	73.417	5.576	62.205	84.629
	300NP	82.163	5.576	70.951	93.375
	600NP	82.230	5.576	71.018	93.442
50%FC	Cont	72.483	5.576	61.271	83.695
	Water sprayed	73.503	5.576	62.291	84.715
	50NP	78.557	5.576	67.345	89.769
	150NP	78.217	5.576	67.005	89.429
	300NP	86.583	5.576	75.371	97.795
	600NP	95.797	5.576	84.585	107.009
25%FC	Cont	86.030	5.576	74.818	97.242
	Water sprayed	82.890	5.576	71.678	94.102
	50NP	75.543	5.576	64.331	86.755
	150NP	82.590	5.576	71.378	93.802
	300NP	89.383	5.576	78.171	100.595
	600NP	77.580	5.576	66.368	88.792

Post Hoc Tests

FC_Levels

Homogeneous Subsets

LIP_PER_Sh

Duncan[a,b]

FC_Levels	N	Subset		
		1	2	3
FFC	18	66.5228		
75%FC	18		74.8578	
50%FC	18		80.8567	80.8567
25%FC	18			82.3361
Sig.		1.000	.069	.648

Means for groups in homogeneous subsets are displayed.
Based on observed means.
The error term is Mean Square(Error) = 93.288.

a. Uses Harmonic Mean Sample Size = 18.000.

b. Alpha = .05.

NPs

Homogeneous Subsets

LIP_PER_Sh

Duncan[a,b]

NPs	N	Subset	
		1	2
50NP	12	71.0083	
Water sprayed	12	73.2817	73.2817
Cont	12	74.7883	74.7883
150NP	12	74.8508	74.8508
600NP	12		81.0942
300NP	12		81.8367
Sig.		.382	.057

Means for groups in homogeneous subsets are displayed.
Based on observed means.
The error term is Mean Square(Error) = 93.288.

a. Uses Harmonic Mean Sample Size = 12.000.

b. Alpha = .05.

Profile Plots

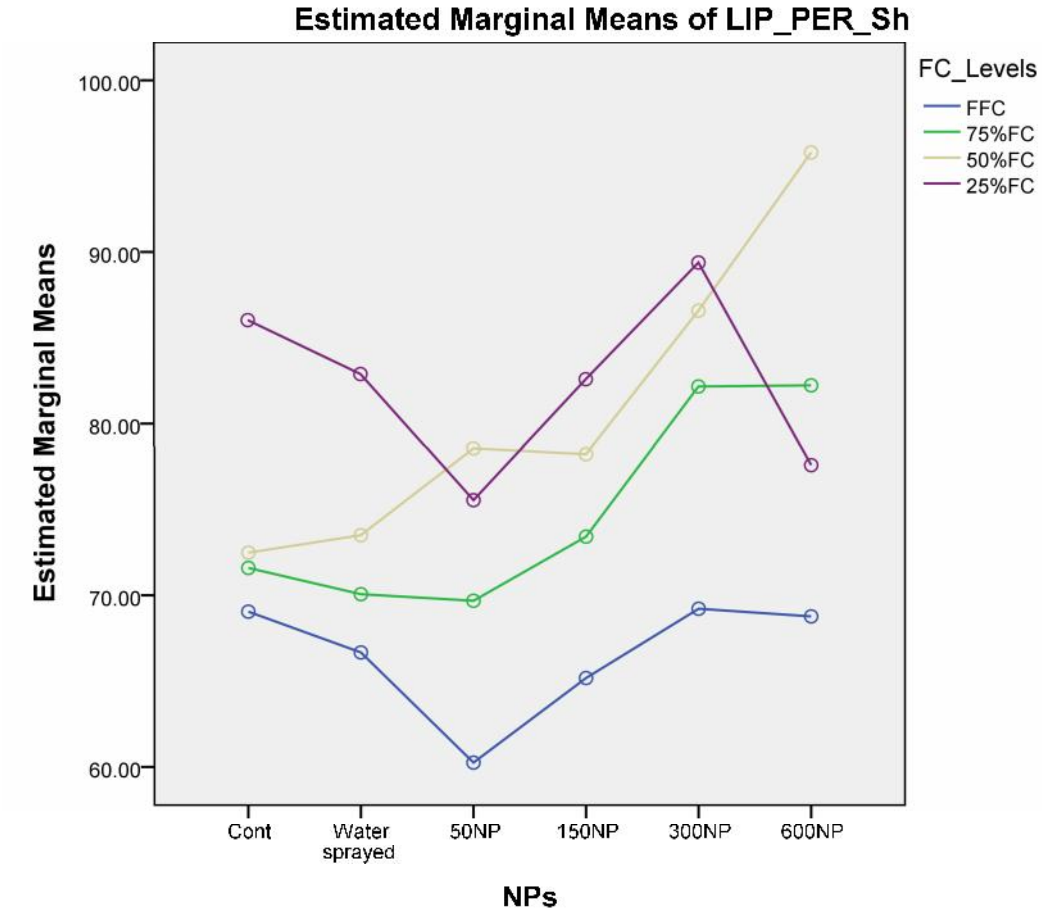


```
UNIANOVA LIP_PER_Sh BY FC_LevOP OPs
  /METHOD=SSTYPE(3)
  /INTERCEPT=EXCLUDE
  /SAVE=SEPRED
  /POSTHOC=FC_LevOP OPs(DUNCAN)
  /PLOT=PROFILE(OPs*FC_LevOP)
  /EMMEANS=TABLES(OVERALL)
  /EMMEANS=TABLES(FC_LevOP)
  /EMMEANS=TABLES(OPs)
  /EMMEANS=TABLES(FC_LevOP*OPs)
  /PRINT=DESCRIPTIVE
  /CRITERIA=ALPHA(.05)
  /DESIGN=FC_LevOP OPs FC_LevOP*OPs.
```

Univariate Analysis of Variance

```
[DataSet1] E:\Amna M Sc\Paper\Two Way ANOVA H.sav
```

Between-Subjects Factors

		Value Label	N
FC_LevOP	1.00	FFC	18
	2.00	75%FC	18
	3.00	50%FC	18
	4.00	25%FC	18
OPs	1.00	Cont	12
	2.00	Water sprayed	12
	3.00	50OPs	12
	4.00	150OPs	12
	5.00	300OPs	12
	6.00	600OPs	12

Descriptive Statistics

Dependent Variable: LIP_PER_Sh

FC_LevOP	OPs	Mean	Std. Deviation	N
FFC	Cont	69.0467	9.42181	3
	Water sprayed	66.6700	6.32581	3
	50OPs	75.7967	.21939	3
	150OPs	77.7500	2.91834	3
	300OPs	91.9733	5.95668	3
	600OPs	108.1100	3.64021	3
	Total	81.5578	15.51142	18
75%FC	Cont	71.5933	9.96842	3
	Water sprayed	70.0633	2.81617	3
	50OPs	71.4667	4.04233	3
	150OPs	80.6800	1.30369	3
	300OPs	99.8700	5.97492	3
	600OPs	104.6300	3.23894	3
	Total	83.0506	15.16184	18
50%FC	Cont	72.4833	13.85448	3
	Water sprayed	73.5033	4.92581	3
	50OPs	88.0267	3.83278	3
	150OPs	91.4667	8.54571	3
	300OPs	106.1733	7.74009	3
	600OPs	120.9367	9.34207	3
	Total	92.0983	19.14955	18
25%FC	Cont	86.0300	13.24765	3
	Water sprayed	82.8900	11.48751	3
	50OPs	89.1300	1.36121	3
	150OPs	101.7833	9.57403	3
	300OPs	109.3000	.91804	3
	600OPs	123.0967	2.02466	3
	Total	98.7050	16.21409	18

Descriptive Statistics

Dependent Variable: LIP_PER_Sh

FC_LevOP	OPs	Mean	Std. Deviation	N
Total	Cont	74.7883	12.19449	12
	Water sprayed	73.2817	8.77843	12
	50OPs	81.1050	8.34496	12
	150OPs	87.9200	11.40840	12
	300OPs	101.8292	8.47912	12
	600OPs	114.1933	9.48611	12
	Total	88.8529	17.68352	72

Tests of Between-Subjects Effects

Dependent Variable: LIP_PER_Sh

Source	Type III Sum of Squares	df	Mean Square	F	Sig.
Model	588201.420[a]	24	24508.393	484.257	.000
FC_LevOP	3500.689	3	1166.896	23.056	.000
OPs	15740.360	5	3148.072	62.202	.000
FC_LevOP * OPs	531.834	15	35.456	.701	.771
Error	2429.296	48	50.610		
Total	590630.716	72			

a. R Squared = .996 (Adjusted R Squared = .994)

Estimated Marginal Means

1. Grand Mean

Dependent Variable: LIP_PER_Sh

Mean	Std. Error	95% Confidence Interval	
		Lower Bound	Upper Bound
88.853	.838	87.167	90.539

2. FC_LevOP

Dependent Variable: LIP_PER_Sh

FC_LevOP	Mean	Std. Error	95% Confidence Interval	
			Lower Bound	Upper Bound
FFC	81.558	1.677	78.186	84.929
75%FC	83.051	1.677	79.679	86.422
50%FC	92.098	1.677	88.727	95.470
25%FC	98.705	1.677	95.334	102.076

3. OPs

Dependent Variable: LIP_PER_Sh

OPs	Mean	Std. Error	95% Confidence Interval	
			Lower Bound	Upper Bound
Cont	74.788	2.054	70.659	78.917
Water sprayed	73.282	2.054	69.153	77.411
50OPs	81.105	2.054	76.976	85.234
150OPs	87.920	2.054	83.791	92.049
300OPs	101.829	2.054	97.700	105.958
600OPs	114.193	2.054	110.064	118.322

4. FC_LevOP * OPs

Dependent Variable: LIP_PER_Sh

FC_LevOP	OPs	Mean	Std. Error	95% Confidence Interval	
				Lower Bound	Upper Bound
FFC	Cont	69.047	4.107	60.788	77.305
	Water sprayed	66.670	4.107	58.412	74.928
	50OPs	75.797	4.107	67.538	84.055
	150OPs	77.750	4.107	69.492	86.008
	300OPs	91.973	4.107	83.715	100.232
	600OPs	108.110	4.107	99.852	116.368
75%FC	Cont	71.593	4.107	63.335	79.852
	Water sprayed	70.063	4.107	61.805	78.322
	50OPs	71.467	4.107	63.208	79.725
	150OPs	80.680	4.107	72.422	88.938
	300OPs	99.870	4.107	91.612	108.128
	600OPs	104.630	4.107	96.372	112.888
50%FC	Cont	72.483	4.107	64.225	80.742
	Water sprayed	73.503	4.107	65.245	81.762
	50OPs	88.027	4.107	79.768	96.285
	150OPs	91.467	4.107	83.208	99.725
	300OPs	106.173	4.107	97.915	114.432
	600OPs	120.937	4.107	112.678	129.195
25%FC	Cont	86.030	4.107	77.772	94.288
	Water sprayed	82.890	4.107	74.632	91.148
	50OPs	89.130	4.107	80.872	97.388
	150OPs	101.783	4.107	93.525	110.042
	300OPs	109.300	4.107	101.042	117.558
	600OPs	123.097	4.107	114.838	131.355

Post Hoc Tests

FC_LevOP

Homogeneous Subsets

LIP_PER_Sh

Duncan[a,b]

FC_LevOP	N	Subset		
		1	2	3
FFC	18	81.5578		
75%FC	18	83.0506		
50%FC	18		92.0983	
25%FC	18			98.7050
Sig.		.532	1.000	1.000

Means for groups in homogeneous subsets are displayed.
Based on observed means.
The error term is Mean Square(Error) = 50.610.

a. Uses Harmonic Mean Sample Size = 18.000.

b. Alpha = .05.

OPs

Homogeneous Subsets

LIP_PER_Sh

Duncan[a,b]

OPs	N	Subset				
		1	2	3	4	5
Water sprayed	12	73.2817				
Cont	12	74.7883				
50OPs	12		81.1050			
150OPs	12			87.9200		
300OPs	12				101.8292	
600OPs	12					114.1933
Sig.		.606	1.000	1.000	1.000	1.000

Means for groups in homogeneous subsets are displayed.
Based on observed means.
The error term is Mean Square(Error) = 50.610.

a. Uses Harmonic Mean Sample Size = 12.000.

b. Alpha = .05.

Profile Plots

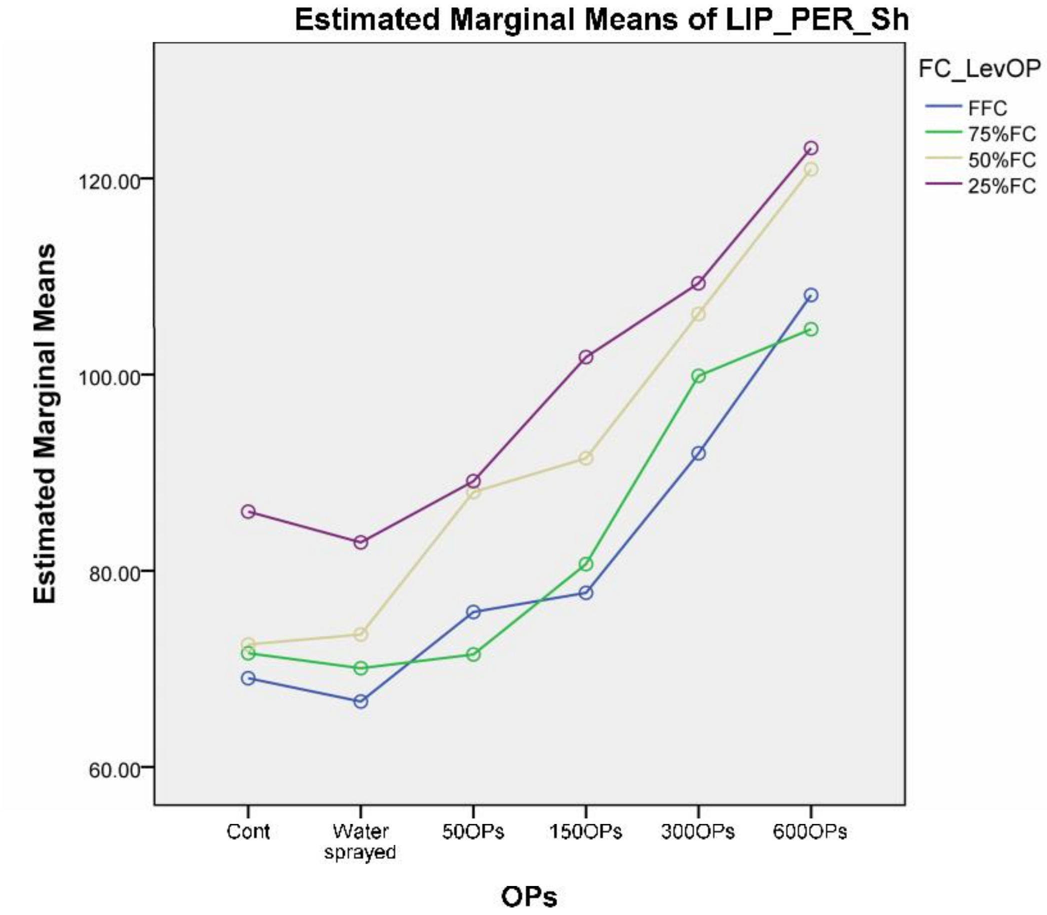


```
UNIANOVA Proline BY FC_Levels NPs
  /METHOD=SSTYPE(3)
  /INTERCEPT=EXCLUDE
  /SAVE=SEPRED
  /POSTHOC=FC_Levels NPs(DUNCAN)
  /PLOT=PROFILE(NPs*FC_Levels)
  /EMMEANS=TABLES(OVERALL)
  /EMMEANS=TABLES(FC_Levels)
  /EMMEANS=TABLES(NPs)
  /EMMEANS=TABLES(FC_Levels*NPs)
  /PRINT=DESCRIPTIVE
  /CRITERIA=ALPHA(.05)
  /DESIGN=FC_Levels NPs FC_Levels*NPs.
```

Univariate Analysis of Variance

[DataSet1] E:\Amna M Sc\Paper\Two Way ANOVA H.sav

Between-Subjects Factors

		Value Label	N
FC_Levels	1	FFC	18
	2	75%FC	18
	3	50%FC	18
	4	25%FC	18
NPs	1	Cont	12
	2	Water sprayed	12
	3	50NP	12
	4	150NP	12
	5	300NP	12
	6	600NP	12

Descriptive Statistics

Dependent Variable: Proline

FC_Levels	NPs	Mean	Std. Deviation	N
FFC	Cont	39.3000	1.03923	3
	Water sprayed	37.4667	2.80416	3
	50NP	47.1000	1.80000	3
	150NP	47.7333	5.84323	3
	300NP	114.1667	4.81906	3
	600NP	103.3000	1.80000	3
	Total	64.8444	32.45745	18
75%FC	Cont	68.8667	11.06406	3
	Water sprayed	58.0000	4.76235	3
	50NP	77.9000	1.80000	3
	150NP	80.9667	2.80416	3
	300NP	120.8333	6.36894	3
	600NP	174.5667	6.87192	3
	Total	96.8556	41.33402	18
50%FC	Cont	133.5333	7.53414	3
	Water sprayed	64.6333	2.79344	3
	50NP	82.1667	2.79344	3
	150NP	81.5667	9.05005	3
	300NP	128.0667	13.56773	3
	600NP	259.1667	4.81906	3
	Total	124.8556	67.33035	18
25%FC	Cont	222.9333	4.81906	3
	Water sprayed	212.0667	3.17543	3
	50NP	205.4000	5.19615	3
	150NP	192.7000	2.74955	3
	300NP	222.9333	11.00651	3
	600NP	408.4000	3.74700	3
	Total	244.0722	76.53337	18

Descriptive Statistics

Dependent Variable: Proline

FC_Levels	NPs	Mean	Std. Deviation	N
Total	Cont	116.1583	73.82193	12
	Water sprayed	93.0417	72.59371	12
	50NP	103.1417	63.32180	12
	150NP	100.7417	57.47552	12
	300NP	146.5000	47.09393	12
	600NP	236.3583	118.74316	12
	Total	132.6569	88.33174	72

Tests of Between-Subjects Effects

Dependent Variable: Proline

Source	Type III Sum of Squares	df	Mean Square	F	Sig.
Model	1819260.72[a]	24	75802.530	2064.050	.000
FC_Levels	330381.007	3	110127.002	2998.682	.000
NPs	176123.031	5	35224.606	959.142	.000
FC_Levels * NPs	45710.412	15	3047.361	82.978	.000
Error	1762.807	48	36.725		
Total	1821023.530	72			

a. R Squared = .999 (Adjusted R Squared = .999)

Estimated Marginal Means

1. Grand Mean

Dependent Variable: Proline

Mean	Std. Error	95% Confidence Interval	
		Lower Bound	Upper Bound
132.657	.714	131.221	134.093

2. FC_Levels

Dependent Variable: Proline

FC_Levels	Mean	Std. Error	95% Confidence Interval	
			Lower Bound	Upper Bound
FFC	64.844	1.428	61.972	67.716
75%FC	96.856	1.428	93.984	99.728
50%FC	124.856	1.428	121.984	127.728
25%FC	244.072	1.428	241.200	246.944

3. NPs

Dependent Variable: Proline

NPs	Mean	Std. Error	95% Confidence Interval	
			Lower Bound	Upper Bound
Cont	116.158	1.749	112.641	119.676
Water sprayed	93.042	1.749	89.524	96.559
50NP	103.142	1.749	99.624	106.659
150NP	100.742	1.749	97.224	104.259
300NP	146.500	1.749	142.983	150.017
600NP	236.358	1.749	232.841	239.876

4. FC_Levels * NPs

Dependent Variable: Proline

FC_Levels	NPs	Mean	Std. Error	95% Confidence Interval	
				Lower Bound	Upper Bound
FFC	Cont	39.300	3.499	32.265	46.335
	Water sprayed	37.467	3.499	30.432	44.502
	50NP	47.100	3.499	40.065	54.135
	150NP	47.733	3.499	40.698	54.768
	300NP	114.167	3.499	107.132	121.202
	600NP	103.300	3.499	96.265	110.335
75%FC	Cont	68.867	3.499	61.832	75.902
	Water sprayed	58.000	3.499	50.965	65.035
	50NP	77.900	3.499	70.865	84.935
	150NP	80.967	3.499	73.932	88.002
	300NP	120.833	3.499	113.798	127.868
	600NP	174.567	3.499	167.532	181.602
50%FC	Cont	133.533	3.499	126.498	140.568
	Water sprayed	64.633	3.499	57.598	71.668
	50NP	82.167	3.499	75.132	89.202
	150NP	81.567	3.499	74.532	88.602
	300NP	128.067	3.499	121.032	135.102
	600NP	259.167	3.499	252.132	266.202
25%FC	Cont	222.933	3.499	215.898	229.968
	Water sprayed	212.067	3.499	205.032	219.102
	50NP	205.400	3.499	198.365	212.435
	150NP	192.700	3.499	185.665	199.735
	300NP	222.933	3.499	215.898	229.968
	600NP	408.400	3.499	401.365	415.435

Post Hoc Tests

FC_Levels

Homogeneous Subsets

Proline

Duncan[a,b]

FC_Levels	N	Subset			
		1	2	3	4
FFC	18	64.8444			
75%FC	18		96.8556		
50%FC	18			124.8556	
25%FC	18				244.0722
Sig.		1.000	1.000	1.000	1.000

Means for groups in homogeneous subsets are displayed.
Based on observed means.
The error term is Mean Square(Error) = 36.725.

a. Uses Harmonic Mean Sample Size = 18.000.

b. Alpha = .05.

NPs

Homogeneous Subsets

Proline

Duncan[a,b]

NPs	N	Subset				
		1	2	3	4	5
Water sprayed	12	93.0417				
150NP	12		100.7417			
50NP	12		103.1417			
Cont	12			116.1583		
300NP	12				146.5000	
600NP	12					236.3583
Sig.		1.000	.337	1.000	1.000	1.000

Means for groups in homogeneous subsets are displayed.
Based on observed means.
The error term is Mean Square(Error) = 36.725.

a. Uses Harmonic Mean Sample Size = 12.000.

b. Alpha = .05.

Profile Plots

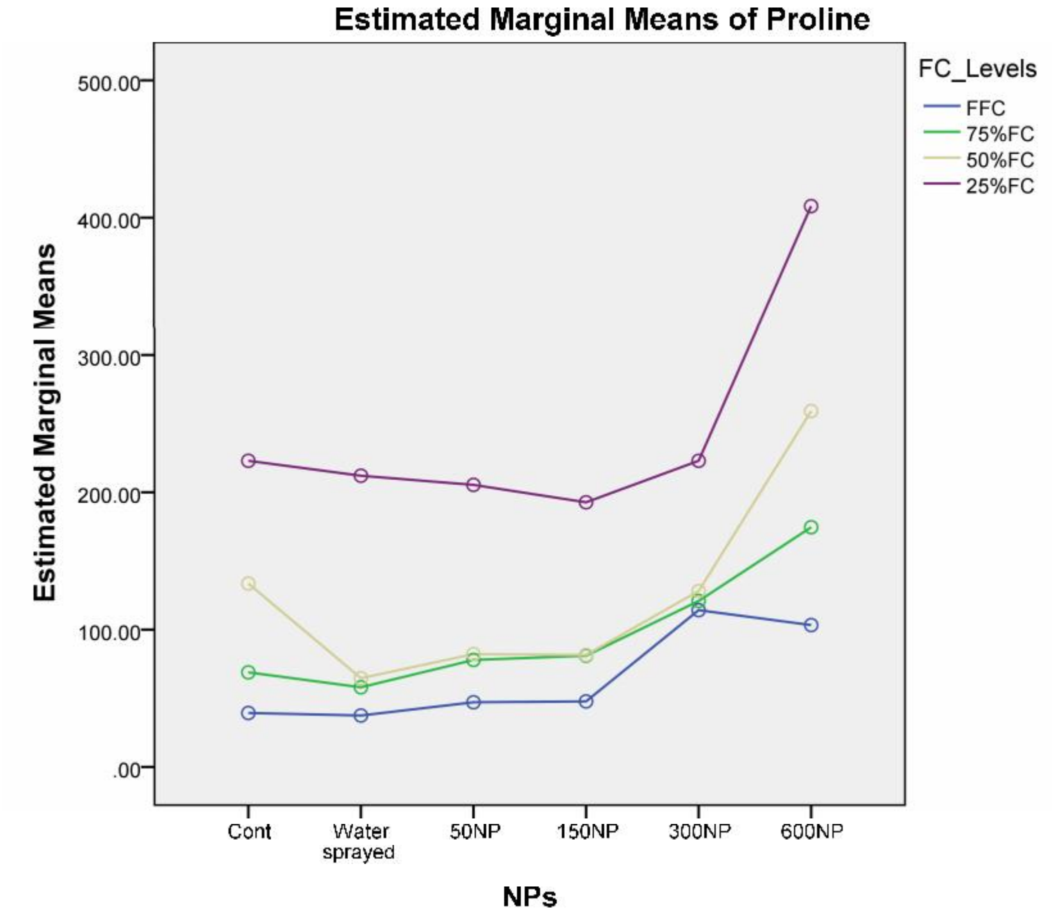


```
UNIANOVA Proline BY FC_LevOP OPs
  /METHOD=SSTYPE(3)
  /INTERCEPT=EXCLUDE
  /SAVE=SEPRED
  /POSTHOC=FC_LevOP OPs(DUNCAN)
  /PLOT=PROFILE(OPs*FC_LevOP)
  /EMMEANS=TABLES(OVERALL)
  /EMMEANS=TABLES(FC_LevOP)
  /EMMEANS=TABLES(OPs)
  /EMMEANS=TABLES(FC_LevOP*OPs)
  /PRINT=DESCRIPTIVE
  /CRITERIA=ALPHA(.05)
  /DESIGN=FC_LevOP OPs FC_LevOP*OPs.
```

Univariate Analysis of Variance

```
[DataSet1] E:\Amna M Sc\Paper\Two Way ANOVA H.sav
```

Between-Subjects Factors

		Value Label	N
FC_LevOP	1.00	FFC	18
	2.00	75%FC	18
	3.00	50%FC	18
	4.00	25%FC	18
OPs	1.00	Cont	12
	2.00	Water sprayed	12
	3.00	50OPs	12
	4.00	150OPs	12
	5.00	300OPs	12
	6.00	600OPs	12

Descriptive Statistics

Dependent Variable: Proline

FC_LevOP	OPs	Mean	Std. Deviation	N
FFC	Cont	39.3000	1.03923	3
	Water sprayed	37.4667	2.80416	3
	50OPs	35.0333	3.78726	3
	150OPs	88.2000	5.78619	3
	300OPs	69.4667	40.38940	3
	600OPs	68.8333	7.90337	3
	Total	56.3833	25.24353	18
75%FC	Cont	68.8667	11.06406	3
	Water sprayed	58.0000	4.76235	3
	50OPs	64.6667	5.84323	3
	150OPs	140.1667	3.80307	3
	300OPs	84.6000	2.74955	3
	600OPs	80.9333	5.55368	3
	Total	82.8722	28.46161	18
50%FC	Cont	133.5333	7.53414	3
	Water sprayed	64.6333	2.79344	3
	50OPs	99.6667	6.54548	3
	150OPs	143.1667	6.54548	3
	300OPs	134.1000	3.60000	3
	600OPs	92.4333	3.65011	3
	Total	111.2556	29.15392	18
25%FC	Cont	222.9333	4.81906	3
	Water sprayed	212.0667	3.17543	3
	50OPs	211.4667	1.09697	3
	150OPs	210.2667	4.81906	3
	300OPs	178.2333	10.60393	3
	600OPs	387.8333	7.90337	3
	Total	237.1333	70.97477	18

Descriptive Statistics

Dependent Variable: Proline

FC_LevOP	OPs	Mean	Std. Deviation	N
Total	Cont	116.1583	73.82193	12
	Water sprayed	93.0417	72.59371	12
	50OPs	102.7083	69.92149	12
	150OPs	145.4500	45.49095	12
	300OPs	116.6000	48.22291	12
	600OPs	157.5083	139.27482	12
	Total	121.9111	81.39857	72

Tests of Between-Subjects Effects

Dependent Variable: Proline

Source	Type III Sum of Squares	df	Mean Square	F	Sig.
Model	1535729.95[a]	24	63988.748	642.078	.000
FC_LevOP	345737.266	3	115245.755	1156.402	.000
OPs	37016.824	5	7403.365	74.287	.000
FC_LevOP * OPs	82888.894	15	5525.926	55.448	.000
Error	4783.627	48	99.659		
Total	1540513.580	72			

a. R Squared = .997 (Adjusted R Squared = .995)

Estimated Marginal Means

1. Grand Mean

Dependent Variable: Proline

Mean	Std. Error	95% Confidence Interval	
		Lower Bound	Upper Bound
121.911	1.176	119.546	124.277

2. FC_LevOP

Dependent Variable: Proline

FC_LevOP	Mean	Std. Error	95% Confidence Interval	
			Lower Bound	Upper Bound
FFC	56.383	2.353	51.652	61.114
75%FC	82.872	2.353	78.141	87.603
50%FC	111.256	2.353	106.525	115.987
25%FC	237.133	2.353	232.402	241.864

3. OPs

Dependent Variable: Proline

OPs	Mean	Std. Error	95% Confidence Interval	
			Lower Bound	Upper Bound
Cont	116.158	2.882	110.364	121.953
Water sprayed	93.042	2.882	87.247	98.836
50OPs	102.708	2.882	96.914	108.503
150OPs	145.450	2.882	139.656	151.244
300OPs	116.600	2.882	110.806	122.394
600OPs	157.508	2.882	151.714	163.303

4. FC_LevOP * OPs

Dependent Variable: Proline

FC_LevOP	OPs	Mean	Std. Error	95% Confidence Interval	
				Lower Bound	Upper Bound
FFC	Cont	39.300	5.764	27.711	50.889
	Water sprayed	37.467	5.764	25.878	49.055
	50OPs	35.033	5.764	23.445	46.622
	150OPs	88.200	5.764	76.611	99.789
	300OPs	69.467	5.764	57.878	81.055
	600OPs	68.833	5.764	57.245	80.422
75%FC	Cont	68.867	5.764	57.278	80.455
	Water sprayed	58.000	5.764	46.411	69.589
	50OPs	64.667	5.764	53.078	76.255
	150OPs	140.167	5.764	128.578	151.755
	300OPs	84.600	5.764	73.011	96.189
	600OPs	80.933	5.764	69.345	92.522
50%FC	Cont	133.533	5.764	121.945	145.122
	Water sprayed	64.633	5.764	53.045	76.222
	50OPs	99.667	5.764	88.078	111.255
	150OPs	143.167	5.764	131.578	154.755
	300OPs	134.100	5.764	122.511	145.689
	600OPs	92.433	5.764	80.845	104.022
25%FC	Cont	222.933	5.764	211.345	234.522
	Water sprayed	212.067	5.764	200.478	223.655
	50OPs	211.467	5.764	199.878	223.055
	150OPs	210.267	5.764	198.678	221.855
	300OPs	178.233	5.764	166.645	189.822
	600OPs	387.833	5.764	376.245	399.422

Post Hoc Tests

FC_LevOP

Homogeneous Subsets

Proline

Duncan[a,b]

FC_LevOP	N	Subset 1	2	3	4
FFC	18	56.3833			
75%FC	18		82.8722		
50%FC	18			111.2556	
25%FC	18				237.1333
Sig.		1.000	1.000	1.000	1.000

Means for groups in homogeneous subsets are displayed.
Based on observed means.
The error term is Mean Square(Error) = 99.659.

a. Uses Harmonic Mean Sample Size = 18.000.

b. Alpha = .05.

OPs

Homogeneous Subsets

Proline

Duncan[a,b]

OPs	N	Subset 1	2	3	4	5
Water sprayed	12	93.0417				
50OPs	12		102.7083			
Cont	12			116.1583		
300OPs	12			116.6000		
150OPs	12				145.4500	
600OPs	12					157.5083
Sig.		1.000	1.000	.914	1.000	1.000

Means for groups in homogeneous subsets are displayed.
Based on observed means.
The error term is Mean Square(Error) = 99.659.

a. Uses Harmonic Mean Sample Size = 12.000.

b. Alpha = .05.

Profile Plots

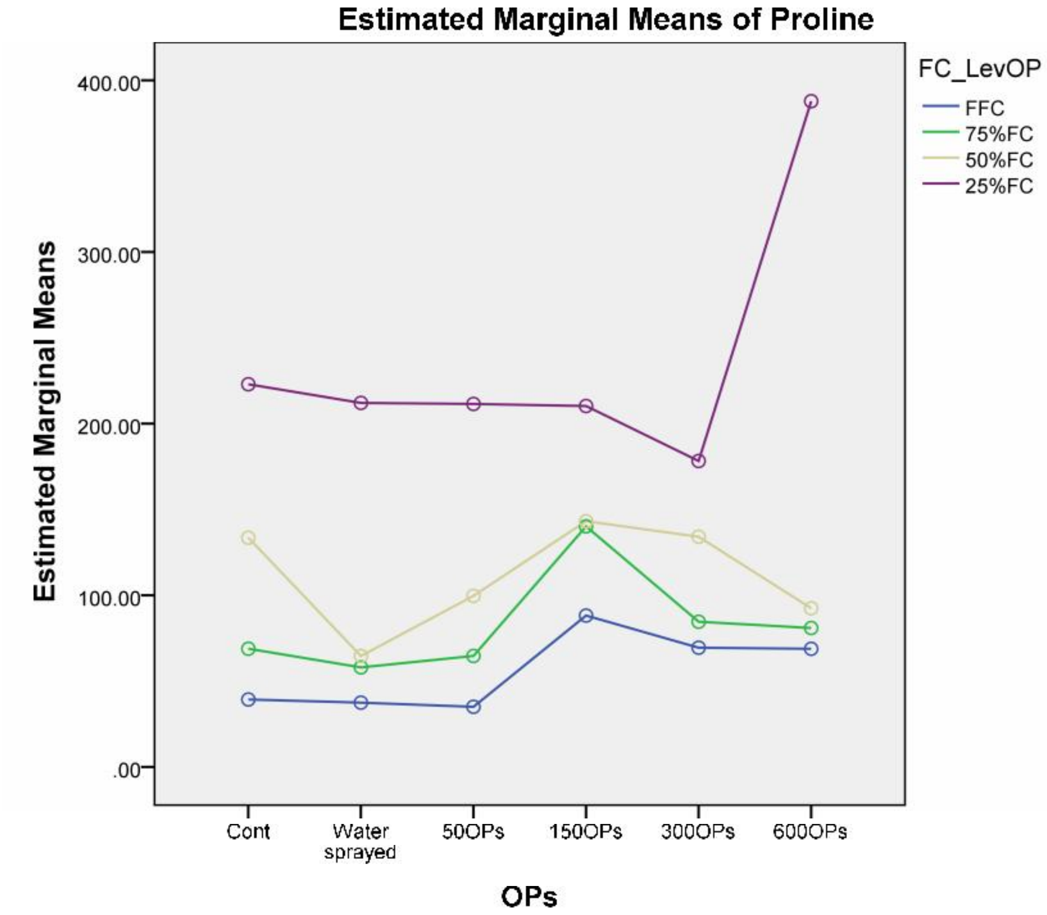


```
UNIANOVA Catalase_Sh BY FC_Levels NPs
  /METHOD=SSTYPE(3)
  /INTERCEPT=EXCLUDE
  /SAVE=SEPRED
  /POSTHOC=FC_Levels NPs(DUNCAN)
  /PLOT=PROFILE(NPs*FC_Levels)
  /EMMEANS=TABLES(OVERALL)
  /EMMEANS=TABLES(FC_Levels)
  /EMMEANS=TABLES(NPs)
  /EMMEANS=TABLES(FC_Levels*NPs)
  /PRINT=DESCRIPTIVE
  /CRITERIA=ALPHA(.05)
  /DESIGN=FC_Levels NPs FC_Levels*NPs.
```

Univariate Analysis of Variance

```
[DataSet1] E:\Amna M Sc\Paper\Two Way ANOVA H.sav
```

Between-Subjects Factors

		Value Label	N
FC_Levels	1	FFC	18
	2	75%FC	18
	3	50%FC	18
	4	25%FC	18
NPs	1	Cont	12
	2	Water sprayed	12
	3	50NP	12
	4	150NP	12
	5	300NP	12
	6	600NP	12

Descriptive Statistics

Dependent Variable: Catalase_Sh

FC_Levels	NPs	Mean	Std. Deviation	N
FFC	Cont	1.0677	.13221	3
	Water sprayed	3.9093	.23325	3
	50NP	1.4427	.24050	3
	150NP	1.3250	.13510	3
	300NP	2.1587	.26950	3
	600NP	2.0737	.29650	3
	Total	1.9962	.98673	18
75%FC	Cont	1.0097	.25250	3
	Water sprayed	3.4750	.66857	3
	50NP	2.0267	.42203	3
	150NP	2.0997	.26250	3
	300NP	4.0857	.29150	3
	600NP	3.9277	.52907	3
	Total	2.7707	1.21877	18
50%FC	Cont	3.1230	.38625	3
	Water sprayed	4.6830	.39000	3
	50NP	4.8510	.50507	3
	150NP	4.5333	.39923	3
	300NP	5.6790	.44687	3
	600NP	5.7970	.71707	3
	Total	4.7777	.99556	18
25%FC	Cont	3.6060	.27193	3
	Water sprayed	5.9740	.73959	3
	50NP	6.4493	.69859	3
	150NP	6.0960	.27700	3
	300NP	7.4457	.55045	3
	600NP	6.2380	.77894	3
	Total	5.9682	1.29346	18

Descriptive Statistics

Dependent Variable: Catalase_Sh

FC_Levels	NPs	Mean	Std. Deviation	N
Total	Cont	2.2016	1.25017	12
	Water sprayed	4.5103	1.09614	12
	50NP	3.6924	2.18039	12
	150NP	3.5135	2.00318	12
	300NP	4.8423	2.06874	12
	600NP	4.5091	1.80225	12
	Total	3.8782	1.93601	72

Tests of Between-Subjects Effects

Dependent Variable: Catalase_Sh

Source	Type III Sum of Squares	df	Mean Square	F	Sig.
Model	1339.112[a]	24	55.796	270.186	.000
FC_Levels	179.022	3	59.674	288.963	.000
NPs	56.467	5	11.293	54.686	.000
FC_Levels * NPs	20.715	15	1.381	6.687	.000
Error	9.913	48	.207		
Total	1349.025	72			

a. R Squared = .993 (Adjusted R Squared = .989)

Estimated Marginal Means

1. Grand Mean

Dependent Variable: Catalase_Sh

Mean	Std. Error	95% Confidence Interval	
		Lower Bound	Upper Bound
3.878	.054	3.771	3.986

2. FC_Levels

Dependent Variable: Catalase_Sh

FC_Levels	Mean	Std. Error	95% Confidence Interval	
			Lower Bound	Upper Bound
FFC	1.996	.107	1.781	2.212
75%FC	2.771	.107	2.555	2.986
50%FC	4.778	.107	4.562	4.993
25%FC	5.968	.107	5.753	6.184

3. NPs

Dependent Variable: Catalase_Sh

NPs	Mean	Std. Error	95% Confidence Interval	
			Lower Bound	Upper Bound
Cont	2.202	.131	1.938	2.465
Water sprayed	4.510	.131	4.247	4.774
50NP	3.692	.131	3.429	3.956
150NP	3.514	.131	3.250	3.777
300NP	4.842	.131	4.578	5.106
600NP	4.509	.131	4.245	4.773

4. FC_Levels * NPs

Dependent Variable: Catalase_Sh

FC_Levels	NPs	Mean	Std. Error	95% Confidence Interval	
				Lower Bound	Upper Bound
FFC	Cont	1.068	.262	.540	1.595
	Water sprayed	3.909	.262	3.382	4.437
	50NP	1.443	.262	.915	1.970
	150NP	1.325	.262	.797	1.853
	300NP	2.159	.262	1.631	2.686
	600NP	2.074	.262	1.546	2.601
75%FC	Cont	1.010	.262	.482	1.537
	Water sprayed	3.475	.262	2.947	4.003
	50NP	2.027	.262	1.499	2.554
	150NP	2.100	.262	1.572	2.627
	300NP	4.086	.262	3.558	4.613
	600NP	3.928	.262	3.400	4.455
50%FC	Cont	3.123	.262	2.595	3.651
	Water sprayed	4.683	.262	4.155	5.211
	50NP	4.851	.262	4.323	5.379
	150NP	4.533	.262	4.006	5.061
	300NP	5.679	.262	5.151	6.207
	600NP	5.797	.262	5.269	6.325
25%FC	Cont	3.606	.262	3.078	4.134
	Water sprayed	5.974	.262	5.446	6.502
	50NP	6.449	.262	5.922	6.977
	150NP	6.096	.262	5.568	6.624
	300NP	7.446	.262	6.918	7.973
	600NP	6.238	.262	5.710	6.766

Post Hoc Tests

FC_Levels

Homogeneous Subsets

Catalase_Sh

Duncan[a,b]

FC_Levels	N	Subset			
		1	2	3	4
FFC	18	1.9962			
75%FC	18		2.7707		
50%FC	18			4.7777	
25%FC	18				5.9682
Sig.		1.000	1.000	1.000	1.000

Means for groups in homogeneous subsets are displayed.
Based on observed means.
The error term is Mean Square(Error) = .207.

a. Uses Harmonic Mean Sample Size = 18.000.

b. Alpha = .05.

NPs

Homogeneous Subsets

Catalase_Sh

Duncan[a,b]

NPs	N	Subset		
		1	2	3
Cont	12	2.2016		
150NP	12		3.5135	
50NP	12		3.6924	
600NP	12			4.5091
Water sprayed	12			4.5103
300NP	12			4.8423
Sig.		1.000	.340	.095

Means for groups in homogeneous subsets are displayed.
Based on observed means.
The error term is Mean Square(Error) = .207.

a. Uses Harmonic Mean Sample Size = 12.000.

b. Alpha = .05.

Profile Plots

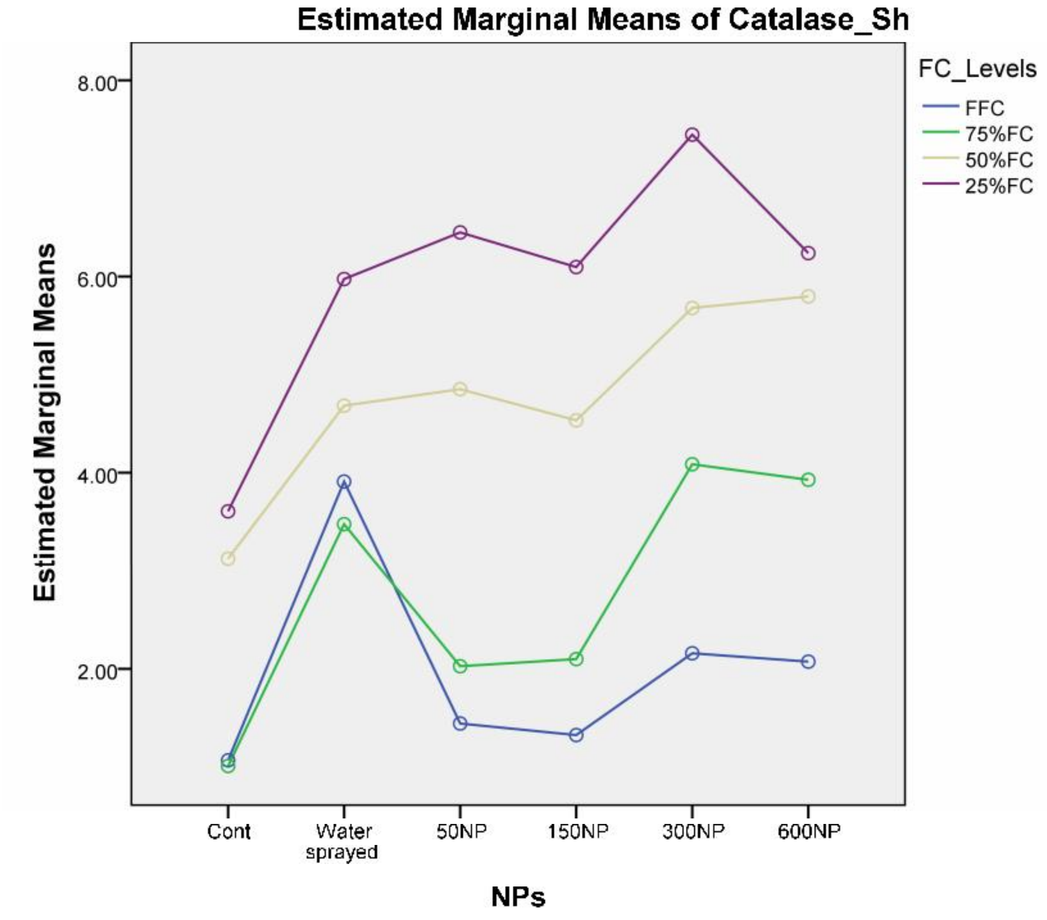


```
UNIANOVA Catalase_Sh BY FC_LevOP OPs
  /METHOD=SSTYPE(3)
  /INTERCEPT=EXCLUDE
  /SAVE=SEPRED
  /POSTHOC=FC_LevOP OPs(DUNCAN)
  /PLOT=PROFILE(OPs*FC_LevOP)
  /EMMEANS=TABLES(OVERALL)
  /EMMEANS=TABLES(FC_LevOP)
  /EMMEANS=TABLES(OPs)
  /EMMEANS=TABLES(FC_LevOP*OPs)
  /PRINT=DESCRIPTIVE
  /CRITERIA=ALPHA(.05)
  /DESIGN=FC_LevOP OPs FC_LevOP*OPs.
```

Univariate Analysis of Variance

[DataSet1] E:\Amna M Sc\Paper\Two Way ANOVA H.sav

Between-Subjects Factors

		Value Label	N
FC_LevOP	1.00	FFC	18
	2.00	75%FC	18
	3.00	50%FC	18
	4.00	25%FC	18
OPs	1.00	Cont	12
	2.00	Water sprayed	12
	3.00	50OPs	12
	4.00	150OPs	12
	5.00	300OPs	12
	6.00	600OPs	12

Descriptive Statistics

Dependent Variable: Catalase_Sh

FC_LevOP	OPs	Mean	Std. Deviation	N
FFC	Cont	1.0677	.13221	3
	Water sprayed	3.9093	.23325	3
	50OPs	4.0717	.22055	3
	150OPs	2.8727	.19110	3
	300OPs	3.0377	.40472	3
	600OPs	3.3440	.55700	3
	Total	3.0505	1.05027	18
75%FC	Cont	1.0097	.25250	3
	Water sprayed	3.4750	.66857	3
	50OPs	3.4100	.60130	3
	150OPs	3.5370	.23556	3
	300OPs	3.8573	.58081	3
	600OPs	3.5383	.43763	3
	Total	3.1379	1.07310	18
50%FC	Cont	3.1230	.38625	3
	Water sprayed	4.6830	.39000	3
	50OPs	5.6937	.17956	3
	150OPs	5.7833	.20034	3
	300OPs	6.1010	.49190	3
	600OPs	4.9377	.74420	3
	Total	5.0536	1.08711	18
25%FC	Cont	3.6060	.27193	3
	Water sprayed	5.9740	.73959	3
	50OPs	7.6243	.57446	3
	150OPs	7.2620	.78635	3
	300OPs	6.9523	.69251	3
	600OPs	6.5250	.45207	3
	Total	6.3239	1.45631	18

Descriptive Statistics

Dependent Variable: Catalase_Sh

FC_LevOP	OPs	Mean	Std. Deviation	N
Total	Cont	2.2016	1.25017	12
	Water sprayed	4.5103	1.09614	12
	50OPs	5.1999	1.74095	12
	150OPs	4.8638	1.87002	12
	300OPs	4.9871	1.73163	12
	600OPs	4.5863	1.41712	12
	Total	4.3915	1.80078	72

Tests of Between-Subjects Effects

Dependent Variable: Catalase_Sh

Source	Type III Sum of Squares	df	Mean Square	F	Sig.
Model	1607.749[a]	24	66.990	291.763	.000
FC_LevOP	135.766	3	45.255	197.103	.000
OPs	72.949	5	14.590	63.544	.000
FC_LevOP * OPs	10.503	15	.700	3.050	.002
Error	11.021	48	.230		
Total	1618.770	72			

a. R Squared = .993 (Adjusted R Squared = .990)

Estimated Marginal Means

1. Grand Mean

Dependent Variable: Catalase_Sh

Mean	Std. Error	95% Confidence Interval	
		Lower Bound	Upper Bound
4.391	.056	4.278	4.505

2. FC_LevOP

Dependent Variable: Catalase_Sh

FC_LevOP	Mean	Std. Error	95% Confidence Interval	
			Lower Bound	Upper Bound
FFC	3.051	.113	2.823	3.278
75%FC	3.138	.113	2.911	3.365
50%FC	5.054	.113	4.827	5.281
25%FC	6.324	.113	6.097	6.551

3. OPs

Dependent Variable: Catalase_Sh

OPs	Mean	Std. Error	95% Confidence Interval	
			Lower Bound	Upper Bound
Cont	2.202	.138	1.923	2.480
Water sprayed	4.510	.138	4.232	4.788
50OPs	5.200	.138	4.922	5.478
150OPs	4.864	.138	4.586	5.142
300OPs	4.987	.138	4.709	5.265
600OPs	4.586	.138	4.308	4.864

4. FC_LevOP * OPs

Dependent Variable: Catalase_Sh

FC_LevOP	OPs	Mean	Std. Error	95% Confidence Interval	
				Lower Bound	Upper Bound
FFC	Cont	1.068	.277	.511	1.624
	Water sprayed	3.909	.277	3.353	4.466
	50OPs	4.072	.277	3.515	4.628
	150OPs	2.873	.277	2.316	3.429
	300OPs	3.038	.277	2.481	3.594
	600OPs	3.344	.277	2.788	3.900
75%FC	Cont	1.010	.277	.453	1.566
	Water sprayed	3.475	.277	2.919	4.031
	50OPs	3.410	.277	2.854	3.966
	150OPs	3.537	.277	2.981	4.093
	300OPs	3.857	.277	3.301	4.414
	600OPs	3.538	.277	2.982	4.095
50%FC	Cont	3.123	.277	2.567	3.679
	Water sprayed	4.683	.277	4.127	5.239
	50OPs	5.694	.277	5.137	6.250
	150OPs	5.783	.277	5.227	6.340
	300OPs	6.101	.277	5.545	6.657
	600OPs	4.938	.277	4.381	5.494
25%FC	Cont	3.606	.277	3.050	4.162
	Water sprayed	5.974	.277	5.418	6.530
	50OPs	7.624	.277	7.068	8.181
	150OPs	7.262	.277	6.706	7.818
	300OPs	6.952	.277	6.396	7.509
	600OPs	6.525	.277	5.969	7.081

Post Hoc Tests

FC_LevOP

Homogeneous Subsets

Catalase_Sh

Duncan[a,b]

FC_LevOP	N	Subset 1	2	3
FFC	18	3.0505		
75%FC	18	3.1379		
50%FC	18		5.0536	
25%FC	18			6.3239
Sig.		.587	1.000	1.000

Means for groups in homogeneous subsets are displayed.
Based on observed means.
The error term is Mean Square(Error) = .230.

a. Uses Harmonic Mean Sample Size = 18.000.

b. Alpha = .05.

OPs

Homogeneous Subsets

Catalase_Sh

Duncan[a,b]

OPs	N	Subset 1	2	3	4
Cont	12	2.2016			
Water sprayed	12		4.5103		
600OPs	12		4.5863	4.5863	
150OPs	12		4.8638	4.8638	4.8638
300OPs	12			4.9871	4.9871
50OPs	12				5.1999
Sig.		1.000	.093	.057	.111

Means for groups in homogeneous subsets are displayed.
Based on observed means.
The error term is Mean Square(Error) = .230.

a. Uses Harmonic Mean Sample Size = 12.000.

b. Alpha = .05.

Profile Plots

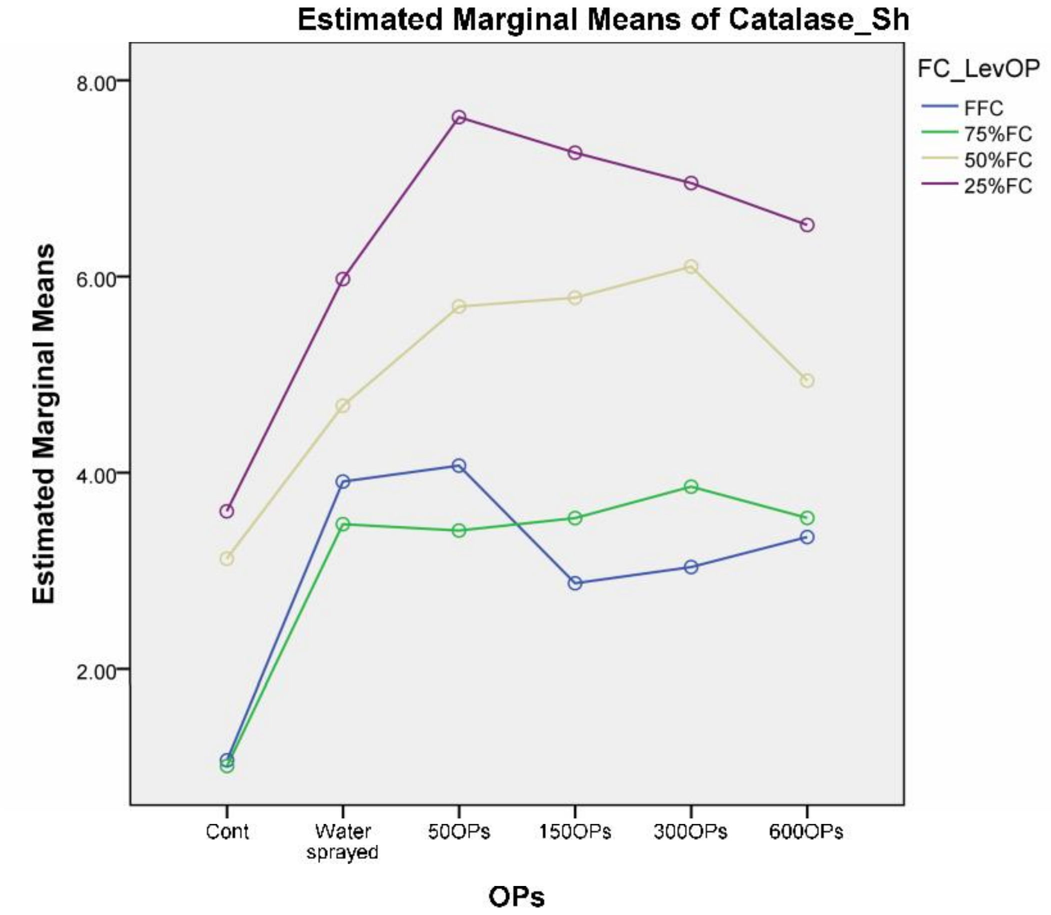


```
UNIANOVA Peroxidase_Sh BY FC_Levels NPs
  /METHOD=SSTYPE(3)
  /INTERCEPT=EXCLUDE
  /SAVE=SEPRED
  /POSTHOC=FC_Levels NPs(DUNCAN)
  /PLOT=PROFILE(NPs*FC_Levels)
  /EMMEANS=TABLES(OVERALL)
  /EMMEANS=TABLES(FC_Levels)
  /EMMEANS=TABLES(NPs)
  /EMMEANS=TABLES(FC_Levels*NPs)
  /PRINT=DESCRIPTIVE
  /CRITERIA=ALPHA(.05)
  /DESIGN=FC_Levels NPs FC_Levels*NPs.
```

Univariate Analysis of Variance

[DataSet1] E:\Amna M Sc\Paper\Two Way ANOVA H.sav

Between-Subjects Factors

		Value Label	N
FC_Levels	1	FFC	18
	2	75%FC	18
	3	50%FC	18
	4	25%FC	18
NPs	1	Cont	12
	2	Water sprayed	12
	3	50NP	12
	4	150NP	12
	5	300NP	12
	6	600NP	12

Descriptive Statistics

Dependent Variable: Peroxidase_Sh

FC_Levels	NPs	Mean	Std. Deviation	N
FFC	Cont	.7467	.12702	3
	Water sprayed	1.4933	.19858	3
	50NP	4.2833	.38553	3
	150NP	4.1633	.04619	3
	300NP	5.2433	.20207	3
	600NP	5.3400	.43578	3
	Total	3.5450	1.84862	18
75%FC	Cont	1.1767	.04619	3
	Water sprayed	1.7200	.19313	3
	50NP	5.2500	.18735	3
	150NP	5.5300	.21656	3
	300NP	7.0700	.14731	3
	600NP	6.7733	.49095	3
	Total	4.5867	2.39043	18
50%FC	Cont	2.4200	.15133	3
	Water sprayed	4.4933	.07506	3
	50NP	6.8267	.06351	3
	150NP	6.7333	.38734	3
	300NP	10.0100	.06928	3
	600NP	10.6567	.35529	3
	Total	6.8567	2.96682	18
25%FC	Cont	3.5267	.26558	3
	Water sprayed	6.7633	.21385	3
	50NP	8.1733	.16743	3
	150NP	8.0633	.27934	3
	300NP	12.0500	.29206	3
	600NP	12.6033	.30730	3
	Total	8.5300	3.19236	18

Descriptive Statistics

Dependent Variable: Peroxidase_Sh

FC_Levels	NPs	Mean	Std. Deviation	N
Total	Cont	1.9675	1.14726	12
	Water sprayed	3.6175	2.26804	12
	50NP	6.1333	1.56574	12
	150NP	6.1225	1.52382	12
	300NP	8.5933	2.74360	12
	600NP	8.8433	3.06380	12
	Total	5.8796	3.25001	72

Tests of Between-Subjects Effects

Dependent Variable: Peroxidase_Sh

Source	Type III Sum of Squares	df	Mean Square	F	Sig.
Model	3235.874[a]	24	134.828	2105.410	.000
FC_Levels	271.824	3	90.608	1414.888	.000
NPs	440.317	5	88.063	1375.154	.000
FC_Levels * NPs	34.730	15	2.315	36.155	.000
Error	3.074	48	.064		
Total	3238.948	72			

a. R Squared = .999 (Adjusted R Squared = .999)

Estimated Marginal Means

1. Grand Mean

Dependent Variable: Peroxidase_Sh

Mean	Std. Error	95% Confidence Interval	
		Lower Bound	Upper Bound
5.880	.030	5.820	5.940

2. FC_Levels

Dependent Variable: Peroxidase_Sh

FC_Levels	Mean	Std. Error	95% Confidence Interval	
			Lower Bound	Upper Bound
FFC	3.545	.060	3.425	3.665
75%FC	4.587	.060	4.467	4.707
50%FC	6.857	.060	6.737	6.977
25%FC	8.530	.060	8.410	8.650

3. NPs

Dependent Variable: Peroxidase_Sh

NPs	Mean	Std. Error	95% Confidence Interval	
			Lower Bound	Upper Bound
Cont	1.968	.073	1.821	2.114
Water sprayed	3.618	.073	3.471	3.764
50NP	6.133	.073	5.986	6.280
150NP	6.123	.073	5.976	6.269
300NP	8.593	.073	8.446	8.740
600NP	8.843	.073	8.696	8.990

4. FC_Levels * NPs

Dependent Variable: Peroxidase_Sh

FC_Levels	NPs	Mean	Std. Error	95% Confidence Interval	
				Lower Bound	Upper Bound
FFC	Cont	.747	.146	.453	1.040
	Water sprayed	1.493	.146	1.200	1.787
	50NP	4.283	.146	3.990	4.577
	150NP	4.163	.146	3.870	4.457
	300NP	5.243	.146	4.950	5.537
	600NP	5.340	.146	5.046	5.634
75%FC	Cont	1.177	.146	.883	1.470
	Water sprayed	1.720	.146	1.426	2.014
	50NP	5.250	.146	4.956	5.544
	150NP	5.530	.146	5.236	5.824
	300NP	7.070	.146	6.776	7.364
	600NP	6.773	.146	6.480	7.067
50%FC	Cont	2.420	.146	2.126	2.714
	Water sprayed	4.493	.146	4.200	4.787
	50NP	6.827	.146	6.533	7.120
	150NP	6.733	.146	6.440	7.027
	300NP	10.010	.146	9.716	10.304
	600NP	10.657	.146	10.363	10.950
25%FC	Cont	3.527	.146	3.233	3.820
	Water sprayed	6.763	.146	6.470	7.057
	50NP	8.173	.146	7.880	8.467
	150NP	8.063	.146	7.770	8.357
	300NP	12.050	.146	11.756	12.344
	600NP	12.603	.146	12.310	12.897

Post Hoc Tests

FC_Levels

Homogeneous Subsets

Peroxidase_Sh

Duncan[a,b]

FC_Levels	N	Subset			
		1	2	3	4
FFC	18	3.5450			
75%FC	18		4.5867		
50%FC	18			6.8567	
25%FC	18				8.5300
Sig.		1.000	1.000	1.000	1.000

Means for groups in homogeneous subsets are displayed.
Based on observed means.
The error term is Mean Square(Error) = .064.

a. Uses Harmonic Mean Sample Size = 18.000.

b. Alpha = .05.

NPs

Homogeneous Subsets

Peroxidase_Sh

Duncan[a,b]

NPs	N	Subset				
		1	2	3	4	5
Cont	12	1.9675				
Water sprayed	12		3.6175			
150NP	12			6.1225		
50NP	12			6.1333		
300NP	12				8.5933	
600NP	12					8.8433
Sig.		1.000	1.000	.917	1.000	1.000

Means for groups in homogeneous subsets are displayed.
Based on observed means.
The error term is Mean Square(Error) = .064.

a. Uses Harmonic Mean Sample Size = 12.000.

b. Alpha = .05.

Profile Plots

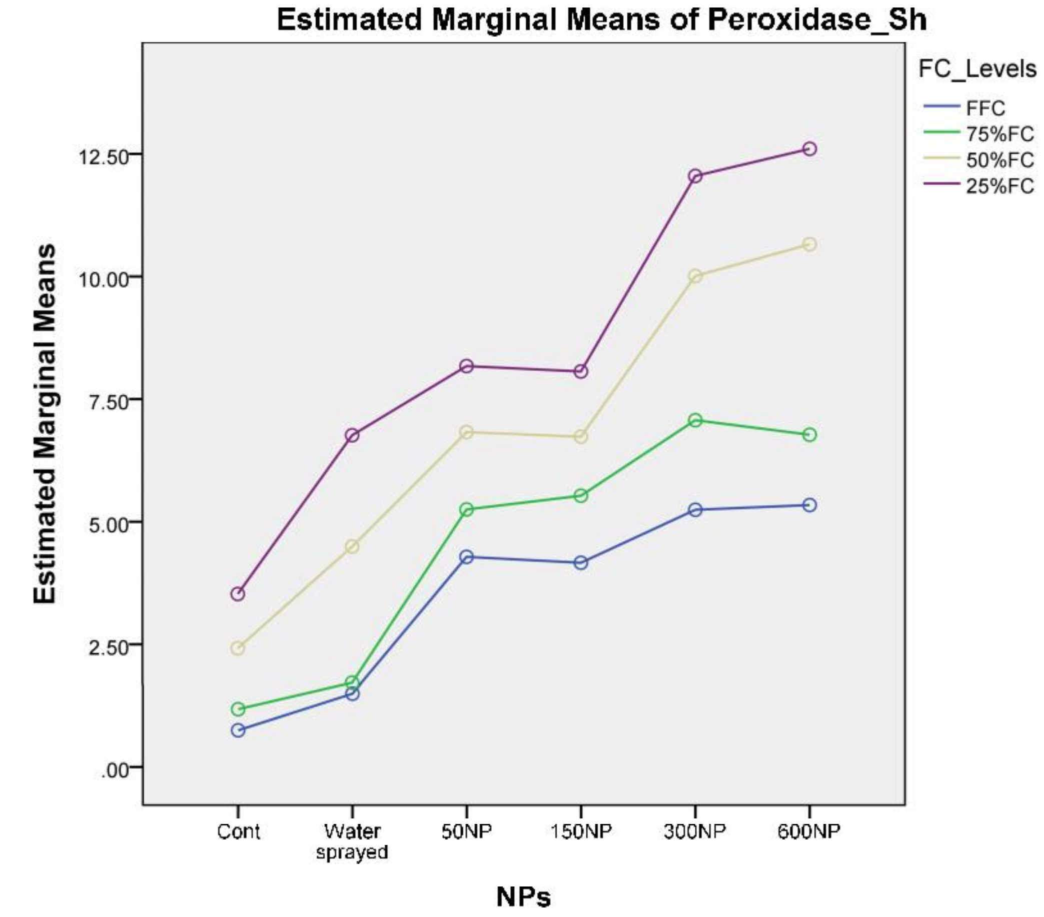


```
UNIANOVA Peroxidase_Sh BY FC_LevOP OPs
  /METHOD=SSTYPE(3)
  /INTERCEPT=EXCLUDE
  /SAVE=SEPRED
  /POSTHOC=FC_LevOP OPs(DUNCAN)
  /PLOT=PROFILE(OPs*FC_LevOP)
  /EMMEANS=TABLES(OVERALL)
  /EMMEANS=TABLES(FC_LevOP)
  /EMMEANS=TABLES(OPs)
  /EMMEANS=TABLES(FC_LevOP*OPs)
  /PRINT=DESCRIPTIVE
  /CRITERIA=ALPHA(.05)
  /DESIGN=FC_LevOP OPs FC_LevOP*OPs.
```

Univariate Analysis of Variance

```
[DataSet1] E:\Amna M Sc\Paper\Two Way ANOVA H.sav
```

Between-Subjects Factors

		Value Label	N
FC_LevOP	1.00	FFC	18
	2.00	75%FC	18
	3.00	50%FC	18
	4.00	25%FC	18
OPs	1.00	Cont	12
	2.00	Water sprayed	12
	3.00	50OPs	12
	4.00	150OPs	12
	5.00	300OPs	12
	6.00	600OPs	12

Descriptive Statistics

Dependent Variable: Peroxidase_Sh

FC_LevOP	OPs	Mean	Std. Deviation	N
FFC	Cont	.7467	.12702	3
	Water sprayed	1.4933	.19858	3
	50OPs	2.2800	.19313	3
	150OPs	2.7000	.32000	3
	300OPs	6.0133	.57353	3
	600OPs	6.6600	.37470	3
	Total	3.3156	2.31151	18
75%FC	Cont	1.1767	.04619	3
	Water sprayed	1.7200	.19313	3
	50OPs	2.6933	.21939	3
	150OPs	4.1667	.20404	3
	300OPs	6.4467	.82585	3
	600OPs	10.0433	1.02963	3
	Total	4.3744	3.19427	18
50%FC	Cont	2.4200	.15133	3
	Water sprayed	4.4933	.07506	3
	50OPs	5.0967	.49903	3
	150OPs	5.5767	.50600	3
	300OPs	9.0600	.76531	3
	600OPs	9.8600	.36373	3
	Total	6.0844	2.69432	18
25%FC	Cont	3.5267	.26558	3
	Water sprayed	6.7633	.21385	3
	50OPs	7.0200	.74478	3
	150OPs	10.2067	.39119	3
	300OPs	10.6867	.37220	3
	600OPs	11.2233	1.17074	3
	Total	8.2378	2.85515	18

Descriptive Statistics

Dependent Variable: Peroxidase_Sh

FC_LevOP	OPs	Mean	Std. Deviation	N
Total	Cont	1.9675	1.14726	12
	Water sprayed	3.6175	2.26804	12
	50OPs	4.2725	2.04170	12
	150OPs	5.6625	2.95602	12
	300OPs	8.0517	2.07896	12
	600OPs	9.4467	1.90104	12
	Total	5.5031	3.30608	72

Tests of Between-Subjects Effects

Dependent Variable: Peroxidase_Sh

Source	Type III Sum of Squares	df	Mean Square	F	Sig.
Model	2944.222[a]	24	122.676	481.024	.000
FC_LevOP	249.761	3	83.254	326.446	.000
OPs	475.712	5	95.142	373.063	.000
FC_LevOP * OPs	38.328	15	2.555	10.019	.000
Error	12.241	48	.255		
Total	2956.463	72			

a. R Squared = .996 (Adjusted R Squared = .994)

Estimated Marginal Means

1. Grand Mean

Dependent Variable: Peroxidase_Sh

Mean	Std. Error	95% Confidence Interval	
		Lower Bound	Upper Bound
5.503	.060	5.383	5.623

2. FC_LevOP

Dependent Variable: Peroxidase_Sh

FC_LevOP	Mean	Std. Error	95% Confidence Interval	
			Lower Bound	Upper Bound
FFC	3.316	.119	3.076	3.555
75%FC	4.374	.119	4.135	4.614
50%FC	6.084	.119	5.845	6.324
25%FC	8.238	.119	7.998	8.477

3. OPs

Dependent Variable: Peroxidase_Sh

OPs	Mean	Std. Error	95% Confidence Interval	
			Lower Bound	Upper Bound
Cont	1.968	.146	1.674	2.261
Water sprayed	3.618	.146	3.324	3.911
50OPs	4.273	.146	3.979	4.566
150OPs	5.663	.146	5.369	5.956
300OPs	8.052	.146	7.759	8.345
600OPs	9.447	.146	9.154	9.740

4. FC_LevOP * OPs

Dependent Variable: Peroxidase_Sh

FC_LevOP	OPs	Mean	Std. Error	95% Confidence Interval	
				Lower Bound	Upper Bound
FFC	Cont	.747	.292	.160	1.333
	Water sprayed	1.493	.292	.907	2.080
	50OPs	2.280	.292	1.694	2.866
	150OPs	2.700	.292	2.114	3.286
	300OPs	6.013	.292	5.427	6.600
	600OPs	6.660	.292	6.074	7.246
75%FC	Cont	1.177	.292	.590	1.763
	Water sprayed	1.720	.292	1.134	2.306
	50OPs	2.693	.292	2.107	3.280
	150OPs	4.167	.292	3.580	4.753
	300OPs	6.447	.292	5.860	7.033
	600OPs	10.043	.292	9.457	10.630
50%FC	Cont	2.420	.292	1.834	3.006
	Water sprayed	4.493	.292	3.907	5.080
	50OPs	5.097	.292	4.510	5.683
	150OPs	5.577	.292	4.990	6.163
	300OPs	9.060	.292	8.474	9.646
	600OPs	9.860	.292	9.274	10.446
25%FC	Cont	3.527	.292	2.940	4.113
	Water sprayed	6.763	.292	6.177	7.350
	50OPs	7.020	.292	6.434	7.606
	150OPs	10.207	.292	9.620	10.793
	300OPs	10.687	.292	10.100	11.273
	600OPs	11.223	.292	10.637	11.810

Post Hoc Tests

FC_LevOP

Homogeneous Subsets

Peroxidase_Sh

Duncan[a,b]

FC_LevOP	N	Subset			
		1	2	3	4
FFC	18	3.3156			
75%FC	18		4.3744		
50%FC	18			6.0844	
25%FC	18				8.2378
Sig.		1.000	1.000	1.000	1.000

Means for groups in homogeneous subsets are displayed.
Based on observed means.
The error term is Mean Square(Error) = .255.

a. Uses Harmonic Mean Sample Size = 18.000.

b. Alpha = .05.

OPs

Homogeneous Subsets

Peroxidase_Sh

Duncan[a,b]

OPs	N	Subset					
		1	2	3	4	5	6
Cont	12	1.9675					
Water sprayed	12		3.6175				
50OPs	12			4.2725			
150OPs	12				5.6625		
300OPs	12					8.0517	
600OPs	12						9.4467
Sig.		1.000	1.000	1.000	1.000	1.000	1.000

Means for groups in homogeneous subsets are displayed.
Based on observed means.
The error term is Mean Square(Error) = .255.

a. Uses Harmonic Mean Sample Size = 12.000.

b. Alpha = .05.

Profile Plots

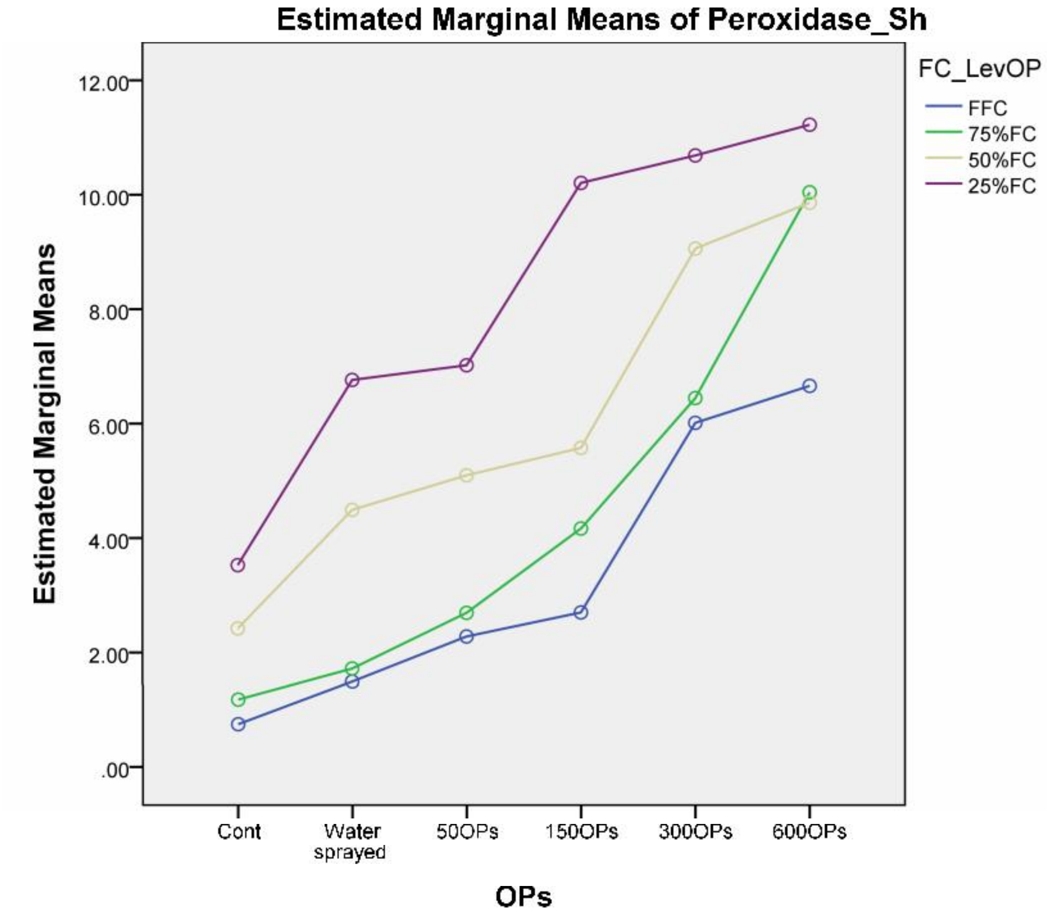


```
UNIANOVA Peroxidase_Ro BY FC_Levels NPs
  /METHOD=SSTYPE(3)
  /INTERCEPT=EXCLUDE
  /SAVE=SEPRED
  /POSTHOC=FC_Levels NPs(DUNCAN)
  /PLOT=PROFILE(NPs*FC_Levels)
  /EMMEANS=TABLES(OVERALL)
  /EMMEANS=TABLES(FC_Levels)
  /EMMEANS=TABLES(NPs)
  /EMMEANS=TABLES(FC_Levels*NPs)
  /PRINT=DESCRIPTIVE
  /CRITERIA=ALPHA(.05)
  /DESIGN=FC_Levels NPs FC_Levels*NPs.
```

Univariate Analysis of Variance

[DataSet1] E:\Amna M Sc\Paper\Two Way ANOVA H.sav

Between-Subjects Factors

		Value Label	N
FC_Levels	1	FFC	18
	2	75%FC	18
	3	50%FC	18
	4	25%FC	18
NPs	1	Cont	12
	2	Water sprayed	12
	3	50NP	12
	4	150NP	12
	5	300NP	12
	6	600NP	12

Descriptive Statistics

Dependent Variable: Peroxidase_Ro

FC_Levels	NPs	Mean	Std. Deviation	N
FFC	Cont	21.5497	.42254	3
	Water sprayed	20.3743	.82089	3
	50NP	20.6256	1.96907	3
	150NP	21.2557	.24381	3
	300NP	20.5320	3.00683	3
	600NP	24.3405	.59367	3
	Total	21.4463	1.90373	18
75%FC	Cont	30.7008	2.08258	3
	Water sprayed	22.9863	1.06091	3
	50NP	19.9573	4.23023	3
	150NP	17.9890	1.29821	3
	300NP	17.3517	2.06992	3
	600NP	17.5388	1.98986	3
	Total	21.0873	5.24077	18
50%FC	Cont	37.5321	2.24699	3
	Water sprayed	19.3545	.39672	3
	50NP	19.9275	1.85742	3
	150NP	26.5263	4.12948	3
	300NP	32.2965	2.42612	3
	600NP	59.6985	5.30251	3
	Total	32.5559	14.37808	18
25%FC	Cont	44.8942	5.05966	3
	Water sprayed	24.6911	2.54559	3
	50NP	24.2351	1.38824	3
	150NP	28.5213	1.74082	3
	300NP	49.2271	2.25448	3
	600NP	55.4911	.51953	3
	Total	37.8433	13.04341	18

Descriptive Statistics

Dependent Variable: Peroxidase_Ro

FC_Levels	NPs	Mean	Std. Deviation	N
Total	Cont	33.6692	9.34287	12
	Water sprayed	21.8516	2.52581	12
	50NP	21.1864	2.89848	12
	150NP	23.5731	4.79519	12
	300NP	29.8518	13.21862	12
	600NP	39.2672	19.52188	12
	Total	28.2332	12.26591	72

Tests of Between-Subjects Effects

Dependent Variable: Peroxidase_Ro

Source	Type III Sum of Squares	df	Mean Square	F	Sig.
Model	67775.216[a]	24	2823.967	453.129	.000
FC_Levels	3746.986	3	1248.995	200.412	.000
NPs	3192.234	5	638.447	102.444	.000
FC_Levels * NPs	3443.766	15	229.584	36.839	.000
Error	299.143	48	6.232		
Total	68074.359	72			

a. R Squared = .996 (Adjusted R Squared = .993)

Estimated Marginal Means

1. Grand Mean

Dependent Variable: Peroxidase_Ro

Mean	Std. Error	95% Confidence Interval	
		Lower Bound	Upper Bound
28.233	.294	27.642	28.825

2. FC_Levels

Dependent Variable: Peroxidase_Ro

FC_Levels	Mean	Std. Error	95% Confidence Interval	
			Lower Bound	Upper Bound
FFC	21.446	.588	20.263	22.629
75%FC	21.087	.588	19.904	22.270
50%FC	32.556	.588	31.373	33.739
25%FC	37.843	.588	36.660	39.026

3. NPs

Dependent Variable: Peroxidase_Ro

NPs	Mean	Std. Error	95% Confidence Interval	
			Lower Bound	Upper Bound
Cont	33.669	.721	32.220	35.118
Water sprayed	21.852	.721	20.403	23.301
50NP	21.186	.721	19.737	22.635
150NP	23.573	.721	22.124	25.022
300NP	29.852	.721	28.403	31.301
600NP	39.267	.721	37.818	40.716

4. FC_Levels * NPs

Dependent Variable: Peroxidase_Ro

FC_Levels	NPs	Mean	Std. Error	95% Confidence Interval	
				Lower Bound	Upper Bound
FFC	Cont	21.550	1.441	18.652	24.448
	Water sprayed	20.374	1.441	17.476	23.272
	50NP	20.626	1.441	17.728	23.524
	150NP	21.256	1.441	18.358	24.154
	300NP	20.532	1.441	17.634	23.430
	600NP	24.341	1.441	21.443	27.238
75%FC	Cont	30.701	1.441	27.803	33.599
	Water sprayed	22.986	1.441	20.088	25.884
	50NP	19.957	1.441	17.059	22.855
	150NP	17.989	1.441	15.091	20.887
	300NP	17.352	1.441	14.454	20.250
	600NP	17.539	1.441	14.641	20.437
50%FC	Cont	37.532	1.441	34.634	40.430
	Water sprayed	19.355	1.441	16.457	22.252
	50NP	19.927	1.441	17.030	22.825
	150NP	26.526	1.441	23.628	29.424
	300NP	32.297	1.441	29.399	35.194
	600NP	59.698	1.441	56.801	62.596
25%FC	Cont	44.894	1.441	41.996	47.792
	Water sprayed	24.691	1.441	21.793	27.589
	50NP	24.235	1.441	21.337	27.133
	150NP	28.521	1.441	25.623	31.419
	300NP	49.227	1.441	46.329	52.125
	600NP	55.491	1.441	52.593	58.389

Post Hoc Tests

FC_Levels

Homogeneous Subsets

Peroxidase_Ro

Duncan[a,b]

FC_Levels	N	Subset 1	2	3
75%FC	18	21.0873		
FFC	18	21.4463		
50%FC	18		32.5559	
25%FC	18			37.8433
Sig.		.668	1.000	1.000

Means for groups in homogeneous subsets are displayed.
Based on observed means.
The error term is Mean Square(Error) = 6.232.

a. Uses Harmonic Mean Sample Size = 18.000.

b. Alpha = .05.

NPs

Homogeneous Subsets

Peroxidase_Ro

Duncan[a,b]

NPs	N	Subset 1	2	3	4	5
50NP	12	21.1864				
Water sprayed	12	21.8516	21.8516			
150NP	12		23.5731			
300NP	12			29.8518		
Cont	12				33.6692	
600NP	12					39.2672
Sig.		.517	.098	1.000	1.000	1.000

Means for groups in homogeneous subsets are displayed.
Based on observed means.
The error term is Mean Square(Error) = 6.232.

a. Uses Harmonic Mean Sample Size = 12.000.

b. Alpha = .05.

Profile Plots

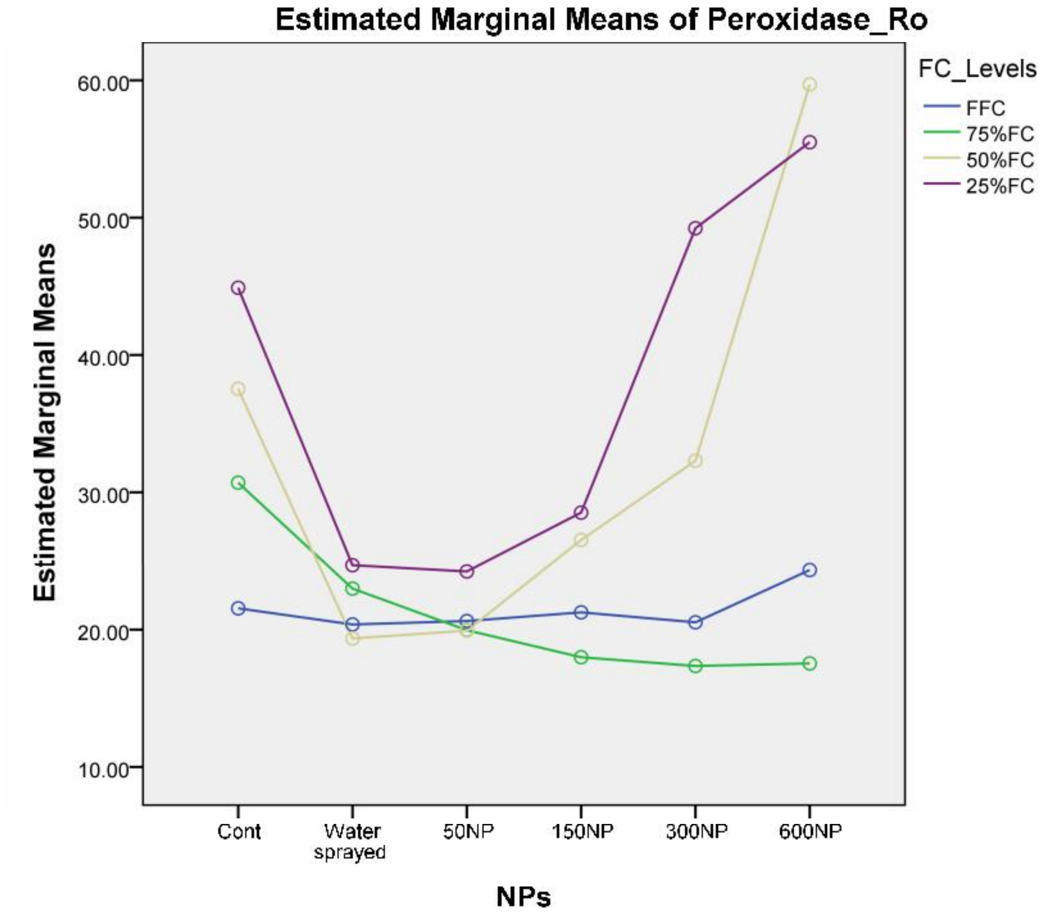


```
UNIANOVA Peroxidase_Ro BY FC_LevOP OPs
  /METHOD=SSTYPE(3)
  /INTERCEPT=EXCLUDE
  /SAVE=SEPRED
  /POSTHOC=FC_LevOP OPs(DUNCAN)
  /PLOT=PROFILE(OPs*FC_LevOP)
  /EMMEANS=TABLES(OVERALL)
  /EMMEANS=TABLES(FC_LevOP)
  /EMMEANS=TABLES(OPs)
  /EMMEANS=TABLES(FC_LevOP*OPs)
  /PRINT=DESCRIPTIVE
  /CRITERIA=ALPHA(.05)
  /DESIGN=FC_LevOP OPs FC_LevOP*OPs.
```

Univariate Analysis of Variance

```
[DataSet1] E:\Amna M Sc\Paper\Two Way ANOVA H.sav
```

Between-Subjects Factors

		Value Label	N
FC_LevOP	1.00	FFC	18
	2.00	75%FC	18
	3.00	50%FC	18
	4.00	25%FC	18
OPs	1.00	Cont	12
	2.00	Water sprayed	12
	3.00	50OPs	12
	4.00	150OPs	12
	5.00	300OPs	12
	6.00	600OPs	12

Descriptive Statistics

Dependent Variable: Peroxidase_Ro

FC_LevOP	OPs	Mean	Std. Deviation	N
FFC	Cont	21.5497	.42254	3
	Water sprayed	20.3743	.82089	3
	50OPs	13.6714	1.03346	3
	150OPs	15.9384	.65055	3
	300OPs	15.5386	1.75534	3
	600OPs	22.9317	2.58907	3
	Total	18.3340	3.73430	18
75%FC	Cont	30.7008	2.08258	3
	Water sprayed	22.9863	1.06091	3
	50OPs	20.0594	2.48953	3
	150OPs	19.3806	3.13431	3
	300OPs	20.0120	1.48453	3
	600OPs	25.0862	2.84500	3
	Total	23.0376	4.51485	18
50%FC	Cont	37.5321	2.24699	3
	Water sprayed	19.3545	.39672	3
	50OPs	25.2409	1.17492	3
	150OPs	23.9466	.88820	3
	300OPs	25.0822	.54992	3
	600OPs	33.8068	2.26708	3
	Total	27.4939	6.49282	18
25%FC	Cont	44.8942	5.05966	3
	Water sprayed	24.6911	2.54559	3
	50OPs	30.5948	1.61153	3
	150OPs	29.8295	1.53312	3
	300OPs	35.1471	5.84100	3
	600OPs	35.5562	2.31248	3
	Total	33.4522	7.11989	18

Descriptive Statistics

Dependent Variable: Peroxidase_Ro

FC_LevOP	OPs	Mean	Std. Deviation	N
Total	Cont	33.6692	9.34287	12
	Water sprayed	21.8516	2.52581	12
	50OPs	22.3917	6.69575	12
	150OPs	22.2738	5.65653	12
	300OPs	23.9450	8.08006	12
	600OPs	29.3452	6.05904	12
	Total	25.5794	7.87658	72

Tests of Between-Subjects Effects

Dependent Variable: Peroxidase_Ro

Source	Type III Sum of Squares	df	Mean Square	F	Sig.
Model	51248.456[a]	24	2135.352	384.688	.000
FC_LevOP	2242.841	3	747.614	134.684	.000
OPs	1407.399	5	281.480	50.709	.000
FC_LevOP * OPs	488.193	15	32.546	5.863	.000
Error	266.441	48	5.551		
Total	51514.898	72			

a. R Squared = .995 (Adjusted R Squared = .992)

Estimated Marginal Means

1. Grand Mean

Dependent Variable: Peroxidase_Ro

Mean	Std. Error	95% Confidence Interval	
		Lower Bound	Upper Bound
25.579	.278	25.021	26.138

2. FC_LevOP

Dependent Variable: Peroxidase_Ro

FC_LevOP	Mean	Std. Error	95% Confidence Interval	
			Lower Bound	Upper Bound
FFC	18.334	.555	17.217	19.451
75%FC	23.038	.555	21.921	24.154
50%FC	27.494	.555	26.377	28.610
25%FC	33.452	.555	32.336	34.569

3. OPs

Dependent Variable: Peroxidase_Ro

OPs	Mean	Std. Error	95% Confidence Interval	
			Lower Bound	Upper Bound
Cont	33.669	.680	32.302	35.037
Water sprayed	21.852	.680	20.484	23.219
50OPs	22.392	.680	21.024	23.759
150OPs	22.274	.680	20.906	23.641
300OPs	23.945	.680	22.578	25.312
600OPs	29.345	.680	27.978	30.713

4. FC_LevOP * OPs

Dependent Variable: Peroxidase_Ro

FC_LevOP	OPs	Mean	Std. Error	95% Confidence Interval	
				Lower Bound	Upper Bound
FFC	Cont	21.550	1.360	18.815	24.285
	Water sprayed	20.374	1.360	17.639	23.109
	50OPs	13.671	1.360	10.936	16.406
	150OPs	15.938	1.360	13.203	18.673
	300OPs	15.539	1.360	12.804	18.274
	600OPs	22.932	1.360	20.197	25.667
75%FC	Cont	30.701	1.360	27.966	33.436
	Water sprayed	22.986	1.360	20.251	25.721
	50OPs	20.059	1.360	17.324	22.794
	150OPs	19.381	1.360	16.646	22.116
	300OPs	20.012	1.360	17.277	22.747
	600OPs	25.086	1.360	22.351	27.821
50%FC	Cont	37.532	1.360	34.797	40.267
	Water sprayed	19.355	1.360	16.620	22.090
	50OPs	25.241	1.360	22.506	27.976
	150OPs	23.947	1.360	21.212	26.682
	300OPs	25.082	1.360	22.347	27.817
	600OPs	33.807	1.360	31.072	36.542
25%FC	Cont	44.894	1.360	42.159	47.629
	Water sprayed	24.691	1.360	21.956	27.426
	50OPs	30.595	1.360	27.860	33.330
	150OPs	29.830	1.360	27.095	32.565
	300OPs	35.147	1.360	32.412	37.882
	600OPs	35.556	1.360	32.821	38.291

Post Hoc Tests

FC_LevOP

Homogeneous Subsets

Peroxidase_Ro

Duncan[a,b]

FC_LevOP	N	Subset			
		1	2	3	4
FFC	18	18.3340			
75%FC	18		23.0376		
50%FC	18			27.4939	
25%FC	18				33.4522
Sig.		1.000	1.000	1.000	1.000

Means for groups in homogeneous subsets are displayed.
Based on observed means.
The error term is Mean Square(Error) = 5.551.

a. Uses Harmonic Mean Sample Size = 18.000.

b. Alpha = .05.

OPs

Homogeneous Subsets

Peroxidase_Ro

Duncan[a,b]

OPs	N	Subset		
		1	2	3
Water sprayed	12	21.8516		
150OPs	12	22.2738		
50OPs	12	22.3917		
300OPs	12	23.9450		
600OPs	12		29.3452	
Cont	12			33.6692
Sig.		.051	1.000	1.000

Means for groups in homogeneous subsets are displayed.
Based on observed means.
The error term is Mean Square(Error) = 5.551.

a. Uses Harmonic Mean Sample Size = 12.000.

b. Alpha = .05.

Profile Plots

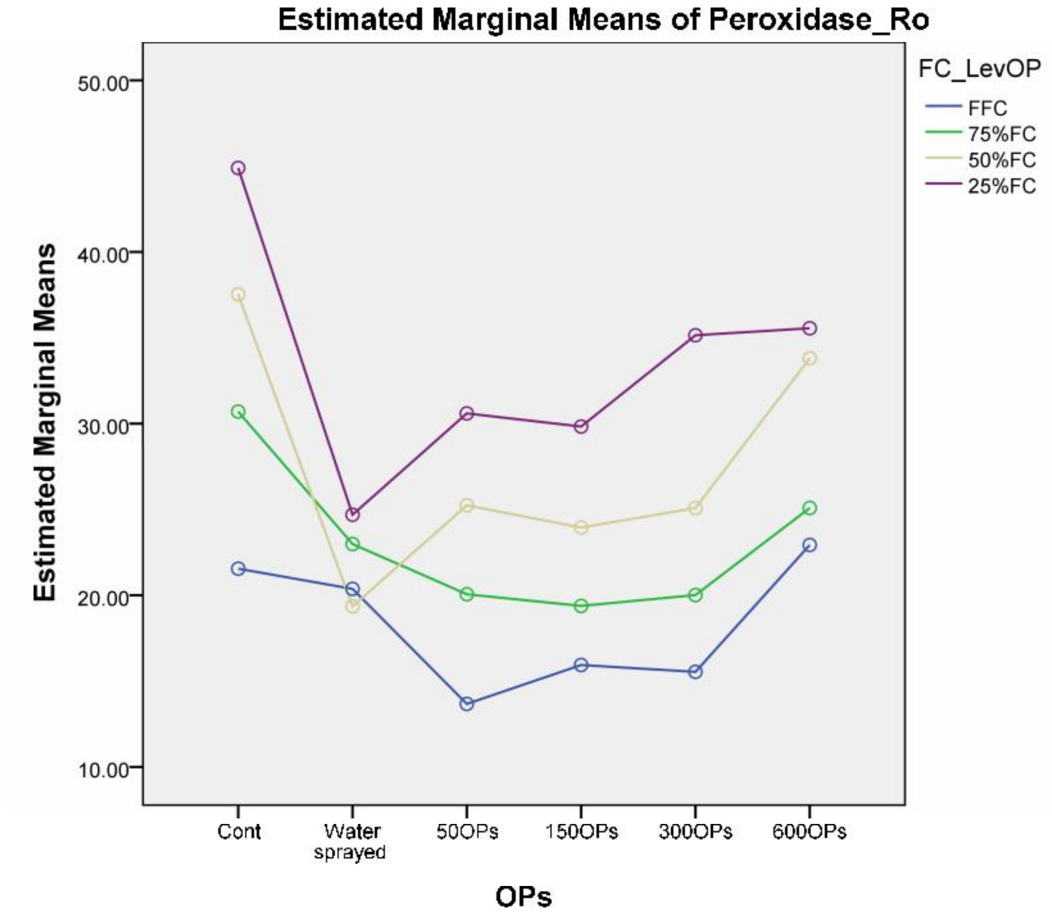


```
UNIANOVA Ascorbate.Per_Sh BY FC_Levels NPs
  /METHOD=SSTYPE(3)
  /INTERCEPT=EXCLUDE
  /SAVE=SEPRED
  /POSTHOC=FC_Levels NPs(DUNCAN)
  /PLOT=PROFILE(NPs*FC_Levels)
  /EMMEANS=TABLES(OVERALL)
  /EMMEANS=TABLES(FC_Levels)
  /EMMEANS=TABLES(NPs)
  /EMMEANS=TABLES(FC_Levels*NPs)
  /PRINT=DESCRIPTIVE
  /CRITERIA=ALPHA(.05)
  /DESIGN=FC_Levels NPs FC_Levels*NPs.
```

Univariate Analysis of Variance

[DataSet1] E:\Amna M Sc\Paper\Two Way ANOVA H.sav

Between-Subjects Factors

		Value Label	N
FC_Levels	1	FFC	18
	2	75%FC	18
	3	50%FC	18
	4	25%FC	18
NPs	1	Cont	12
	2	Water sprayed	12
	3	50NP	12
	4	150NP	12
	5	300NP	12
	6	600NP	12

Descriptive Statistics

Dependent Variable: Ascorbate.Per_Sh

FC_Levels	NPs	Mean	Std. Deviation	N
FFC	Cont	82.6813	7.18950	3
	Water sprayed	101.6780	29.12129	3
	50NP	70.5403	5.77252	3
	150NP	61.2537	5.61420	3
	300NP	66.4290	10.67090	3
	600NP	54.3110	7.11118	3
	Total	72.8156	19.72657	18
75%FC	Cont	97.8413	12.75098	3
	Water sprayed	139.5470	6.06700	3
	50NP	96.9950	21.42408	3
	150NP	76.9907	4.76256	3
	300NP	96.3050	12.13342	3
	600NP	61.7190	22.00917	3
	Total	94.8997	27.56347	18
50%FC	Cont	164.7553	14.02150	3
	Water sprayed	165.5713	21.24245	3
	50NP	123.0010	13.07929	3
	150NP	289.0647	10.34274	3
	300NP	267.7100	18.25300	3
	600NP	201.7110	6.50700	3
	Total	201.9689	61.91188	18
25%FC	Cont	224.1927	21.01315	3
	Water sprayed	181.0537	6.70550	3
	50NP	164.6913	19.77903	3
	150NP	359.9317	56.82484	3
	300NP	300.0473	29.95671	3
	600NP	231.3440	23.77029	3
	Total	243.5434	74.07980	18

Descriptive Statistics

Dependent Variable: Ascorbate.Per_Sh

FC_Levels	NPs	Mean	Std. Deviation	N
Total	Cont	142.3677	60.24921	12
	Water sprayed	146.9625	35.16796	12
	50NP	113.8069	38.84042	12
	150NP	196.8102	138.28030	12
	300NP	182.6228	108.26049	12
	600NP	137.2713	84.77929	12
	Total	153.3069	87.62936	72

Tests of Between-Subjects Effects

Dependent Variable: Ascorbate.Per_Sh

Source	Type III Sum of Squares	df	Mean Square	F	Sig.
Model	2218793.68[a]	24	92449.737	238.264	.000
FC_Levels	367215.922	3	122405.307	315.467	.000
NPs	56751.206	5	11350.241	29.252	.000
FC_Levels * NPs	102610.397	15	6840.693	17.630	.000
Error	18624.653	48	388.014		
Total	2237418.336	72			

a. R Squared = .992 (Adjusted R Squared = .988)

Estimated Marginal Means

1. Grand Mean

Dependent Variable: Ascorbate.Per_Sh

Mean	Std. Error	95% Confidence Interval	
		Lower Bound	Upper Bound
153.307	2.321	148.639	157.974

2. FC_Levels

Dependent Variable: Ascorbate.Per_Sh

FC_Levels	Mean	Std. Error	95% Confidence Interval	
			Lower Bound	Upper Bound
FFC	72.816	4.643	63.480	82.151
75%FC	94.900	4.643	85.565	104.235
50%FC	201.969	4.643	192.634	211.304
25%FC	243.543	4.643	234.208	252.879

3. NPs

Dependent Variable: Ascorbate.Per_Sh

NPs	Mean	Std. Error	95% Confidence Interval	
			Lower Bound	Upper Bound
Cont	142.368	5.686	130.935	153.801
Water sprayed	146.963	5.686	135.529	158.396
50NP	113.807	5.686	102.374	125.240
150NP	196.810	5.686	185.377	208.243
300NP	182.623	5.686	171.190	194.056
600NP	137.271	5.686	125.838	148.704

4. FC_Levels * NPs

Dependent Variable: Ascorbate.Per_Sh

FC_Levels	NPs	Mean	Std. Error	95% Confidence Interval	
				Lower Bound	Upper Bound
FFC	Cont	82.681	11.373	59.815	105.548
	Water sprayed	101.678	11.373	78.812	124.544
	50NP	70.540	11.373	47.674	93.407
	150NP	61.254	11.373	38.387	84.120
	300NP	66.429	11.373	43.563	89.295
	600NP	54.311	11.373	31.445	77.177
75%FC	Cont	97.841	11.373	74.975	120.708
	Water sprayed	139.547	11.373	116.681	162.413
	50NP	96.995	11.373	74.129	119.861
	150NP	76.991	11.373	54.124	99.857
	300NP	96.305	11.373	73.439	119.171
	600NP	61.719	11.373	38.853	84.585
50%FC	Cont	164.755	11.373	141.889	187.622
	Water sprayed	165.571	11.373	142.705	188.438
	50NP	123.001	11.373	100.135	145.867
	150NP	289.065	11.373	266.198	311.931
	300NP	267.710	11.373	244.844	290.576
	600NP	201.711	11.373	178.845	224.577
25%FC	Cont	224.193	11.373	201.326	247.059
	Water sprayed	181.054	11.373	158.187	203.920
	50NP	164.691	11.373	141.825	187.558
	150NP	359.932	11.373	337.065	382.798
	300NP	300.047	11.373	277.181	322.914
	600NP	231.344	11.373	208.478	254.210

Post Hoc Tests

FC_Levels

Homogeneous Subsets

Ascorbate.Per_Sh

Duncan[a,b]

FC_Levels	N	Subset 1	2	3	4
FFC	18	72.8156			
75%FC	18		94.8997		
50%FC	18			201.9689	
25%FC	18				243.5434
Sig.		1.000	1.000	1.000	1.000

Means for groups in homogeneous subsets are displayed.
Based on observed means.
The error term is Mean Square(Error) = 388.014.

a. Uses Harmonic Mean Sample Size = 18.000.

b. Alpha = .05.

NPs

Homogeneous Subsets

Ascorbate.Per_Sh

Duncan[a,b]

NPs	N	Subset 1	2	3
50NP	12	113.8069		
600NP	12		137.2713	
Cont	12		142.3677	
Water sprayed	12		146.9625	
300NP	12			182.6228
150NP	12			196.8102
Sig.		1.000	.262	.084

Means for groups in homogeneous subsets are displayed.
Based on observed means.
The error term is Mean Square(Error) = 388.014.

a. Uses Harmonic Mean Sample Size = 12.000.

b. Alpha = .05.

Profile Plots

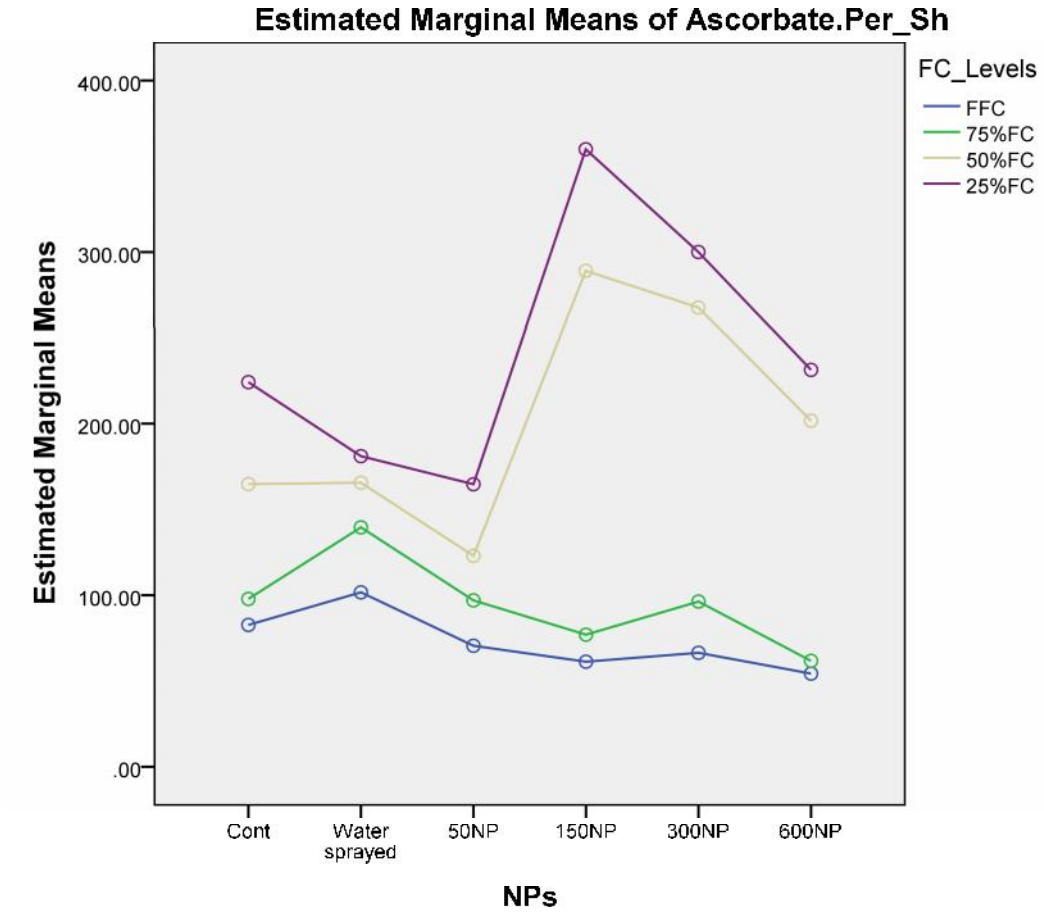


```
UNIANOVA Ascorbate.Per_Sh BY FC_LevOP OPs
  /METHOD=SSTYPE(3)
  /INTERCEPT=EXCLUDE
  /SAVE=SEPRED
  /POSTHOC=FC_LevOP OPs(DUNCAN)
  /PLOT=PROFILE(OPs*FC_LevOP)
  /EMMEANS=TABLES(OVERALL)
  /EMMEANS=TABLES(FC_LevOP)
  /EMMEANS=TABLES(OPs)
  /EMMEANS=TABLES(FC_LevOP*OPs)
  /PRINT=DESCRIPTIVE
  /CRITERIA=ALPHA(.05)
  /DESIGN=FC_LevOP OPs FC_LevOP*OPs.
```

Univariate Analysis of Variance

```
[DataSet1] E:\Amna M Sc\Paper\Two Way ANOVA H.sav
```

Between-Subjects Factors

		Value Label	N
FC_LevOP	1.00	FFC	18
	2.00	75%FC	18
	3.00	50%FC	18
	4.00	25%FC	18
OPs	1.00	Cont	12
	2.00	Water sprayed	12
	3.00	50OPs	12
	4.00	150OPs	12
	5.00	300OPs	12
	6.00	600OPs	12

Descriptive Statistics

Dependent Variable: Ascorbate.Per_Sh

FC_LevOP	OPs	Mean	Std. Deviation	N
FFC	Cont	82.6813	7.18950	3
	Water sprayed	101.6780	29.12129	3
	50OPs	109.9727	12.48695	3
	150OPs	258.7250	13.10949	3
	300OPs	334.1733	54.34385	3
	600OPs	233.5490	53.51270	3
	Total	186.7966	100.96247	18
75%FC	Cont	97.8413	12.75098	3
	Water sprayed	139.5470	6.06700	3
	50OPs	148.3910	18.54900	3
	150OPs	239.4090	13.34916	3
	300OPs	324.1387	7.90550	3
	600OPs	222.3957	18.19894	3
	Total	195.2871	78.39920	18
50%FC	Cont	164.7553	14.02150	3
	Water sprayed	165.5713	21.24245	3
	50OPs	170.8137	25.82461	3
	150OPs	238.1027	16.65810	3
	300OPs	445.9057	19.30833	3
	600OPs	148.4397	40.90296	3
	Total	222.2647	108.98759	18
25%FC	Cont	224.1927	21.01315	3
	Water sprayed	181.0537	6.70550	3
	50OPs	177.1050	9.02225	3
	150OPs	304.3017	32.94130	3
	300OPs	408.4890	10.88362	3
	600OPs	246.0917	12.30450	3
	Total	256.8723	83.82184	18

Descriptive Statistics

Dependent Variable: Ascorbate.Per_Sh

FC_LevOP	OPs	Mean	Std. Deviation	N
Total	Cont	142.3677	60.24921	12
	Water sprayed	146.9625	35.16796	12
	50OPs	151.5706	31.31221	12
	150OPs	260.1346	33.06795	12
	300OPs	378.1767	58.85374	12
	600OPs	212.6190	49.87046	12
	Total	215.3052	95.89954	72

Tests of Between-Subjects Effects

Dependent Variable: Ascorbate.Per_Sh

Source	Type III Sum of Squares	df	Mean Square	F	Sig.
Model	3963180.46[a]	24	165132.519	288.846	.000
FC_LevOP	53815.025	3	17938.342	31.377	.000
OPs	511160.564	5	102232.113	178.822	.000
FC_LevOP * OPs	60550.206	15	4036.680	7.061	.000
Error	27441.508	48	571.698		
Total	3990621.968	72			

a. R Squared = .993 (Adjusted R Squared = .990)

Estimated Marginal Means

1. Grand Mean

Dependent Variable: Ascorbate.Per_Sh

Mean	Std. Error	95% Confidence Interval	
		Lower Bound	Upper Bound
215.305	2.818	209.640	220.971

2. FC_LevOP

Dependent Variable: Ascorbate.Per_Sh

FC_LevOP	Mean	Std. Error	95% Confidence Interval	
			Lower Bound	Upper Bound
FFC	186.797	5.636	175.465	198.128
75%FC	195.287	5.636	183.956	206.618
50%FC	222.265	5.636	210.933	233.596
25%FC	256.872	5.636	245.541	268.204

3. OPs

Dependent Variable: Ascorbate.Per_Sh

OPs	Mean	Std. Error	95% Confidence Interval	
			Lower Bound	Upper Bound
Cont	142.368	6.902	128.490	156.246
Water sprayed	146.963	6.902	133.085	160.840
50OPs	151.571	6.902	137.693	165.449
150OPs	260.135	6.902	246.257	274.013
300OPs	378.177	6.902	364.299	392.055
600OPs	212.619	6.902	198.741	226.497

4. FC_LevOP * OPs

Dependent Variable: Ascorbate.Per_Sh

FC_LevOP	OPs	Mean	Std. Error	95% Confidence Interval	
				Lower Bound	Upper Bound
FFC	Cont	82.681	13.805	54.925	110.437
	Water sprayed	101.678	13.805	73.922	129.434
	50OPs	109.973	13.805	82.217	137.729
	150OPs	258.725	13.805	230.969	286.481
	300OPs	334.173	13.805	306.417	361.929
	600OPs	233.549	13.805	205.793	261.305
75%FC	Cont	97.841	13.805	70.085	125.597
	Water sprayed	139.547	13.805	111.791	167.303
	50OPs	148.391	13.805	120.635	176.147
	150OPs	239.409	13.805	211.653	267.165
	300OPs	324.139	13.805	296.383	351.895
	600OPs	222.396	13.805	194.640	250.152
50%FC	Cont	164.755	13.805	136.999	192.511
	Water sprayed	165.571	13.805	137.815	193.327
	50OPs	170.814	13.805	143.058	198.570
	150OPs	238.103	13.805	210.347	265.859
	300OPs	445.906	13.805	418.150	473.662
	600OPs	148.440	13.805	120.684	176.196
25%FC	Cont	224.193	13.805	196.437	251.949
	Water sprayed	181.054	13.805	153.298	208.810
	50OPs	177.105	13.805	149.349	204.861
	150OPs	304.302	13.805	276.546	332.058
	300OPs	408.489	13.805	380.733	436.245
	600OPs	246.092	13.805	218.336	273.848

Post Hoc Tests

FC_LevOP

Homogeneous Subsets

Ascorbate.Per_Sh

Duncan[a,b]

FC_LevOP	N	Subset 1	Subset 2	Subset 3
FFC	18	186.7966		
75%FC	18	195.2871		
50%FC	18		222.2647	
25%FC	18			256.8723
Sig.		.292	1.000	1.000

Means for groups in homogeneous subsets are displayed.
Based on observed means.
The error term is Mean Square(Error) = 571.698.

a. Uses Harmonic Mean Sample Size = 18.000.

b. Alpha = .05.

OPs

Homogeneous Subsets

Ascorbate.Per_Sh

Duncan[a,b]

OPs	N	Subset 1	Subset 2	Subset 3	Subset 4
Cont	12	142.3677			
Water sprayed	12	146.9625			
50OPs	12	151.5706			
600OPs	12		212.6190		
150OPs	12			260.1346	
300OPs	12				378.1767
Sig.		.380	1.000	1.000	1.000

Means for groups in homogeneous subsets are displayed.
Based on observed means.
The error term is Mean Square(Error) = 571.698.

a. Uses Harmonic Mean Sample Size = 12.000.

b. Alpha = .05.

Profile Plots

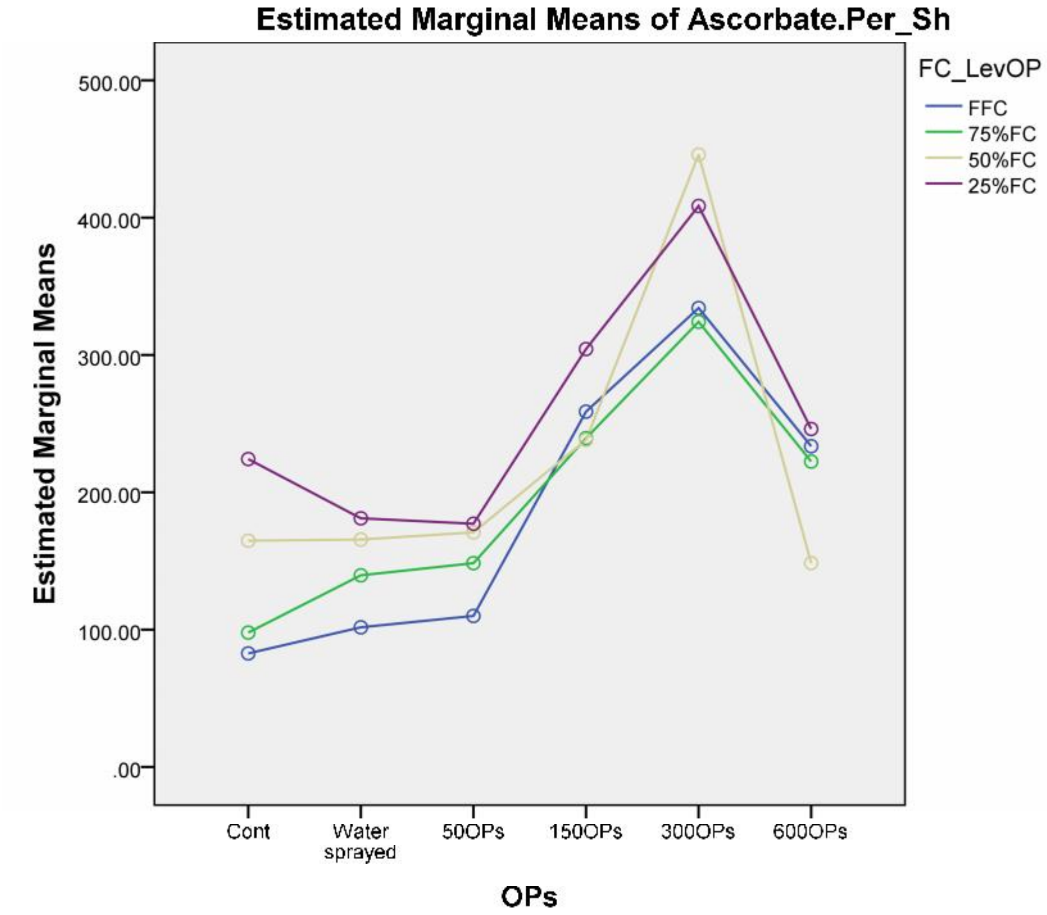


```
UNIANOVA Ascorbate.Per_Ro BY FC_Levels NPs
  /METHOD=SSTYPE(3)
  /INTERCEPT=EXCLUDE
  /SAVE=SEPRED
  /POSTHOC=FC_Levels NPs(DUNCAN)
  /PLOT=PROFILE(NPs*FC_Levels)
  /EMMEANS=TABLES(OVERALL)
  /EMMEANS=TABLES(FC_Levels)
  /EMMEANS=TABLES(NPs)
  /EMMEANS=TABLES(FC_Levels*NPs)
  /PRINT=DESCRIPTIVE
  /CRITERIA=ALPHA(.05)
  /DESIGN=FC_Levels NPs FC_Levels*NPs.
```

Univariate Analysis of Variance

```
[DataSet1] E:\Amna M Sc\Paper\Two Way ANOVA H.sav
```

Between-Subjects Factors

		Value Label	N
FC_Levels	1	FFC	18
	2	75%FC	18
	3	50%FC	18
	4	25%FC	18
NPs	1	Cont	12
	2	Water sprayed	12
	3	50NP	12
	4	150NP	12
	5	300NP	12
	6	600NP	12

Descriptive Statistics

Dependent Variable: Ascorbate.Per_Ro

FC_Levels	NPs	Mean	Std. Deviation	N
FFC	Cont	217.3833	42.41875	3
	Water sprayed	171.1733	38.03500	3
	50NP	360.3167	59.13573	3
	150NP	366.6200	40.74000	3
	300NP	386.9267	79.51062	3
	600NP	477.2233	146.93301	3
	Total	329.9406	125.52940	18
75%FC	Cont	240.8800	64.93510	3
	Water sprayed	193.3033	9.84382	3
	50NP	332.6500	32.43482	3
	150NP	321.0900	31.30881	3
	300NP	409.6667	20.27077	3
	600NP	527.4500	68.95294	3
	Total	337.5067	118.35940	18
50%FC	Cont	337.3867	40.51883	3
	Water sprayed	243.0367	25.31109	3
	50NP	444.6967	86.96215	3
	150NP	464.5400	34.89959	3
	300NP	474.4200	41.08425	3
	600NP	654.9000	51.74710	3
	Total	436.4967	137.75111	18
25%FC	Cont	422.6767	58.85663	3
	Water sprayed	255.6700	30.83091	3
	50NP	496.7767	33.47790	3
	150NP	496.2633	69.10135	3
	300NP	545.6967	75.99165	3
	600NP	752.2767	90.34430	3
	Total	494.8933	161.30376	18

Descriptive Statistics

Dependent Variable: Ascorbate.Per_Ro

FC_Levels	NPs	Mean	Std. Deviation	N
Total	Cont	304.5817	96.43450	12
	Water sprayed	215.7958	43.43940	12
	50NP	408.6100	84.20635	12
	150NP	412.1283	84.11028	12
	300NP	454.1775	82.16347	12
	600NP	602.9625	139.44091	12
	Total	399.7093	150.84911	72

Tests of Between-Subjects Effects

Dependent Variable: Ascorbate.Per_Ro

Source	Type III Sum of Squares	df	Mean Square	F	Sig.
Model	12943897.5[a]	24	539329.065	147.929	.000
FC_Levels	344702.805	3	114900.935	31.515	.000
NPs	1048626.394	5	209725.279	57.524	.000
FC_Levels * NPs	47306.266	15	3153.751	.865	.605
Error	175001.842	48	3645.872		
Total	13118899.39	72			

a. R Squared = .987 (Adjusted R Squared = .980)

Estimated Marginal Means

1. Grand Mean

Dependent Variable: Ascorbate.Per_Ro

Mean	Std. Error	95% Confidence Interval	
		Lower Bound	Upper Bound
399.709	7.116	385.402	414.017

2. FC_Levels

Dependent Variable: Ascorbate.Per_Ro

FC_Levels	Mean	Std. Error	95% Confidence Interval	
			Lower Bound	Upper Bound
FFC	329.941	14.232	301.325	358.556
75%FC	337.507	14.232	308.891	366.122
50%FC	436.497	14.232	407.881	465.112
25%FC	494.893	14.232	466.278	523.509

3. NPs

Dependent Variable: Ascorbate.Per_Ro

NPs	Mean	Std. Error	95% Confidence Interval	
			Lower Bound	Upper Bound
Cont	304.582	17.431	269.535	339.628
Water sprayed	215.796	17.431	180.749	250.842
50NP	408.610	17.431	373.564	443.656
150NP	412.128	17.431	377.082	447.175
300NP	454.178	17.431	419.131	489.224
600NP	602.963	17.431	567.916	638.009

4. FC_Levels * NPs

Dependent Variable: Ascorbate.Per_Ro

FC_Levels	NPs	Mean	Std. Error	95% Confidence Interval	
				Lower Bound	Upper Bound
FFC	Cont	217.383	34.861	147.291	287.476
	Water sprayed	171.173	34.861	101.081	241.266
	50NP	360.317	34.861	290.224	430.409
	150NP	366.620	34.861	296.527	436.713
	300NP	386.927	34.861	316.834	457.019
	600NP	477.223	34.861	407.131	547.316
75%FC	Cont	240.880	34.861	170.787	310.973
	Water sprayed	193.303	34.861	123.211	263.396
	50NP	332.650	34.861	262.557	402.743
	150NP	321.090	34.861	250.997	391.183
	300NP	409.667	34.861	339.574	479.759
	600NP	527.450	34.861	457.357	597.543
50%FC	Cont	337.387	34.861	267.294	407.479
	Water sprayed	243.037	34.861	172.944	313.129
	50NP	444.697	34.861	374.604	514.789
	150NP	464.540	34.861	394.447	534.633
	300NP	474.420	34.861	404.327	544.513
	600NP	654.900	34.861	584.807	724.993
25%FC	Cont	422.677	34.861	352.584	492.769
	Water sprayed	255.670	34.861	185.577	325.763
	50NP	496.777	34.861	426.684	566.869
	150NP	496.263	34.861	426.171	566.356
	300NP	545.697	34.861	475.604	615.789
	600NP	752.277	34.861	682.184	822.369

Post Hoc Tests

FC_Levels

Homogeneous Subsets

Ascorbate.Per_Ro

Duncan[a,b]

FC_Levels	N	Subset		
		1	2	3
FFC	18	329.9406		
75%FC	18	337.5067		
50%FC	18		436.4967	
25%FC	18			494.8933
Sig.		.709	1.000	1.000

Means for groups in homogeneous subsets are displayed.
Based on observed means.
The error term is Mean Square(Error) = 3645.872.

a. Uses Harmonic Mean Sample Size = 18.000.

b. Alpha = .05.

NPs

Homogeneous Subsets

Ascorbate.Per_Ro

Duncan[a,b]

NPs	N	Subset			
		1	2	3	4
Water sprayed	12	215.7958			
Cont	12		304.5817		
50NP	12			408.6100	
150NP	12			412.1283	
300NP	12			454.1775	
600NP	12				602.9625
Sig.		1.000	1.000	.086	1.000

Means for groups in homogeneous subsets are displayed.
Based on observed means.
The error term is Mean Square(Error) = 3645.872.

a. Uses Harmonic Mean Sample Size = 12.000.

b. Alpha = .05.

Profile Plots

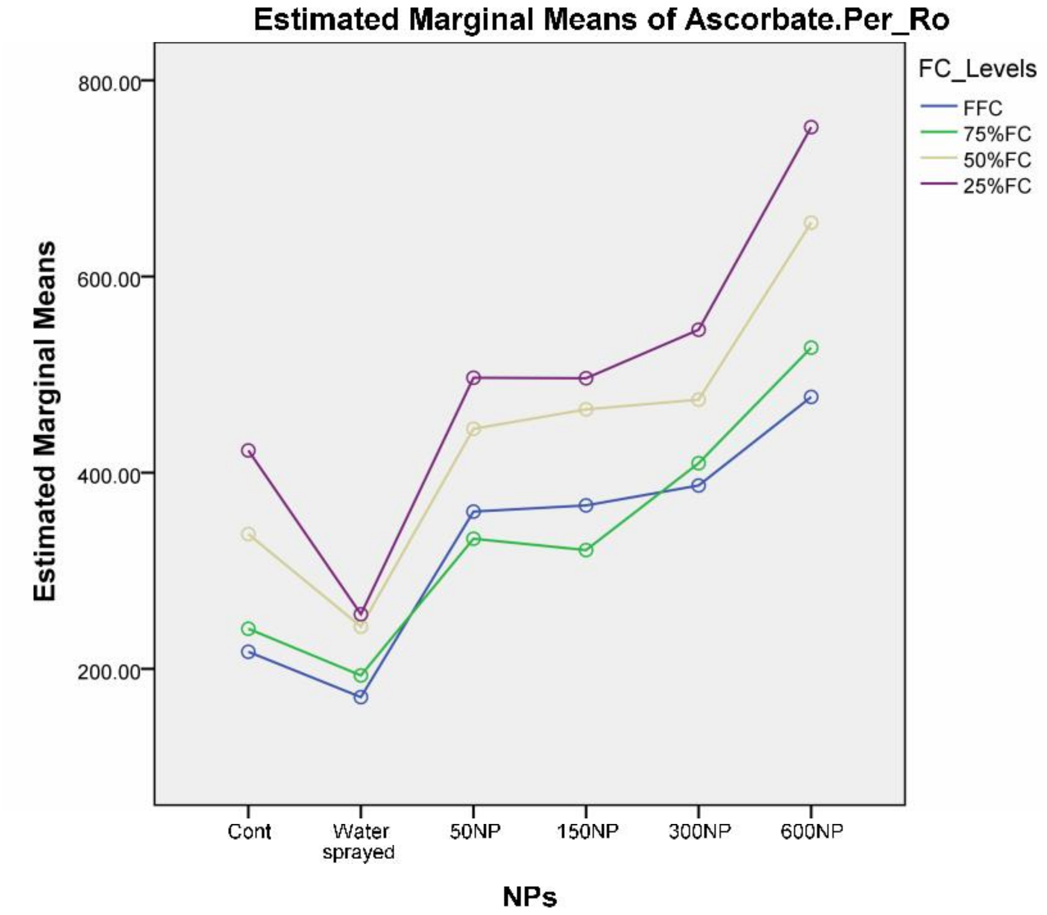


```
UNIANOVA Ascorbate.Per_Ro BY FC_LevOP OPs
  /METHOD=SSTYPE(3)
  /INTERCEPT=EXCLUDE
  /SAVE=SEPRED
  /POSTHOC=FC_LevOP OPs(DUNCAN)
  /PLOT=PROFILE(OPs*FC_LevOP)
  /EMMEANS=TABLES(OVERALL)
  /EMMEANS=TABLES(FC_LevOP)
  /EMMEANS=TABLES(OPs)
  /EMMEANS=TABLES(FC_LevOP*OPs)
  /PRINT=DESCRIPTIVE
  /CRITERIA=ALPHA(.05)
  /DESIGN=FC_LevOP OPs FC_LevOP*OPs.
```

Univariate Analysis of Variance

```
[DataSet1] E:\Amna M Sc\Paper\Two Way ANOVA H.sav
```

Between-Subjects Factors

		Value Label	N
FC_LevOP	1.00	FFC	18
	2.00	75%FC	18
	3.00	50%FC	18
	4.00	25%FC	18
OPs	1.00	Cont	12
	2.00	Water sprayed	12
	3.00	50OPs	12
	4.00	150OPs	12
	5.00	300OPs	12
	6.00	600OPs	12

Descriptive Statistics

Dependent Variable: Ascorbate.Per_Ro

FC_LevOP	OPs	Mean	Std. Deviation	N
FFC	Cont	217.3833	42.41875	3
	Water sprayed	171.1733	38.03500	3
	50OPs	194.6767	10.87151	3
	150OPs	198.7167	23.96251	3
	300OPs	257.4400	34.33000	3
	600OPs	249.7433	30.89401	3
	Total	214.8556	41.28886	18
75%FC	Cont	240.8800	64.93510	3
	Water sprayed	193.3033	9.84382	3
	50OPs	173.0800	35.22440	3
	150OPs	234.7733	22.59172	3
	300OPs	259.8600	25.72095	3
	600OPs	281.0167	46.83500	3
	Total	230.4856	50.00069	18
50%FC	Cont	337.3867	40.51883	3
	Water sprayed	243.0367	25.31109	3
	50OPs	189.5933	31.15854	3
	150OPs	279.8567	13.32500	3
	300OPs	275.6367	82.81397	3
	600OPs	367.9167	63.13185	3
	Total	282.2378	72.84268	18
25%FC	Cont	422.6767	58.85663	3
	Water sprayed	255.6700	30.83091	3
	50OPs	299.6467	42.68173	3
	150OPs	341.5300	44.97728	3
	300OPs	383.2600	33.77358	3
	600OPs	412.2400	28.56152	3
	Total	352.5039	70.96831	18

Descriptive Statistics

Dependent Variable: Ascorbate.Per_Ro

FC_LevOP	OPs	Mean	Std. Deviation	N
Total	Cont	304.5817	96.43450	12
	Water sprayed	215.7958	43.43940	12
	50OPs	214.2492	58.95988	12
	150OPs	263.7192	60.83105	12
	300OPs	294.0492	68.81993	12
	600OPs	327.7292	78.02190	12
	Total	270.0207	80.08507	72

Tests of Between-Subjects Effects

Dependent Variable: Ascorbate.Per_Ro

Source	Type III Sum of Squares	df	Mean Square	F	Sig.
Model	5626008.64[a]	24	234417.027	142.497	.000
FC_LevOP	208061.176	3	69353.725	42.159	.000
OPs	134311.250	5	26862.250	16.329	.000
FC_LevOP * OPs	34031.579	15	2268.772	1.379	.196
Error	78962.914	48	1645.061		
Total	5704971.550	72			

a. R Squared = .986 (Adjusted R Squared = .979)

Estimated Marginal Means

1. Grand Mean

Dependent Variable: Ascorbate.Per_Ro

Mean	Std. Error	95% Confidence Interval	
		Lower Bound	Upper Bound
270.021	4.780	260.410	279.631

2. FC_LevOP

Dependent Variable: Ascorbate.Per_Ro

FC_LevOP	Mean	Std. Error	95% Confidence Interval	
			Lower Bound	Upper Bound
FFC	214.856	9.560	195.634	234.077
75%FC	230.486	9.560	211.264	249.707
50%FC	282.238	9.560	263.016	301.459
25%FC	352.504	9.560	333.282	371.725

3. OPs

Dependent Variable: Ascorbate.Per_Ro

OPs	Mean	Std. Error	95% Confidence Interval Lower Bound	Upper Bound
Cont	304.582	11.708	281.040	328.123
Water sprayed	215.796	11.708	192.254	239.337
50OPs	214.249	11.708	190.708	237.791
150OPs	263.719	11.708	240.178	287.261
300OPs	294.049	11.708	270.508	317.591
600OPs	327.729	11.708	304.188	351.271

4. FC_LevOP * OPs

Dependent Variable: Ascorbate.Per_Ro

FC_LevOP	OPs	Mean	Std. Error	95% Confidence Interval Lower Bound	Upper Bound
FFC	Cont	217.383	23.417	170.300	264.466
	Water sprayed	171.173	23.417	124.090	218.256
	50OPs	194.677	23.417	147.594	241.760
	150OPs	198.717	23.417	151.634	245.800
	300OPs	257.440	23.417	210.357	304.523
	600OPs	249.743	23.417	202.660	296.826
75%FC	Cont	240.880	23.417	193.797	287.963
	Water sprayed	193.303	23.417	146.220	240.386
	50OPs	173.080	23.417	125.997	220.163
	150OPs	234.773	23.417	187.690	281.856
	300OPs	259.860	23.417	212.777	306.943
	600OPs	281.017	23.417	233.934	328.100
50%FC	Cont	337.387	23.417	290.304	384.470
	Water sprayed	243.037	23.417	195.954	290.120
	50OPs	189.593	23.417	142.510	236.676
	150OPs	279.857	23.417	232.774	326.940
	300OPs	275.637	23.417	228.554	322.720
	600OPs	367.917	23.417	320.834	415.000
25%FC	Cont	422.677	23.417	375.594	469.760
	Water sprayed	255.670	23.417	208.587	302.753
	50OPs	299.647	23.417	252.564	346.730
	150OPs	341.530	23.417	294.447	388.613
	300OPs	383.260	23.417	336.177	430.343
	600OPs	412.240	23.417	365.157	459.323

Post Hoc Tests

FC_LevOP

Homogeneous Subsets

Ascorbate.Per_Ro

Duncan[a,b]

FC_LevOP	N	Subset 1	2	3
FFC	18	214.8556		
75%FC	18	230.4856		
50%FC	18		282.2378	
25%FC	18			352.5039
Sig.		.253	1.000	1.000

Means for groups in homogeneous subsets are displayed.
Based on observed means.
The error term is Mean Square(Error) = 1645.061.

a. Uses Harmonic Mean Sample Size = 18.000.

b. Alpha = .05.

OPs

Homogeneous Subsets

Ascorbate.Per_Ro

Duncan[a,b]

OPs	N	Subset 1	2	3
50OPs	12	214.2492		
Water sprayed	12	215.7958		
150OPs	12		263.7192	
300OPs	12		294.0492	294.0492
Cont	12			304.5817
600OPs	12			327.7292
Sig.		.926	.073	.059

Means for groups in homogeneous subsets are displayed.
Based on observed means.
The error term is Mean Square(Error) = 1645.061.

a. Uses Harmonic Mean Sample Size = 12.000.

b. Alpha = .05.

Profile Plots

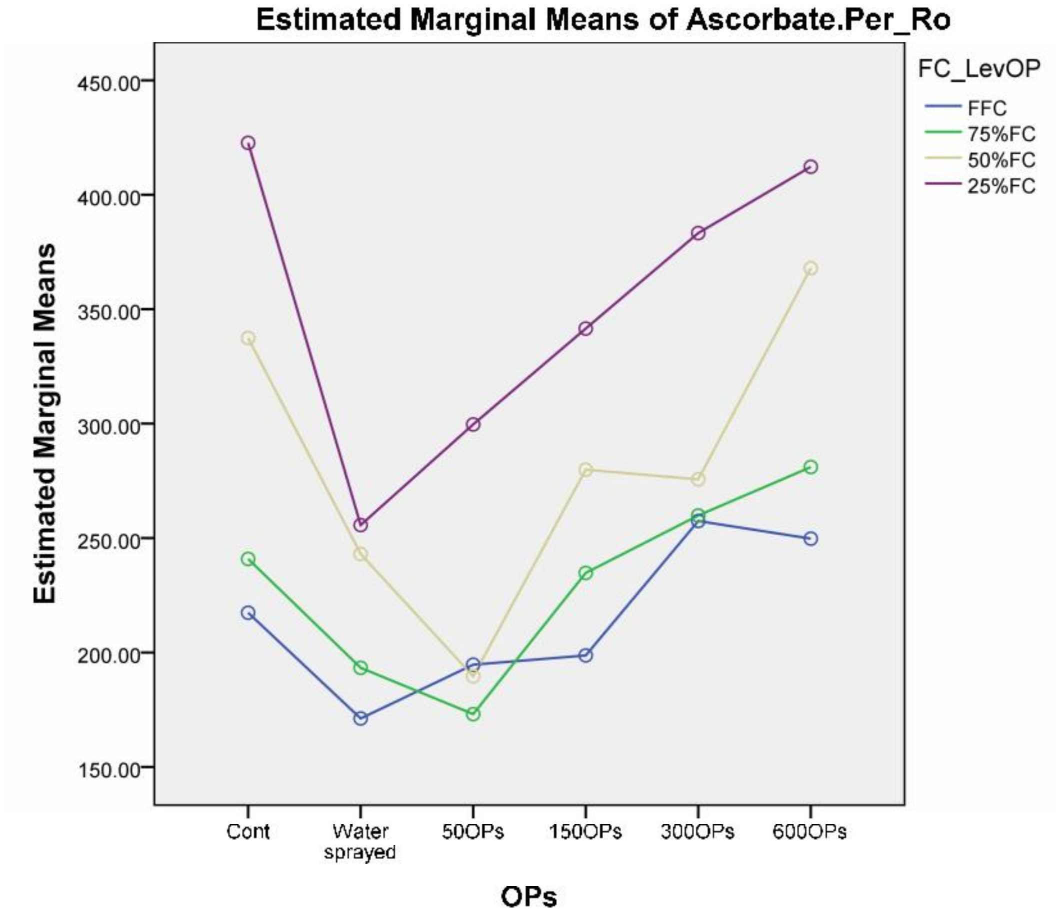


```
GET
  FILE='E:\Amna M Sc\Paper\Two Way ANOVA H.sav'.
DATASET NAME DataSet1 WINDOW=FRONT.
UNIANOVA LIP_PER_Ro BY FC_Levels NPs
  /METHOD=SSTYPE(3)
  /INTERCEPT=EXCLUDE
  /POSTHOC=FC_Levels NPs(DUNCAN)
  /PLOT=PROFILE(NPs*FC_Levels)
  /EMMEANS=TABLES(OVERALL)
  /EMMEANS=TABLES(FC_Levels)
  /EMMEANS=TABLES(NPs)
  /EMMEANS=TABLES(FC_Levels*NPs)
  /PRINT=DESCRIPTIVE
  /CRITERIA=ALPHA(.05)
  /DESIGN=FC_Levels NPs FC_Levels*NPs.
```

Univariate Analysis of Variance

```
[DataSet1] E:\Amna M Sc\Paper\Two Way ANOVA H.sav
```

Between-Subjects Factors

		Value Label	N
FC_Levels	1	FFC	18
	2	75%FC	18
	3	50%FC	18
	4	25%FC	18
NPs	1	Cont	12
	2	Water sprayed	12
	3	50NP	12
	4	150NP	12
	5	300NP	12
	6	600NP	12

Descriptive Statistics

Dependent Variable: LIP_PER_Ro

FC_Levels	NPs	Mean	Std. Deviation	N
FFC	Cont	77.7067	.58046	3
	Water sprayed	81.0167	1.66785	3
	50NP	56.1367	6.73451	3
	150NP	25.4767	.79664	3
	300NP	51.0800	5.92317	3
	600NP	129.6367	10.52922	3
	Total	70.1756	33.60575	18
75%FC	Cont	52.6100	2.60179	3
	Water sprayed	79.3633	2.54744	3
	50NP	43.0533	.21939	3
	150NP	43.3133	1.54111	3
	300NP	52.2267	7.23114	3
	600NP	111.4667	5.49058	3
	Total	63.6722	25.51445	18
50%FC	Cont	35.5400	1.37982	3
	Water sprayed	85.2267	2.50708	3
	50NP	79.3600	2.60179	3
	150NP	54.2667	4.65167	3
	300NP	72.8667	1.96105	3
	600NP	87.8967	5.65221	3
	Total	69.1928	19.37562	18
25%FC	Cont	51.2100	1.75120	3
	Water sprayed	88.1533	2.87210	3
	50NP	69.4267	3.97598	3
	150NP	54.1367	2.54543	3
	300NP	63.6933	2.96837	3
	600NP	56.6867	5.94535	3
	Total	63.8844	13.14428	18

Descriptive Statistics

Dependent Variable: LIP_PER_Ro

FC_Levels	NPs	Mean	Std. Deviation	N
Total	Cont	54.2667	15.84235	12
	Water sprayed	83.4400	4.17034	12
	50NP	61.9942	14.72659	12
	150NP	44.2983	12.49149	12
	300NP	59.9667	10.25846	12
	600NP	96.4217	29.17037	12
	Total	66.7313	23.80134	72

Tests of Between-Subjects Effects

Dependent Variable: LIP_PER_Ro

Source	Type III Sum of Squares	df	Mean Square	F	Sig.
Model	359955.413[a]	24	14998.142	811.945	.000
FC_Levels	636.918	3	212.306	11.493	.000
NPs	22650.052	5	4530.010	245.238	.000
FC_Levels * NPs	16048.143	15	1069.876	57.919	.000
Error	886.649	48	18.472		
Total	360842.062	72			

a. R Squared = .998 (Adjusted R Squared = .996)

Estimated Marginal Means

1. Grand Mean

Dependent Variable: LIP_PER_Ro

		95% Confidence Interval	
Mean	Std. Error	Lower Bound	Upper Bound
66.731	.507	65.713	67.750

2. FC_Levels

Dependent Variable: LIP_PER_Ro

			95% Confidence Interval	
FC_Levels	Mean	Std. Error	Lower Bound	Upper Bound
FFC	70.176	1.013	68.139	72.212
75%FC	63.672	1.013	61.635	65.709
50%FC	69.193	1.013	67.156	71.230
25%FC	63.884	1.013	61.848	65.921

3. NPs

Dependent Variable: LIP_PER_Ro

NPs	Mean	Std. Error	95% Confidence Interval	
			Lower Bound	Upper Bound
Cont	54.267	1.241	51.772	56.761
Water sprayed	83.440	1.241	80.945	85.935
50NP	61.994	1.241	59.500	64.489
150NP	44.298	1.241	41.804	46.793
300NP	59.967	1.241	57.472	62.461
600NP	96.422	1.241	93.927	98.916

4. FC_Levels * NPs

Dependent Variable: LIP_PER_Ro

FC_Levels	NPs	Mean	Std. Error	95% Confidence Interval	
				Lower Bound	Upper Bound
FFC	Cont	77.707	2.481	72.718	82.696
	Water sprayed	81.017	2.481	76.028	86.006
	50NP	56.137	2.481	51.148	61.126
	150NP	25.477	2.481	20.488	30.466
	300NP	51.080	2.481	46.091	56.069
	600NP	129.637	2.481	124.648	134.626
75%FC	Cont	52.610	2.481	47.621	57.599
	Water sprayed	79.363	2.481	74.374	84.352
	50NP	43.053	2.481	38.064	48.042
	150NP	43.313	2.481	38.324	48.302
	300NP	52.227	2.481	47.238	57.216
	600NP	111.467	2.481	106.478	116.456
50%FC	Cont	35.540	2.481	30.551	40.529
	Water sprayed	85.227	2.481	80.238	90.216
	50NP	79.360	2.481	74.371	84.349
	150NP	54.267	2.481	49.278	59.256
	300NP	72.867	2.481	67.878	77.856
	600NP	87.897	2.481	82.908	92.886
25%FC	Cont	51.210	2.481	46.221	56.199
	Water sprayed	88.153	2.481	83.164	93.142
	50NP	69.427	2.481	64.438	74.416
	150NP	54.137	2.481	49.148	59.126
	300NP	63.693	2.481	58.704	68.682
	600NP	56.687	2.481	51.698	61.676

Post Hoc Tests

FC_Levels

Homogeneous Subsets

LIP_PER_Ro

Duncan[a,b]

FC_Levels	N	Subset	
		1	2
75%FC	18	63.6722	
25%FC	18	63.8844	
50%FC	18		69.1928
FFC	18		70.1756
Sig.		.883	.496

Means for groups in homogeneous subsets are displayed.
Based on observed means.
The error term is Mean Square(Error) = 18.472.

a. Uses Harmonic Mean Sample Size = 18.000.

b. Alpha = .05.

NPs

Homogeneous Subsets

LIP_PER_Ro

Duncan[a,b]

NPs	N	Subset				
		1	2	3	4	5
150NP	12	44.2983				
Cont	12		54.2667			
300NP	12			59.9667		
50NP	12			61.9942		
Water sprayed	12				83.4400	
600NP	12					96.4217
Sig.		1.000	1.000	.254	1.000	1.000

Means for groups in homogeneous subsets are displayed.
Based on observed means.
The error term is Mean Square(Error) = 18.472.

a. Uses Harmonic Mean Sample Size = 12.000.

b. Alpha = .05.

Profile Plots

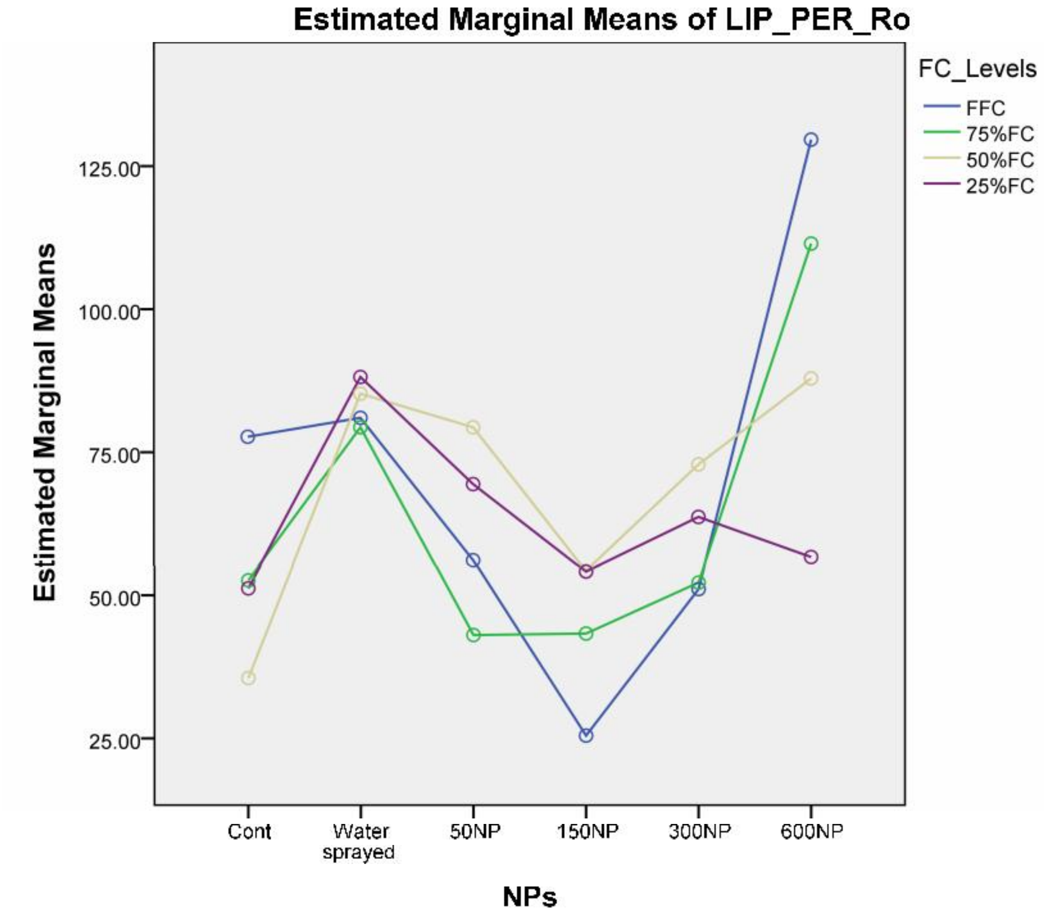


```
UNIANOVA LIP_PER_Ro BY FC_LevOP OPs
  /METHOD=SSTYPE(3)
  /INTERCEPT=EXCLUDE
  /POSTHOC=FC_LevOP OPs(DUNCAN)
  /PLOT=PROFILE(OPs*FC_LevOP)
  /EMMEANS=TABLES(OVERALL)
  /EMMEANS=TABLES(FC_LevOP)
  /EMMEANS=TABLES(OPs)
  /EMMEANS=TABLES(FC_LevOP*OPs)
  /PRINT=DESCRIPTIVE
  /CRITERIA=ALPHA(.05)
  /DESIGN=FC_LevOP OPs FC_LevOP*OPs.
```

Univariate Analysis of Variance

```
[DataSet1] E:\Amna M Sc\Paper\Two Way ANOVA H.sav
```

Between-Subjects Factors

		Value Label	N
FC_LevOP	1.00	FFC	18
	2.00	75%FC	18
	3.00	50%FC	18
	4.00	25%FC	18
OPs	1.00	Cont	12
	2.00	Water sprayed	12
	3.00	50OPs	12
	4.00	150OPs	12
	5.00	300OPs	12
	6.00	600OPs	12

Descriptive Statistics

Dependent Variable: LIP_PER_Ro

FC_LevOP	OPs	Mean	Std. Deviation	N
FFC	Cont	77.7067	.58046	3
	Water sprayed	81.0167	1.66785	3
	50OPs	82.9300	2.02022	3
	150OPs	84.8400	6.00812	3
	300OPs	92.7400	.96161	3
	600OPs	63.1867	1.23087	3
	Total	80.4033	9.51298	18
75%FC	Cont	52.6100	2.60179	3
	Water sprayed	79.3633	2.54744	3
	50OPs	77.5800	1.66556	3
	150OPs	78.7267	11.35610	3
	300OPs	98.0867	1.88598	3
	600OPs	75.6667	2.02211	3
	Total	77.0056	14.25788	18
50%FC	Cont	35.5400	1.37982	3
	Water sprayed	85.2267	2.50708	3
	50OPs	98.2167	3.98791	3
	150OPs	94.5233	3.06308	3
	300OPs	103.3100	2.86721	3
	600OPs	68.2800	1.76669	3
	Total	80.8494	23.95970	18
25%FC	Cont	51.2100	1.75120	3
	Water sprayed	88.1533	2.87210	3
	50OPs	90.7867	5.33233	3
	150OPs	124.2033	1.66785	3
	300OPs	113.3767	.79664	3
	600OPs	56.5600	8.93940	3
	Total	87.3817	27.81509	18

Descriptive Statistics

Dependent Variable: LIP_PER_Ro

FC_LevOP	OPs	Mean	Std. Deviation	N
Total	Cont	54.2667	15.84235	12
	Water sprayed	83.4400	4.17034	12
	50OPs	87.3783	8.72316	12
	150OPs	95.5733	19.10218	12
	300OPs	101.8783	8.10789	12
	600OPs	65.9233	8.33514	12
	Total	81.4100	20.18261	72

Tests of Between-Subjects Effects

Dependent Variable: LIP_PER_Ro

Source	Type III Sum of Squares	df	Mean Square	F	Sig.
Model	505374.784[a]	24	21057.283	1379.777	.000
FC_LevOP	1014.976	3	338.325	22.169	.000
OPs	19630.704	5	3926.141	257.260	.000
FC_LevOP * OPs	7542.762	15	502.851	32.949	.000
Error	732.546	48	15.261		
Total	506107.330	72			

a. R Squared = .999 (Adjusted R Squared = .998)

Estimated Marginal Means

1. Grand Mean

Dependent Variable: LIP_PER_Ro

Mean	Std. Error	95% Confidence Interval	
		Lower Bound	Upper Bound
81.410	.460	80.484	82.336

2. FC_LevOP

Dependent Variable: LIP_PER_Ro

FC_LevOP	Mean	Std. Error	95% Confidence Interval	
			Lower Bound	Upper Bound
FFC	80.403	.921	78.552	82.255
75%FC	77.006	.921	75.154	78.857
50%FC	80.849	.921	78.998	82.701
25%FC	87.382	.921	85.530	89.233

3. OPs

Dependent Variable: LIP_PER_Ro

OPs	Mean	Std. Error	95% Confidence Interval	
			Lower Bound	Upper Bound
Cont	54.267	1.128	51.999	56.534
Water sprayed	83.440	1.128	81.173	85.707
50OPs	87.378	1.128	85.111	89.646
150OPs	95.573	1.128	93.306	97.841
300OPs	101.878	1.128	99.611	104.146
600OPs	65.923	1.128	63.656	68.191

4. FC_LevOP * OPs

Dependent Variable: LIP_PER_Ro

FC_LevOP	OPs	Mean	Std. Error	95% Confidence Interval	
				Lower Bound	Upper Bound
FFC	Cont	77.707	2.255	73.172	82.242
	Water sprayed	81.017	2.255	76.482	85.552
	50OPs	82.930	2.255	78.395	87.465
	150OPs	84.840	2.255	80.305	89.375
	300OPs	92.740	2.255	88.205	97.275
	600OPs	63.187	2.255	58.652	67.722
75%FC	Cont	52.610	2.255	48.075	57.145
	Water sprayed	79.363	2.255	74.828	83.898
	50OPs	77.580	2.255	73.045	82.115
	150OPs	78.727	2.255	74.192	83.262
	300OPs	98.087	2.255	93.552	102.622
	600OPs	75.667	2.255	71.132	80.202
50%FC	Cont	35.540	2.255	31.005	40.075
	Water sprayed	85.227	2.255	80.692	89.762
	50OPs	98.217	2.255	93.682	102.752
	150OPs	94.523	2.255	89.988	99.058
	300OPs	103.310	2.255	98.775	107.845
	600OPs	68.280	2.255	63.745	72.815
25%FC	Cont	51.210	2.255	46.675	55.745
	Water sprayed	88.153	2.255	83.618	92.688
	50OPs	90.787	2.255	86.252	95.322
	150OPs	124.203	2.255	119.668	128.738
	300OPs	113.377	2.255	108.842	117.912
	600OPs	56.560	2.255	52.025	61.095

Post Hoc Tests

FC_LevOP

Homogeneous Subsets

LIP_PER_Ro

Duncan[a,b]

FC_LevOP	N	Subset		
		1	2	3
75%FC	18	77.0056		
FFC	18		80.4033	
50%FC	18		80.8494	
25%FC	18			87.3817
Sig.		1.000	.733	1.000

Means for groups in homogeneous subsets are displayed.
Based on observed means.
The error term is Mean Square(Error) = 15.261.

a. Uses Harmonic Mean Sample Size = 18.000.

b. Alpha = .05.

OPs

Homogeneous Subsets

LIP_PER_Ro

Duncan[a,b]

OPs	N	Subset					
		1	2	3	4	5	6
Cont	12	54.2667					
600OPs	12		65.9233				
Water sprayed	12			83.4400			
50OPs	12				87.3783		
150OPs	12					95.5733	
300OPs	12						101.8783
Sig.		1.000	1.000	1.000	1.000	1.000	1.000

Means for groups in homogeneous subsets are displayed.
Based on observed means.
The error term is Mean Square(Error) = 15.261.

a. Uses Harmonic Mean Sample Size = 12.000.

b. Alpha = .05.

Profile Plots

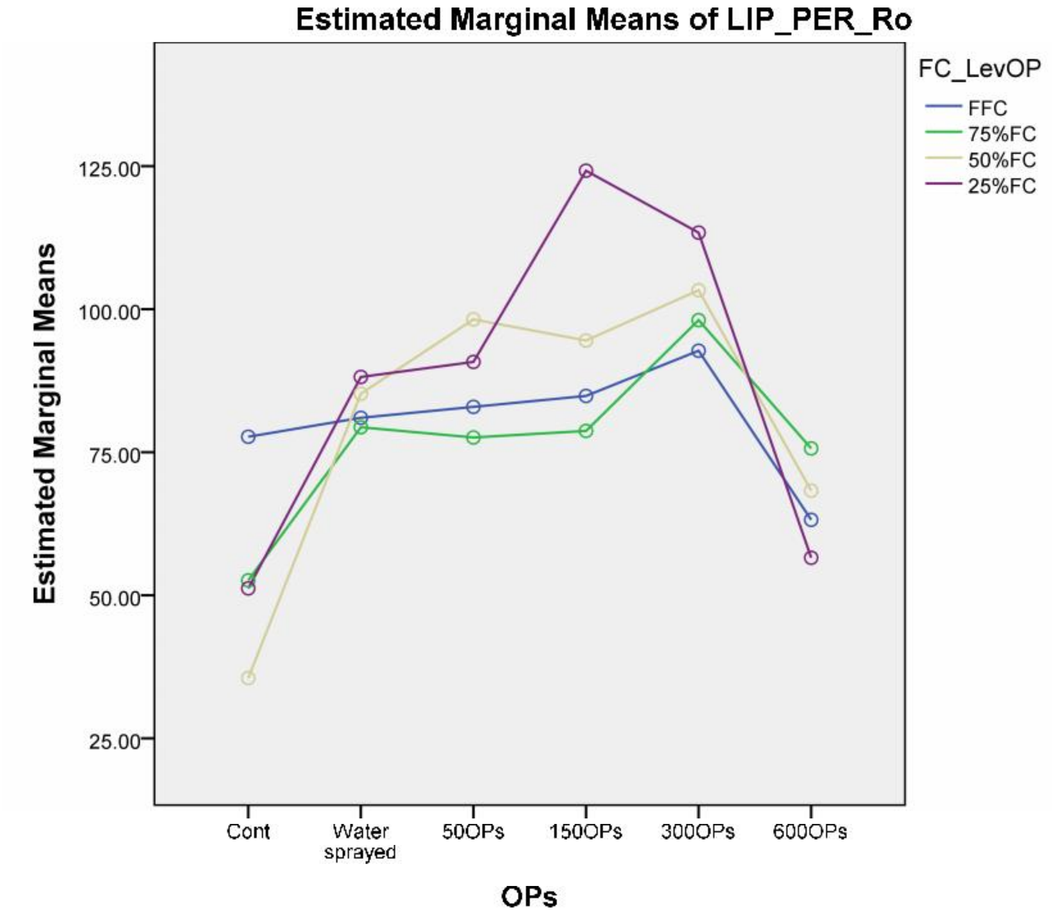


```
UNIANOVA Catalase_Ro BY FC_Levels NPs
  /METHOD=SSTYPE(3)
  /INTERCEPT=EXCLUDE
  /POSTHOC=FC_Levels NPs(DUNCAN)
  /PLOT=PROFILE(NPs*FC_Levels)
  /EMMEANS=TABLES(OVERALL)
  /EMMEANS=TABLES(FC_Levels)
  /EMMEANS=TABLES(NPs)
  /EMMEANS=TABLES(FC_Levels*NPs)
  /PRINT=DESCRIPTIVE
  /CRITERIA=ALPHA(.05)
  /DESIGN=FC_Levels NPs FC_Levels*NPs.
```

Univariate Analysis of Variance

```
[DataSet1] E:\Amna M Sc\Paper\Two Way ANOVA H.sav
```

Between-Subjects Factors

		Value Label	N
FC_Levels	1	FFC	18
	2	75%FC	18
	3	50%FC	18
	4	25%FC	18
NPs	1	Cont	12
	2	Water sprayed	12
	3	50NP	12
	4	150NP	12
	5	300NP	12
	6	600NP	12

Descriptive Statistics

Dependent Variable: Catalase_Ro

FC_Levels	NPs	Mean	Std. Deviation	N
FFC	Cont	1.7292	.74876	3
	Water sprayed	1.6138	.69878	3
	50NP	1.9938	.86336	3
	150NP	2.1602	.74832	3
	300NP	2.0867	.72285	3
	600NP	2.4295	1.05200	3
	Total	2.0022	.73914	18
75%FC	Cont	4.5085	.78090	3
	Water sprayed	2.5326	.62665	3
	50NP	3.6032	.78011	3
	150NP	4.3474	.75300	3
	300NP	2.9794	.64506	3
	600NP	4.4331	1.09691	3
	Total	3.7340	1.03126	18
50%FC	Cont	6.6062	.71514	3
	Water sprayed	4.9210	.60882	3
	50NP	5.0208	.79057	3
	150NP	6.7846	.83938	3
	300NP	7.0445	.87152	3
	600NP	7.9040	1.24455	3
	Total	6.3802	1.32632	18
25%FC	Cont	8.4939	1.41566	3
	Water sprayed	6.4223	1.28445	3
	50NP	8.8330	.80522	3
	150NP	8.3106	1.66212	3
	300NP	10.3569	1.05522	3
	600NP	15.6506	1.59456	3
	Total	9.6779	3.19555	18

Descriptive Statistics

Dependent Variable: Catalase_Ro

FC_Levels	NPs	Mean	Std. Deviation	N
Total	Cont	5.3344	2.75039	12
	Water sprayed	3.8724	2.11696	12
	50NP	4.8627	2.73147	12
	150NP	5.4007	2.61407	12
	300NP	5.6169	3.53422	12
	600NP	7.6043	5.37507	12
	Total	5.4486	3.42971	72

Tests of Between-Subjects Effects

Dependent Variable: Catalase_Ro

Source	Type III Sum of Squares	df	Mean Square	F	Sig.
Model	2926.579[a]	24	121.941	127.121	.000
FC_Levels	604.299	3	201.433	209.990	.000
NPs	90.220	5	18.044	18.810	.000
FC_Levels * NPs	94.605	15	6.307	6.575	.000
Error	46.044	48	.959		
Total	2972.623	72			

a. R Squared = .985 (Adjusted R Squared = .977)

Estimated Marginal Means

1. Grand Mean

Dependent Variable: Catalase_Ro

Mean	Std. Error	95% Confidence Interval	
		Lower Bound	Upper Bound
5.449	.115	5.216	5.681

2. FC_Levels

Dependent Variable: Catalase_Ro

FC_Levels	Mean	Std. Error	95% Confidence Interval	
			Lower Bound	Upper Bound
FFC	2.002	.231	1.538	2.466
75%FC	3.734	.231	3.270	4.198
50%FC	6.380	.231	5.916	6.844
25%FC	9.678	.231	9.214	10.142

3. NPs

Dependent Variable: Catalase_Ro

NPs	Mean	Std. Error	95% Confidence Interval	
			Lower Bound	Upper Bound
Cont	5.334	.283	4.766	5.903
Water sprayed	3.872	.283	3.304	4.441
50NP	4.863	.283	4.294	5.431
150NP	5.401	.283	4.832	5.969
300NP	5.617	.283	5.048	6.185
600NP	7.604	.283	7.036	8.173

4. FC_Levels * NPs

Dependent Variable: Catalase_Ro

FC_Levels	NPs	Mean	Std. Error	95% Confidence Interval	
				Lower Bound	Upper Bound
FFC	Cont	1.729	.565	.592	2.866
	Water sprayed	1.614	.565	.477	2.751
	50NP	1.994	.565	.857	3.131
	150NP	2.160	.565	1.023	3.297
	300NP	2.087	.565	.950	3.224
	600NP	2.429	.565	1.293	3.566
75%FC	Cont	4.509	.565	3.372	5.645
	Water sprayed	2.533	.565	1.396	3.670
	50NP	3.603	.565	2.466	4.740
	150NP	4.347	.565	3.210	5.484
	300NP	2.979	.565	1.842	4.116
	600NP	4.433	.565	3.296	5.570
50%FC	Cont	6.606	.565	5.469	7.743
	Water sprayed	4.921	.565	3.784	6.058
	50NP	5.021	.565	3.884	6.158
	150NP	6.785	.565	5.648	7.922
	300NP	7.044	.565	5.908	8.181
	600NP	7.904	.565	6.767	9.041
25%FC	Cont	8.494	.565	7.357	9.631
	Water sprayed	6.422	.565	5.285	7.559
	50NP	8.833	.565	7.696	9.970
	150NP	8.311	.565	7.174	9.448
	300NP	10.357	.565	9.220	11.494
	600NP	15.651	.565	14.514	16.788

Post Hoc Tests

FC_Levels

Homogeneous Subsets

Catalase_Ro

Duncan[a,b]

FC_Levels	N	Subset 1	2	3	4
FFC	18	2.0022			
75%FC	18		3.7340		
50%FC	18			6.3802	
25%FC	18				9.6779
Sig.		1.000	1.000	1.000	1.000

Means for groups in homogeneous subsets are displayed.
Based on observed means.
The error term is Mean Square(Error) = .959.

a. Uses Harmonic Mean Sample Size = 18.000.

b. Alpha = .05.

NPs

Homogeneous Subsets

Catalase_Ro

Duncan[a,b]

NPs	N	Subset 1	2	3
Water sprayed	12	3.8724		
50NP	12		4.8627	
Cont	12		5.3344	
150NP	12		5.4007	
300NP	12		5.6169	
600NP	12			7.6043
Sig.		1.000	.090	1.000

Means for groups in homogeneous subsets are displayed.
Based on observed means.
The error term is Mean Square(Error) = .959.

a. Uses Harmonic Mean Sample Size = 12.000.

b. Alpha = .05.

Profile Plots

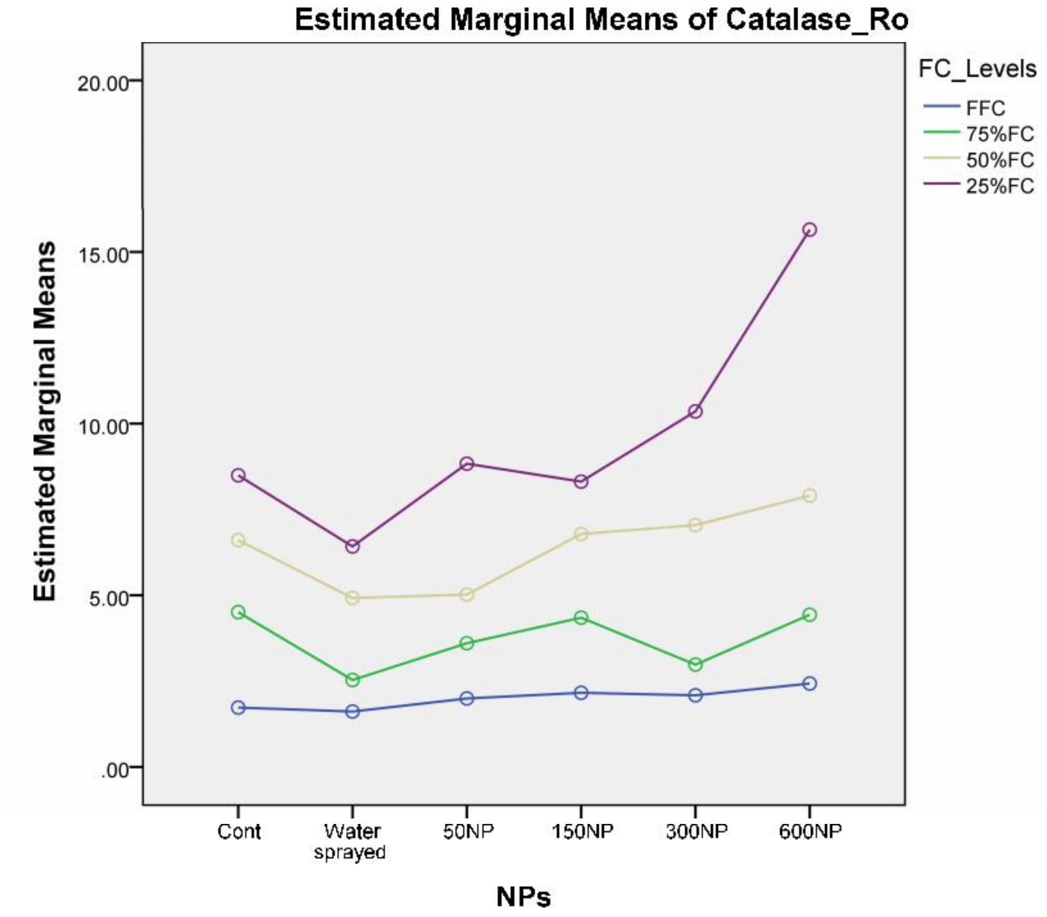


```
UNIANOVA Catalase_Ro BY FC_LevOP OPs
  /METHOD=SSTYPE(3)
  /INTERCEPT=EXCLUDE
  /POSTHOC=FC_LevOP OPs(DUNCAN)
  /PLOT=PROFILE(OPs*FC_LevOP)
  /EMMEANS=TABLES(OVERALL)
  /PRINT=DESCRIPTIVE
  /CRITERIA=ALPHA(.05)
  /DESIGN=FC_LevOP OPs FC_LevOP*OPs.
```

Univariate Analysis of Variance

[DataSet1] E:\Amna M Sc\Paper\Two Way ANOVA H.sav

Between-Subjects Factors

		Value Label	N
FC_LevOP	1.00	FFC	18
	2.00	75%FC	18
	3.00	50%FC	18
	4.00	25%FC	18
OPs	1.00	Cont	12
	2.00	Water sprayed	12
	3.00	50OPs	12
	4.00	150OPs	12
	5.00	300OPs	12
	6.00	600OPs	12

Descriptive Statistics

Dependent Variable: Catalase_Ro

FC_LevOP	OPs	Mean	Std. Deviation	N
FFC	Cont	1.7292	.74876	3
	Water sprayed	1.6138	.69878	3
	50OPs	2.7974	.69217	3
	150OPs	3.3278	.57639	3
	300OPs	1.8203	.63056	3
	600OPs	3.7840	.65541	3
	Total	2.5121	1.03367	18
75%FC	Cont	4.5085	.78090	3
	Water sprayed	2.5326	.62665	3
	50OPs	3.1066	.48916	3
	150OPs	3.8733	.47920	3
	300OPs	4.7247	.81835	3
	600OPs	4.9674	.86039	3
	Total	3.9522	1.07877	18
50%FC	Cont	6.6062	.71514	3
	Water sprayed	4.9210	.60882	3
	50OPs	5.3975	.54993	3
	150OPs	3.9576	.48962	3
	300OPs	4.8723	.84392	3
	600OPs	7.4495	.92164	3
	Total	5.5340	1.33680	18
25%FC	Cont	8.4939	1.41566	3
	Water sprayed	6.4223	1.28445	3
	50OPs	7.9152	.62316	3
	150OPs	4.3468	.47055	3
	300OPs	7.5043	.81237	3
	600OPs	10.4933	.90875	3
	Total	7.5293	2.10262	18

Descriptive Statistics

Dependent Variable: Catalase_Ro

FC_LevOP	OPs	Mean	Std. Deviation	N
Total	Cont	5.3344	2.75039	12
	Water sprayed	3.8724	2.11696	12
	50OPs	4.8042	2.20817	12
	150OPs	3.8764	.57465	12
	300OPs	4.7304	2.20392	12
	600OPs	6.6736	2.78059	12
	Total	4.8819	2.35552	72

Tests of Between-Subjects Effects

Dependent Variable: Catalase_Ro

Source	Type III Sum of Squares	df	Mean Square	F	Sig.
Model	2081.303[a]	24	86.721	145.505	.000
FC_LevOP	250.459	3	83.486	140.077	.000
OPs	65.688	5	13.138	22.043	.000
FC_LevOP * OPs	49.188	15	3.279	5.502	.000
Error	28.608	48	.596		
Total	2109.911	72			

a. R Squared = .986 (Adjusted R Squared = .980)

Estimated Marginal Means

Grand Mean

Dependent Variable: Catalase_Ro

Mean	Std. Error	95% Confidence Interval	
		Lower Bound	Upper Bound
4.882	.091	4.699	5.065

Post Hoc Tests

FC_LevOP

Homogeneous Subsets

Catalase_Ro

Duncan[a,b]

FC_LevOP	N	Subset			
		1	2	3	4
FFC	18	2.5121			
75%FC	18		3.9522		
50%FC	18			5.5340	
25%FC	18				7.5293
Sig.		1.000	1.000	1.000	1.000

Means for groups in homogeneous subsets are displayed.
Based on observed means.
The error term is Mean Square(Error) = .596.

a. Uses Harmonic Mean Sample Size = 18.000.

b. Alpha = .05.

OPs

Homogeneous Subsets

Catalase_Ro

Duncan[a,b]

OPs	N	Subset		
		1	2	3
Water sprayed	12	3.8724		
150OPs	12	3.8764		
300OPs	12		4.7304	
50OPs	12		4.8042	
Cont	12		5.3344	
600OPs	12			6.6736
Sig.		.990	.075	1.000

Means for groups in homogeneous subsets are displayed.
Based on observed means.
The error term is Mean Square(Error) = .596.

a. Uses Harmonic Mean Sample Size = 12.000.

b. Alpha = .05.

Profile Plots

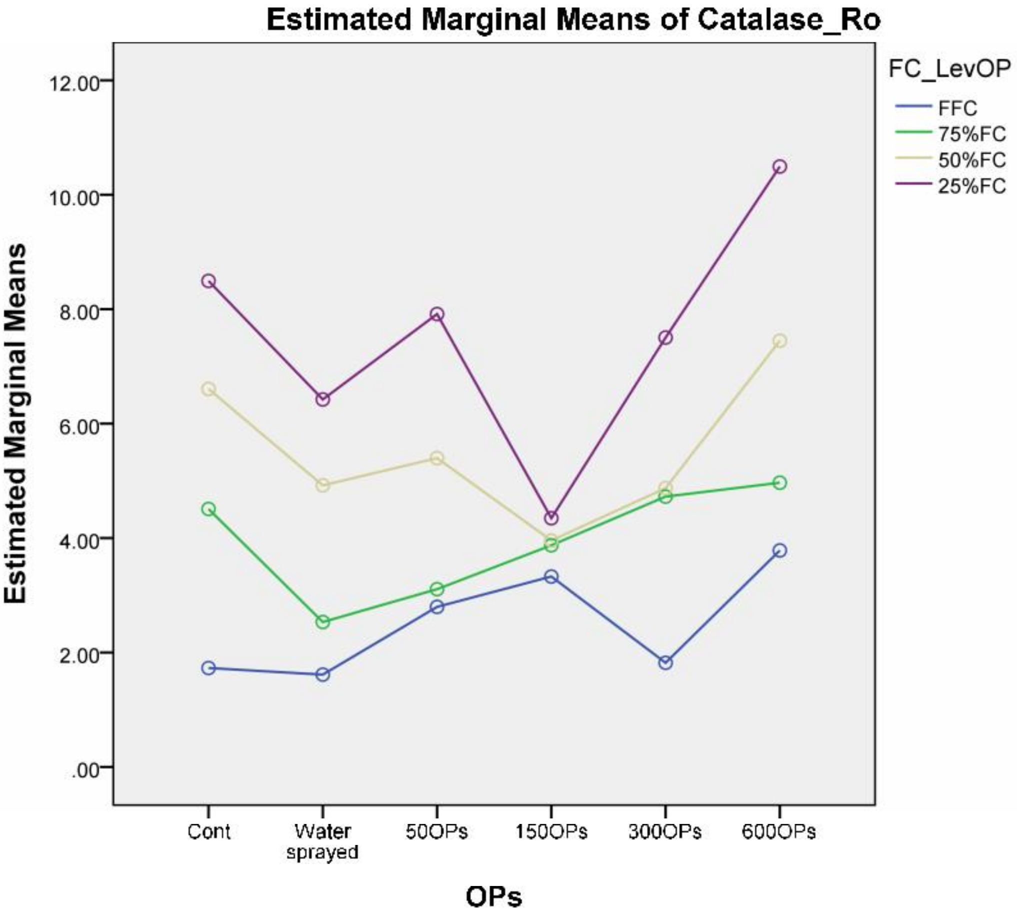
Estimated Marginal Means of Catalase_Ro
FC_LevOP
FFC
75%FC
50%FC
25%FC
Estimated Marginal Means
12.00
10.00
8.00
6.00
4.00
2.00
.00
Cont
Water sprayed
50OPs
150OPs
300OPs
600OPs
OPs
